# Regulation of IL-24/IL-20R2 complex formation using photocaged tyrosines and UV light

**DOI:** 10.3389/fmolb.2023.1214235

**Published:** 2023-07-07

**Authors:** Phuong Ngoc Pham, Jiří Zahradník, Lucie Kolářová, Bohdan Schneider, Gustavo Fuertes

**Affiliations:** ^1^ Laboratory of Biomolecular Recognition, Institute of Biotechnology of the Czech Academy of Sciences, Vestec, Czechia; ^2^ Faculty of Science, Charles University, Prague, Czechia; ^3^ First Faculty of Medicine, BIOCEV Center, Charles University, Prague, Czechia; ^4^ Department of Biomolecular Sciences, Weizmann Institute of Science, Rehovot, Israel

**Keywords:** protein-protein interactions (PPI), interleukin-24, cytokines, optobinders, genetically encoded non-canonical amino acids (ncAA), photocaged proteins, ortho-nitrobenzyltyrosine (NBY), photoxenoprotein engineering

## Abstract

Human interleukin 24 (IL-24) is a multifunctional cytokine that represents an important target for autoimmune diseases and cancer. Since the biological functions of IL-24 depend on interactions with membrane receptors, on-demand regulation of the affinity between IL-24 and its cognate partners offers exciting possibilities in basic research and may have applications in therapy. As a proof-of-concept, we developed a strategy based on recombinant soluble protein variants and genetic code expansion technology to photocontrol the binding between IL-24 and one of its receptors, IL-20R2. Screening of non-canonical *ortho*-nitrobenzyl-tyrosine (NBY) residues introduced at several positions in both partners was done by a combination of biophysical and cell signaling assays. We identified one position for installing NBY, tyrosine70 of IL-20R2, which results in clear impairment of heterocomplex assembly in the dark. Irradiation with 365-nm light leads to decaging and reconstitutes the native tyrosine of the receptor that can then associate with IL-24. Photocaged IL-20R2 may be useful for the spatiotemporal control of the JAK/STAT phosphorylation cascade.

## 1 Introduction

IL-24 is a multifunctional cytokine playing key roles in immune response, host defense, tissue homeostasis, and cell proliferation (Ma et al., 2011; Rutz et al., 2014; Liu et al., 2023). It is a member of a broader family of IL-10 related cytokines including IL-10, IL-19, IL-20, IL-22, IL-24, IL-26, IL-28 and IL-29 (Akdis et al., 2011). An increase in the expression levels of IL-24 is connected to autoimmune diseases, such as psoriasis (Kragstrup et al., 2008), inflammatory bowel disease (Andoh et al., 2009), and rheumatoid arthritis (de Melo et al., 2012). Moreover, a large number of studies suggest anticancer properties for IL-24, such as stimulation of apoptosis and autophagy, or inhibition of angiogenesis, invasion and metastasis (Menezes et al., 2018). Given the anti-oncogenesis effects of IL-24, it became a pharmacological target and even reached phase I clinical trials (Cunningham et al., 2005). Accumulated evidence strengthened the concept of the “bystander effect” according to which secreted IL-24, either in normal or cancer cells, induces tumor apoptosis in presence of IL-20/IL-22 receptors (Su et al., 2005).

Membrane-bound receptors for IL-24 comprise IL-22R1, IL-20R1, and IL-20R2. IL-24/receptor interactions are mediated by the extracellular domains of these receptors. IL-24 signals through both the IL-20 receptor (IL-20R1/IL-20R2) and IL-20R1/IL-20R2 heterodimers (Dumoutier et al., 2001; Parrish-Novak et al., 2002; Wang et al., 2002). Upon binding and heterotrimer formation, IL-24 triggers a signaling cascade in the target cells through Janus Kinase/Signal Transducer and Activator of Transcription (JAK/STAT) pathway, which subsequently activates downstream transcription factors, like STAT3 and STAT1, through phosphorylation (Wang and Liang, 2005).

Since many physiological and pathophysiological roles of IL-24 critically depend on its interaction with cognate receptors (Wang et al., 2002; Wang et al., 2004), we hypothesize that controlling the binding between IL-24 and the shared receptor IL-20R2 could be useful for both basic research and therapeutics. The use of light to switch ON and OFF protein-protein interactions offers superior capabilities in terms of temporal and spatial resolution (Hoorens and Szymanski, 2018). We speculate that assays based on photocontrolled interleukins and/or their cognate receptors may be applied to the detection of specific interleukins, the screening of anti-interleukin/anti-receptor antibodies, or the screening of JAK/STAT inhibitors.

From the molecular engineering point of view, there are two major approaches to control protein functions by light (Kneuttinger, 2022). The first is hybrid protein optogenetics, where a chimera between an intrinsically light-responsive protein and a target protein is made. The second is photoxenoprotein engineering, where the target protein is modified at defined residues with light-responsive non-canonical amino acids (ncAA). NcAA can be incorporated into proteins by genetic code expansion technology through an orthogonal aminoacyl-tRNA synthetase (aaRS)/tRNA_CUA_ pair (Manandhar et al., 2021). Several photocontrolled ncAA have been genetically encoded in *Escherichia coli* and mammalian cells, including analogs of tyrosine, lysine, cysteine, serine and histidine (Wu et al., 2004; Deiters et al., 2006; Lemke et al., 2007; Gautier et al., 2010; Arbely et al., 2012; Nguyen et al., 2014; Hauf et al., 2017; Luo et al., 2017; Baumann et al., 2019; Cheung et al., 2023). We choose to photocage tyrosines in the form of *ortho*-nitrobenzyl-tyrosine (NBY) for two reasons. First, NBY has been proposed as a universal proximal cage for the temporal blockage of protein activity (Wang et al., 2019; Wang et al., 2021). Second, NBY has been successfully employed to photocontrol antigen-antibody interactions (Bridge et al., 2019; Jedlitzke et al., 2019; Joest et al., 2021; Jedlitzke and Mootz, 2022; O'Shea et al., 2022; Yilmaz et al., 2022; Bridge et al., 2023).

**FIGURE 1:**
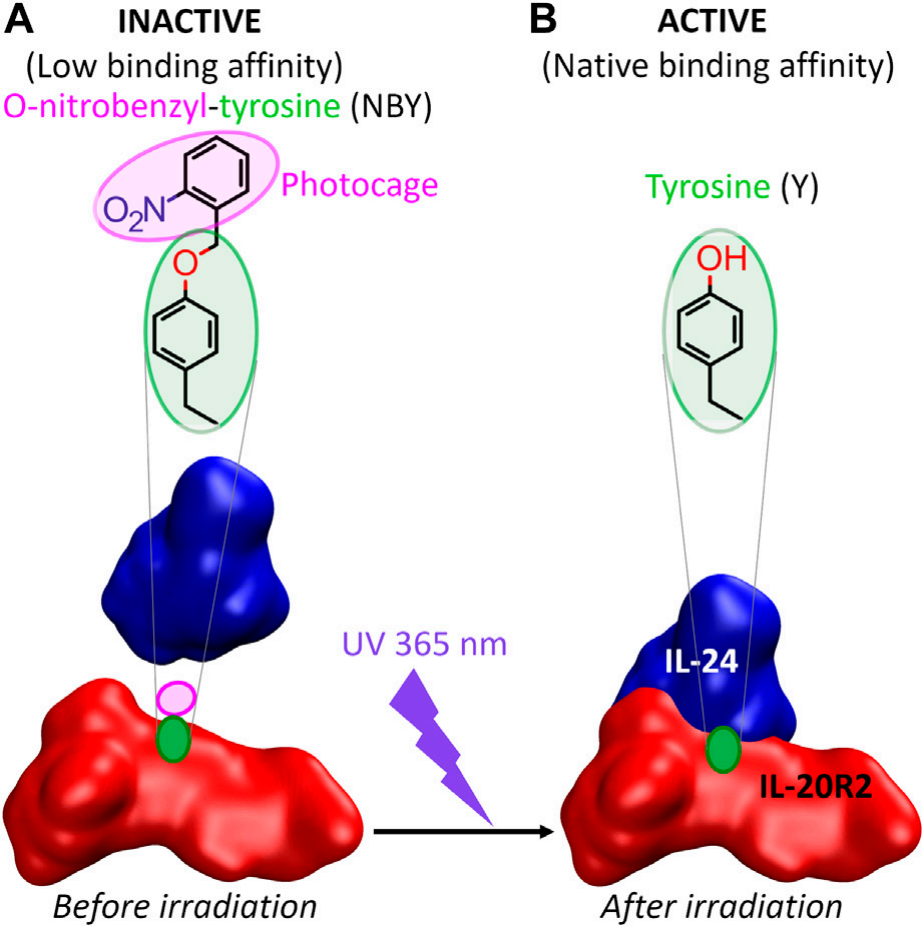
The concept of this study. **(A)** The association between IL-24 and IL-20R2 is diminished upon genetic encoding of a photocaged tyrosine (ortho-nitrobenzyl-tyrosine or NBY) into one of the two binding partners (IL-20R2 in the figure). The non-canonical amino acid must be introduced at a position, *a priori* unknown, which either blocks essential protein-protein interactions (e.g., hydrogen bonds) or causes steric clashes preventing a correct configuration of the interaction interface. **(B)** Irradiation with UV light (365 nm wavelength) releases the cage and reconstitutes a canonical tyrosine residue (and thus restores the native protein) that can now recognize the binding partner with the same affinity as the wild-type complex.

Thus, as a first step towards the photocontrol *in vivo* of cell signaling pathways that rely on IL-24-mediated complex formation, we designed and successfully tested the following *in vitro* approach with purified proteins recombinantly expressed in *E. coli* (Zahradník et al., 2019) (Figure 1). Incorporation of photocaged NBY residues at positions critical for molecular recognition into either IL-24 or IL-20R2 would keep the protein partners incompetent for binding and unable to convey the signal. Following the removal of the cage by UV irradiation, the native protein structure and activity (binding and signaling) would be restored resulting in complex formation. In the future, the ability to switch OFF the binding between IL-24 and IL-20R2 in the dark and switch it ON by UV light may find applications in immunology and possibly also in therapeutics.

## 2 Materials and methods

### 2.1 Miscellaneous chemicals

pEVOL-NBY was a gift from Dr. Petr Schultz (Deiters et al., 2006). NBY was purchased from Accela. For a more complete list of reagents, please refer to Supplementary Table S0.

### 2.2 Gene cloning

An engineered IL-24 variant named IL-24B4 had been sequence-optimized to achieve soluble expression in *E. coli* following structural bioinformatics design (Zahradník et al., 2019). This sequence contains 29 mutations relative to wild-type IL-24 (Supplementary Table S1, residue numbering according to UniprotKB Q13007). A pHisSumo vector containing such interleukin-24 variant with an N-terminal HisSumo tag (Zahradník et al., 2019) was used as the starting material for cloning. We used the primers 1 and 2 (Supplementary Table S2) to sandwich the IL-24 gene between an N-terminal HisSumo-tag and a C-terminal Strep-tag. The final construct was labeled as pHisSumo_IL-24B4_CStrep (Supplementary Figure S1A).

An IL-20R2 variant sequence, referred to as IL-20R2D, which was also optimized to be expressed and soluble in *E. coli*, contained 23 mutations relative to the wild-type (Supplementary Table S1 residue numbering according to UniprotKB Q6UXL0)*.* The design details can be found in the main text and Supplementary Note S1. The ordered DNA string consists of a HisSumo tag and a Strep tag flanking IL-20R2 at the N-terminus and C-terminus, respectively. The final construct was labeled as pHisSumo_IL-20R2D_CStrep (Supplementary Figure S1B).

TAG triplets to incorporate non-canonical amino acids were introduced in the plasmid sequences by standard site-directed mutagenesis using primers 5 to 11 (Supplementary Table S2).

### 2.3 Protein expression

To express proteins with canonical amino acids, the pHisSumo_IL-24B4_CStrep and pHisSumo_IL-20R2D_CStrep plasmids were transformed into *E. coli* BL21 AI strain. The bacterial cells were grown in TB medium containing 1.2% tryptone, 2.4% yeast extract, 0.5% glycerol, 33.7 mM Na_2_HPO_4_, 22.0 mM KH_2_PO_4_, 8.55 mM NaCl, 18.7 mM NH_4_Cl, 2 mM MgSO_4_/MgCl_2_, and metal mix. The antibiotic kanamycin was supplied to the medium and cells were grown at 30°C with shaking at 200 rpm. When bacterial OD600 reached 0.6 to 0.8, cells were induced with IPTG and arabinose at 0.1 mM and 0.02% v/v final concentration, respectively, and grown at 16°C for another 20 h.

For proteins containing non-canonical amino acids, plasmids encoding the proteins-of-interest were co-transformed in BL21 AI with pEVOL-NBY, a plasmid encoding a tyrosyl tRNA synthetase derived from *Methanocaldococcus jannaschii* specific for *ortho*-nitrobenzyl-tyrosine (*Mj*NBYRS) and its cognate suppressor tRNA (tRNA_CUA_) (Deiters et al., 2006).

Bacteria were grown at 30°C and shaking at 200 rpm in TB medium described above including antibiotics kanamycin and chloramphenicol. During culturing, we prepared a 50 mM stock of NBY by resuspending the amino acid in 50% DMSO heated at 70°C and adding NaOH dropwise until a homogeneous solution was obtained. When bacterial OD600 reached between 0.4 and 0.5, NBY was added to the culture at a final concentration of 1 mM. After 25 more minutes, the temperature was decreased to 16°C, and cells were induced by both IPTG and arabinose at 0.1 mM and 0.02% final concentration, respectively. Overnight-grown bacteria (20 h at 16°C) were separated from the medium culture by centrifugation at 5,000 *g* for 10 min at 4°C. All cell pellets were stored at −80°C until needed.

### 2.4 Protein purification

The cell pellet was suspended in cold washing buffer 50 mM Tris, pH = 8, 100 mM NaCl and sonicated 2 s on, 2 s off during 5 min with 50 W power on ice. The fraction of soluble proteins was collected by centrifuging at 40 000 g for 20 min at 4°C and the supernatant was passed over streptactin XT agarose beads equilibrated at room temperature by 50 mM Tris, pH = 8, 150 mM NaCl, 1 mM EDTA. After brief incubation for the proteins containing Strep tag to bind to the beads, the unbound proteins were washed with the same buffer. Commercial elution buffer BXT (100 mM Tris, pH 8.0, 150 mM NaCl, 1 mM EDTA, 50 mM biotin) was used to elute the bound Strep-tagged proteins. All the eluted proteins were further purified by size-exclusion chromatography (SEC) using a Superdex 75 Increase 10/300 GL column (GE Healthcare, Chicago, IL, United States) equilibrated with buffer 50 mM Tris, pH = 8, 100 mM NaCl.

IL-24B4 was digested with Sumo protease to remove the N-terminal HisSumo tag by at a ratio 1:200 (protease:protein) at room temperature overnight (16 h). The digestion mixture was passed over nickel-NTA agarose beads. Uncleaved IL-24 and the cleaved HisSumo fragment were retained on the beads, while cleaved IL-24 without the N-terminal HisSumo tag was recovered from the flow-through. Cleaved IL-24 was subsequently purified by SEC as described before, pooled and concentrated.

### 2.5 Protein characterization

The quality and quantity of all protein samples was checked by electrophoresis, UV/Visible spectroscopy, and mass spectrometry (de Marco et al., 2021). Proteins were analyzed by 15% sodium dodecyl sulphate–polyacrylamide gel electrophoresis (SDS-PAGE) and stained with Coomassie Blue. UV/Vis spectra were taken in a Nanodrop spectrophotometer (DeNovix) using 10 mm pathlegnth cuvettes. The protein extinction coefficient at 280 nm, estimated from the sequence using web server (https://web.expasy.org/protparam/) was used to calculate protein concentration based on Beer–Lambert law.

For mass spectrometry analyses, proteins were diluted with 100 µL of 5% acetic acid in water and loaded onto Opti-trap C4 cartridge (Optimize Technologies), washed 4 times with 250 µL of 5% acetic acid in water and eluted with 100 µL of 80% acetonitrile, 5% acetic acid. Proteins were analyzed by direct infusion using syringe pump at a flow rate 3 μL/min connected with an electrospray ion source of TimsTOF Pro mass spectrometer (Bruker Daltonics). The mass spectrometer was externally calibrated using 0.1% (w/w) sodium trifluoracetate. Proteins were measured in positive mode. The data were processed using SNAP algorithm–a part of DataAnalysis 5.3 software (Bruker Daltonics). Deconvoluted spectra were generated using the UniDec 5.0.2 software (University of Oxford) (Marty et al., 2015). IL-20R2D and IL-20R2D Y70NBY were also measured on a high resolving power and high accuracy 15T SolariX XR FT-ICR mass spectrometer (Bruker Daltonics), Expected protein masses (average and monoisotopic) were calculated with 2M data acquisition, by submitting the protein sequences to the website https://web.expasy.org/compute_pi/.

The circular dichroism spectra were measured using a 0.1 cm quartz cuvette on ChirascanTM-plus spectrometer (Applied Photophysics). Proteins were diluted in buffer (Tris 50 mM NaCl 100 mM pH = 8.0) to final concentration of ∼0.15 mg/mL. The data were recorded in the range of wavelengths of 190–260 nm with a step of 1 nm and time-per-point of 1 s at room temperature. The resulting spectra were water-subtracted and normalized to the concentration of the corresponding sample and are given as molar circular dichroism (Δε) vs wavelength. The data were processed by CDNN 2.1 software and BestSel (https://bestsel.elte.hu/index.php) (Micsonai et al., 2022).

Label-free protein unfolding assays were done by detecting the temperature-dependent change in tryptophan fluorescence at emission wavelengths of 330 and 350 nm (Alexander et al., 2014). Melting temperatures were determined from the maximum of the first derivative of the fluorescence ratios (F350/F330). For thermal unfolding experiments, the proteins were diluted to a final concentration of ∼0.15 mg/mL. The samples were loaded into UV capillaries (NanoTemper Technologies) and experiments were carried out using the Prometheus NT.48.

### 2.6 UV-decaging

200 μL of the proteins at 200 nM concentration were transferred to 1 mm path-length cylindrical cells (Hellma) and irradiated at 365 nm with an LED (Thorlabs M365L2) attached to a collimator (Thorlabs SM2F32-A) at 100 mW of power for 5 min at room temperature. Irradiated proteins were used immediately.

### 2.7 Affinity measurements by MST

We determine the binding affinity (dissociation constant) by MicroScale Thermophoresis (MST) using a Monolith NT.115 instrument (NanoTemper Technologies) (Jerabek-Willemsen et al., 2014). 200 nM of IL-20R2D, IL-20R2D Y70NBY, IL-20R2D Y74NBY, or IL-20R2D Y70NBY/Y74NBY were labeled with an NTA-conjugated fluorescent dye as recommended by the kit His-Tag Labeling RED-tris-NTA second generation. Binding assays were performed in buffer 50 mM Tris, pH = 8, 100 mM NaCl, 0.02% pluronic F127. The instrument settings were 20% MST power and 40% LED power. Data from triplicate experiments were fitted to mass-action kinetics by using the NT Analysis software version 1.5.41:

$$F\left(c\right)=A+B\left(\frac{c+c_{T}+K_{d}-\sqrt{{\left(c+c_{T}+K_{d}\right)}^{2}-4\;c\;c_{T}}}{2\;c_{T}}\right)$$ (1)

Where *F(c)* is the fluorescence signal as a function of concentration; *c* is the concentration of IL-24B4 (unlabeled) *c*_*T*_ is the concentration of IL-20R2D variants (fluorescently labeled), which was held constant at 50 nM; and *K*_*d*_ is the dissociation constant; A is the titration curve baseline; B is the titration response range. To facilitate the comparison between datasets with distinct values of A and B, the fraction of bound molecules (normalized between zero and one) was calculated as:

$$Fraction\;bound=\frac{F\left(c\right)-A}{B}$$ (2)

### 2.8 Affinity measurements by yeast display

Yeast surface display of IL-24B4 and subsequent binding experiments were performed by using the enhanced yeast display platform pJYDC1 plasmid (Addgene ID: 162458) and *Saccharomyces cerevisiae* EBY100 strain (Zahradník et al., 2021a). The bait, an IL-24B4 version including an N-terminal linker of 17 amino acids, was cloned between *Nde*I and *Bam*HI sites (see full construct sequence in Supplementary Table S1), and transformed into yeast by lithium acetate method. Protein expression was achieved by cultivation in galactose-rich expression media 1/9 at 20°C for 24 h (Zahradník et al., 2021a). Prey proteins (IL-20R2D, IL-20R2D Y70NBY and their UV irradiated versions) were labeled by using CF^®^640R succinimidyl ester dye (Biotium) in 1:3 M excess.

The binding affinities of labeled IL-20R2D versions to IL-24B4 expressed on yeast surface were determined by flow cytometry titration experiments. We used 5 different concentrations (500, 100, 20, 4, and 0.1 nM) of labeled prey protein in PBS buffer supplemented by 1 g/L of Bovine Serum Albumin Fraction V (PBSB buffer, Sigma-Aldrich, Cat#10735078001), and 10 nM bilirubin (Sigma-Aldrich, Cat# 14370) for activation of eUnaG2 fluorescence (excitation at 498 nm, emission at 527 nm). To increase the control over the measurement, we enriched the suspension by addition of 0.5% of IL-24S, an engineered clone featuring 300 times tighter binding, which was obtained by yeast display affinity maturation (Supplementary Table S1 and Supplementary Figure S2).

Binding suspensions were incubated overnight at 4°C, washed twice with ice-cold PBSB and analyzed by using CytoFLEX S Flow Cytometer (Beckman Coulter, United States, Cat#. N0-V4-B2-Y4). The gating and analysis strategies were described previously (Zahradník et al., 2021a; Zahradník et al., 2021b). Briefly, the unspecific binding signals were subtracted from the binding signals and a Eq. 1 was fitted to the data by nonlinear least-squares regression using Python v3.7 (https://www.python.org).

### 2.9 JAK/STAT signaling assays in human cells

HeLa cells were gown in a media containing Dulbecco’s modified Eagle’s medium supplemented with 10% fetal bovine serum, 1% of PenStrep and 1% of sodium pyruvate. To ensure sufficient amounts of the receptors IL-20R2 and IL-22R1, two plasmids encoding the native sequences (Supplementary Table S1) were constructed (oligos 12 to 15 of Supplementary Table S2) and co-transfected in HeLa cells (0.66 μg of IL-20R2 and 1.33 μg of IL-22R1) using the jetPrime transfection reagent (Polyplus 114–07) following manufacturer’s instructions. The next day, the cells were treated for 30 min with the obtained interleukin variants at the indicated concentrations. After the incubation period, the plates were put on ice, the media discarded, and cells were washed with cold PBS buffer. Then, 100 μL of RIPA lysis buffer (Tris 50 mM pH = 8.0, NaCl 150 mM, EDTA 5 mM, IGEPAL CA-630 1%, sodium deoxycolate 10%, SDS 10%) supplemented with protease and phosphatase inhibitors was added to each well. Cells were scraped and put into Eppendorf tubes for 20 min on ice, followed by centrifugation at 4°C, 18,000 g for 30 min. Protein concentration in the supernatant fraction was quantified by the BCA assay (QPRO BCA kit from Cyanagen) using BSA as the standard. Equal protein amounts were loaded on SDS-PAGE gels. Western blotting to nitrocellulose membranes was done in a Transblot Turbo Bio-rad system. For immunoblotting we use two primary antibodies: anti-phospho-STAT3 (Tyr705, D3A7) monoclonal antibody raised in rabbit, and anti *a*-tubulin monoclonal antibody raised in mouse (Sigma). As secondary antibodies we used anti-mouse or anti-rabbit peroxidase-conjugated IgG. One milliliter of peroxidase substrate was added to the membranes and the resulting chemiluminiscence was imaged using an Azure400 system. All original Western blot images can be found in the Supplementary Zip file. Band quantification was done in ImageJ (https://imagej.nih.gov/ij/index.html) as previously described (Stael et al., 2022). The band intensities of pSTAT3 were divided by the corresponding band intensities of tubulin and subtracted from a control experiment (untreated cells). Normalized and background-subtracted band intensities were plotted as a function of the concentration of added variant and a dose-response curve was fitted to the data:

$$\frac{pSTAT3}{tubulin}=\frac{Y_{\max}}{1+{\left({EC}_{50}/c\right)}^{n_{H}}}$$ (3)

Where *Y*_max_ is the maximum observed signal, *EC*_*50*_ is the half maximal effective concentration, *c* is the concentration of added interleukin (IL-24B4, IL-20R2D and their NBY counterparts), and *n*_*H*_ is the Hill coefficient (which was fixed to 1, i.e., non-cooperative binding, to prevent overfitting due to the reduced number of points).

## 3 Results

### 3.1 Protein design combining canonical and non-canonical amino acid mutagenesis

*In vitro* testing of photo-induced changes in binding affinity requires pure and active protein partners. We designed engineered variants that are more amenable to downstream manipulations and may be used as surrogates of wild-type proteins after a scrutiny of their performance.

#### 3.1.1 Enhancing solubility and stability with canonical amino acids

In our previous work, we successfully optimized the sequence of IL-24 based on canonical amino acid mutagenesis to achieve soluble expression in *E. coli* hosts (Zahradník et al., 2019). Here we have applied a similar approach to the high affinity receptor of IL-24, the IL-20R2. In order to obtain an stabilized soluble extracellular portion of IL-20R2 protein, we employed PROSS algorithm (Goldenzweig et al., 2016) (Supplementary Note S1, Supplementary Table S1, Supplementary Figure S2, and Supplementary Figure S3A). We then tested the expression of our newly designed IL-20R2D in *E. coli* BL21 (DE3) under the control of T7 promoter and compared it to IL-20R2 wild-type under the same conditions. Unlike the wild-type gene, our construct is expressed as a soluble protein in large quantities ∼20 mg.L^-1^ (Supplementary Figure S3B). Subsequent CD and melting measurements showed evidence of a folded protein (Supplementary Figure S3C) with a melting temperature of ∼45°C (Supplementary Figure S3D). The protein showed considerable stability and retention of binding to IL-24B4 (Zahradník et al., 2019).

#### 3.1.2 Adding photocontrol with photocaged ncAA

**FIGURE 2:**
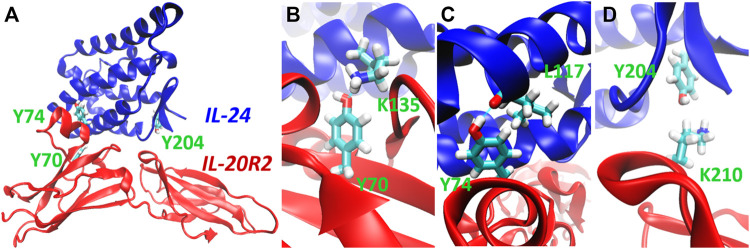
Selection of target residues for substitution with photocaged counterparts. **(A)** 3D model of the complex between IL-24 (blue) and IL-20R2 (red) based on X-ray crystallography (PDB 6DF3). The three chosen tyrosines (tyrosine70, tyrosine74, and tyrosine204) are highlighted in green. **(B)** Close-up view of the H-bond between tyrosine70 (IL-20R2) and lysine135 (IL-24). **(C)** Close-up view of the H-bond between tyrosine74 (IL-20R2) and leucine117 (IL-24). **(D)** Close-up view of Van der Waals contact between lysine210 (IL-20R2) and tyrosine204 (IL-24).

As mentioned before, we chose to photocage tyrosine residues with the o-nitrobenzyl moiety. We then looked for tyrosine residues at the binding interface between IL-24B4 and IL-20R2D. Since no high-resolution structure exists of the complex between the variants used in the present study, we used the X-ray crystal structure of the homologous native ternary complex (IL-24/IL-22R1/IL-20R2, PDB ID: 6DF3) (Lubkowski et al., 2018) (Figure 2A). The structure suggests that the main contribution to complex formation are the contacts between IL-24 and IL-20R2. The IL-24/IL-20R2 interface is shown schematically in 2D (Supplementary Figure S4A). The interface area covers ∼900 Å^2^ and is stabilized by several forces including 13 hydrogen bonds (H-bonds), 2 potential salt bridges, and a number of polar, water-mediated and hydrophobic contacts. We identified three tyrosine candidates: one in IL-24 (Y204), and two in IL-20R2 (Y70 and Y74). The phenolic hydroxyl group of tyr70 in IL-20R2 makes a hydrogen bond (H-bond) with the ε-amino group of lys135 in IL-24 (Figure 2B). The sidechain OH of tyr74 in IL-20R2 is H-bonded to the backbone CO group of leu117 in IL-24 (Figure 2C). Finally, tyr204 in IL-24 makes Van der Waals interactions with lys210 of IL-20R2 (Figure 2D) and is also involved in H-bond interactions with IL-22R1 (Supplementary Figures S4B–E). Considering all these data, we prepared three single mutants: IL-24B4 Y204NBY, IL-20R2D Y70NBY and IL-20R2D Y74NBY. In addition, we prepared the double mutant of IL-20R2D Y70NBY/Y74NBY.

### 3.2 Production and characterization of photocaged interleukins and receptors

**FIGURE 3:**
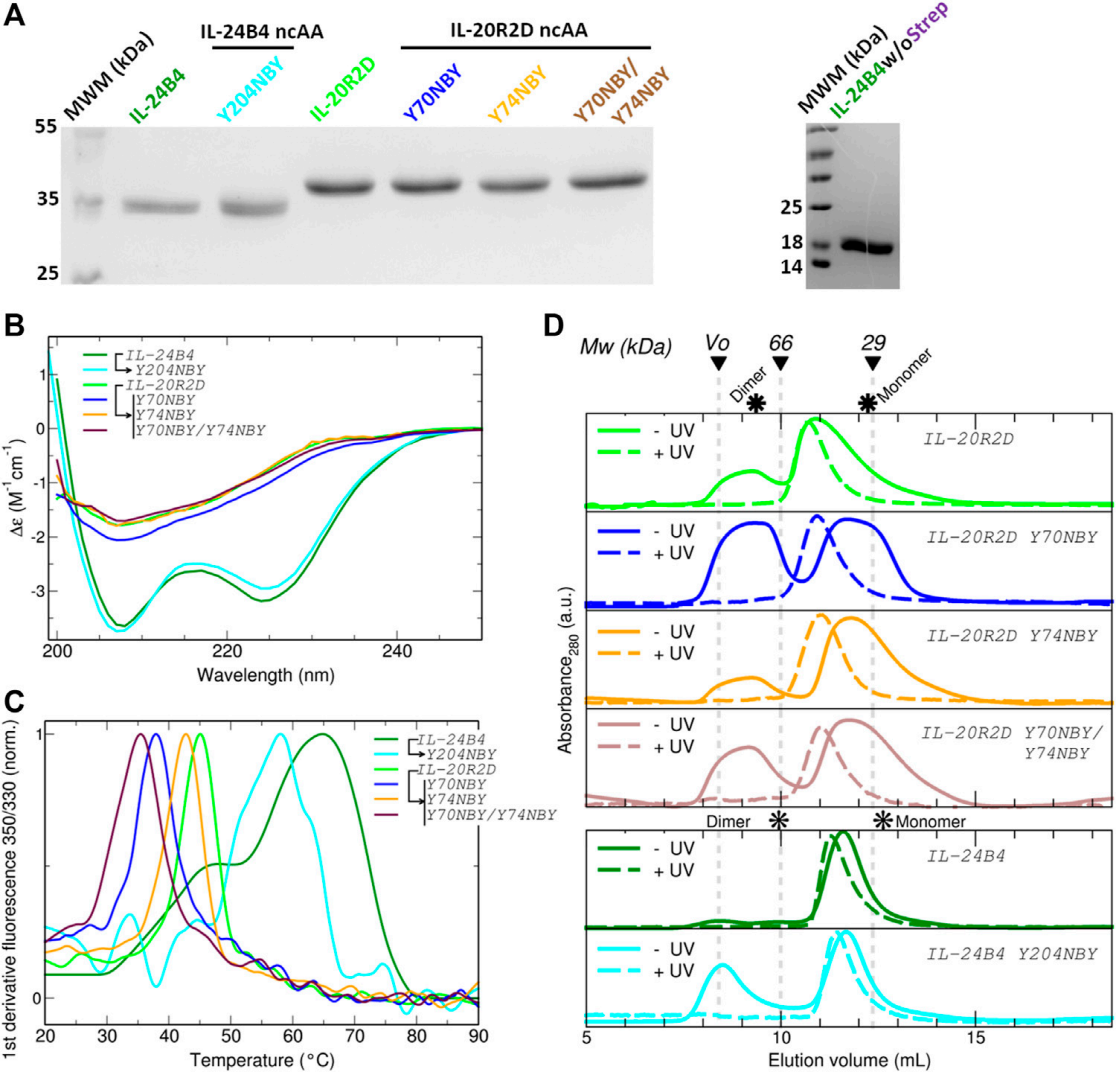
Expression, purification and characterization of parental and caged interleukins. **(A)** SDS-PAGE of all purified variants fused to SUMO (*left* panel) and SUMO-cleaved IL-24B4 (*right* panel). **(B)** Circular dichroism spectra expressed as per residue molar absorption (Δε). **(C)** Thermal denaturation curves expressed as the normalized first derivative of the fluorescence emission ratio between 350 nm and 330 nm. **(D)** Size-exclusion chromatograms of all studied variants before (solid lines) and after (dashed lines) UV irradiation. The molecular weight of gel filtration markers is shown on top. The asterisks indicate the expected elution volumes for both monomeric and dimeric assemblies. Data in panels **(B)**, **(C)** and **(D)** were taken in Tris 50 mM Nacl 100 mM pH = 8.

For the design of the final constructs, we considered one additional phenomenon. Codon decoding by orthogonal aminoacyl-tRNA synthetase/tRNA_CUA_ pairs is not 100% efficient, i.e., protein translation can also terminate at the introduced codon resulting in a mixture of truncated and full-length proteins that can be difficult to separate. To avoid such a problem, we added a C-terminal Strep-tag. All our final constructs express target proteins containing a HisSumo tag at the N-terminus and a Strep-tag at the C-terminus. First, we checked whether addition of the Strep tag alters the yield of IL-24B4 and IL-20R2D. The purity was assessed by SDS-PAGE (Figure 3A). In the case of IL-24B4, the HisSumo tag was removed to avoid interference with downstream affinity measurements by microscale thermophoresis (MST). This is because for MST, the His-tagged target protein is non-covalently labeled with a NTA-conjugated fluorescent dye. Mixing with another protein containing a His-tag will cause the dye to re-equilibrate between the two binding partners thus confounding the experiment. Sumo-free IL-24B4 is shown in Figure 3A (right panel).

To obtain proteins with NBY incorporated at specific positions, an evolved orthogonal aminoacyl tRNA-synthetase (*Mj*NBYRS) and suppressor tRNA (tRNA_CUA_) pair was added to the native bacterial translation system (Deiters et al., 2006). Moreover, the bacteria cells were fed with the dissolved non-canonical amino acid NBY in TB culture medium. The expressed full-length non-canonical receptors were purified by one-step Strep-tag procedure as done for the parental proteins. In addition, the negative controls without NBY supplement were performed for size comparison. Clear bands at the expected molecular weight were found in the case IL-24B4 Y204NBY (Supplementary Figure S5A) IL-20R2D Y70NBY, IL-20R2D Y74NBY (Supplementary Figure S5B) in the presence of ncAA. On the contrary, for the samples without added NBY there were essentially no proteins (Supplementary Figure S5). These results suggest that the ncAA NBY was incorporated into the interleukins and their receptors at selected residues only in *E. coli* cells containing both the non-canonical amino acid and the orthogonal translation machinery.

**FIGURE 4:**
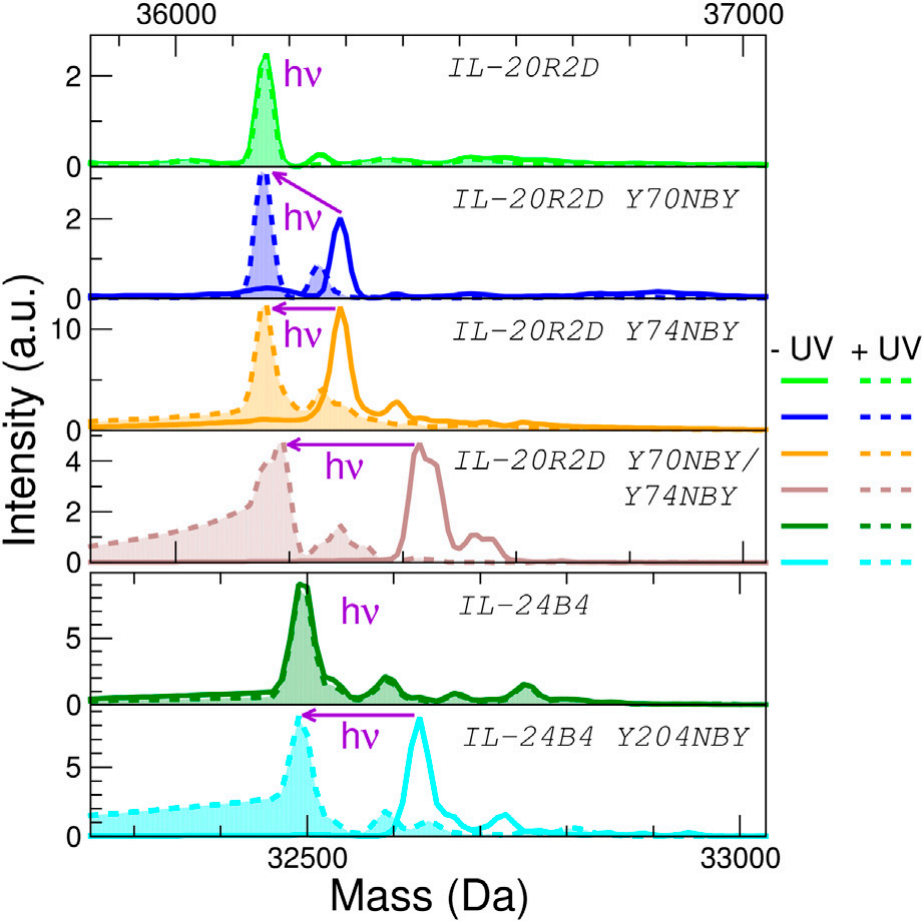
Analysis of the effect of protein UV irradiation by mass spectrometry. Deconvoluted mass spectra of the six interleukin variants used in this study before (solid lines) and after (dashed lines with filled areas) exposure to light (λ = 365 nm, 5 min at 100 mW) No significant mass changes are observed for the parental proteins (IL-20R2D and IL-24B4) upon UV irradiation. NBY-containing IL-20R2D and IL-24B4 are converted back to IL-20R2D and IL-24B4, respectively, by UV light. Average masses can be found in Supplementary Table S3.

To check whether NBY was indeed present in the purified polypeptides, mass spectrometry was employed (Figure 4). The experimentally determined masses are very close to the theoretical masses (plus/minus 2 Da) (Supplementary Table S3). We did not detect mis-incorporation of phenylalanine residues or reduction of the nitro group, two common issues associated to the genetic encoding of NBY (Koch et al., 2022). These results confirm unambiguously that the non-canonical amino acid NBY was introduced into the target proteins at high levels.

We further characterized the obtained protein variants by circular dichroism (CD) and differential scanning fluorimetry (DSF). The CD spectra of the IL-24B4 variants showed two minima at ∼208 nm and ∼224 nm (Figure 3B). In opposition, the CD spectra of the IL-20R2D variants featured a single minimum around 207 nm (Figure 3B). Analysis of secondary structure content revealed that IL-24B4 variants were enriched in α-helices while IL-20R2D mutants contained predominantly antiparallel *ß*-sheets (Supplementary Table S4) in agreement with their crystal structures. Analysis of melting temperatures by DSF indicated significant differences in thermal stability among the mutants (Figure 3C). IL-24 variants were more stable than IL-20R2D variants, ∼60°C vs. ∼40°C, respectively (Supplementary Table S5). In both cases, NBY reduced the protein’s melting temperature. The largest effect was observed in the double mutant (Y70NBY/Y74NBY) that had ∼10°C lower melting temperature than the parental protein. Therefore, although the introduced NBY caused minimal structural changes, protein stability was reduced.

### 3.3 Testing the effect of UV irradiation

Supplementary Table S6 shows the employed UV wavelengths together with protein’s UV/Visible absorption spectra. Prior to measuring protein affinities and cellular signaling, we checked the effect of UV light on protein oligomerization and molecular weight by size-exclusion chromatography (SEC) and mass spectrometry. According to SEC (see chromatograms in Figure 3D), the apparent molecular weights of all studied variants lay in between the expectation for a monomer and a dimer (Supplementary Table S6). Therefore, at least two interpretations are possible. Either the proteins are in monomer/dimer equilibrium or they feature an expanded (“open”) monomeric conformation The NBY mutations tend to make the proteins more aggregation prone and substantial protein amounts were found in the void volume. Importantly, no major changes in the elution profiles were observed before/after UV and no large aggregates were found upon UV exposure. However, UV-irradiated samples eluted earlier than the non-irradiated counterparts (Supplementary Table S6). Although partial UV-induced dimerization cannot be completely ignored, we conclude that a molecular interaction with a 1:1 stoichiometry is a suitable analytical model.

Second, we ruled out any potential UV-induced photodamage to the proteins. The masses of IL-24B4 and IL-20R2D before and after illumination were virtually identical, suggesting that the irradiances used in this study are insufficient to cause significant residue modifications (Figure 4). Next we checked the efficiency of decaging. We shined UV light on the 4 NBY-containing variants and observed that the mass decreased ∼135 Da (or ∼270 Da in the case of the double mutant), which corresponds to that of the nitrobenzyl moiety, making it indistinguishable from the mass of canonical variants (Figure 4). For better visualization of the mass difference we also show detailed mass spectra of 35+ charge state (Supplementary Figure S7).

Thus, we prove that all photocaged interleukins/receptors can be efficiently decaged by UV irradiation, thereby regenerating the parental proteins natively containing a tyrosine residue (or residues in the case of the double mutant).

### 3.4 Measuring interleukin-receptor interactions and their effects

Having found conditions for efficient decaging, we next monitored the effect of the produced interleukin variants on IL-24/IL-20R2 complex formation in three environments of increasing complexity (Supplementary Figure S8). First, with both interacting partners free in solution by MST. Second, with one binding partner immobilized on the yeast cell surface and the other in solution by yeast display. Finally, with the receptors natively inserted in the membrane of human cells and the others exogenously added to the growth media. The first two methods directly report on IL-24/IL-20R2 binding affinities, while the latter is sensitive to the impact that such interactions have on signal transduction through the JAK/STAT pathway.

#### 3.4.1 MST measurements

**FIGURE 5:**
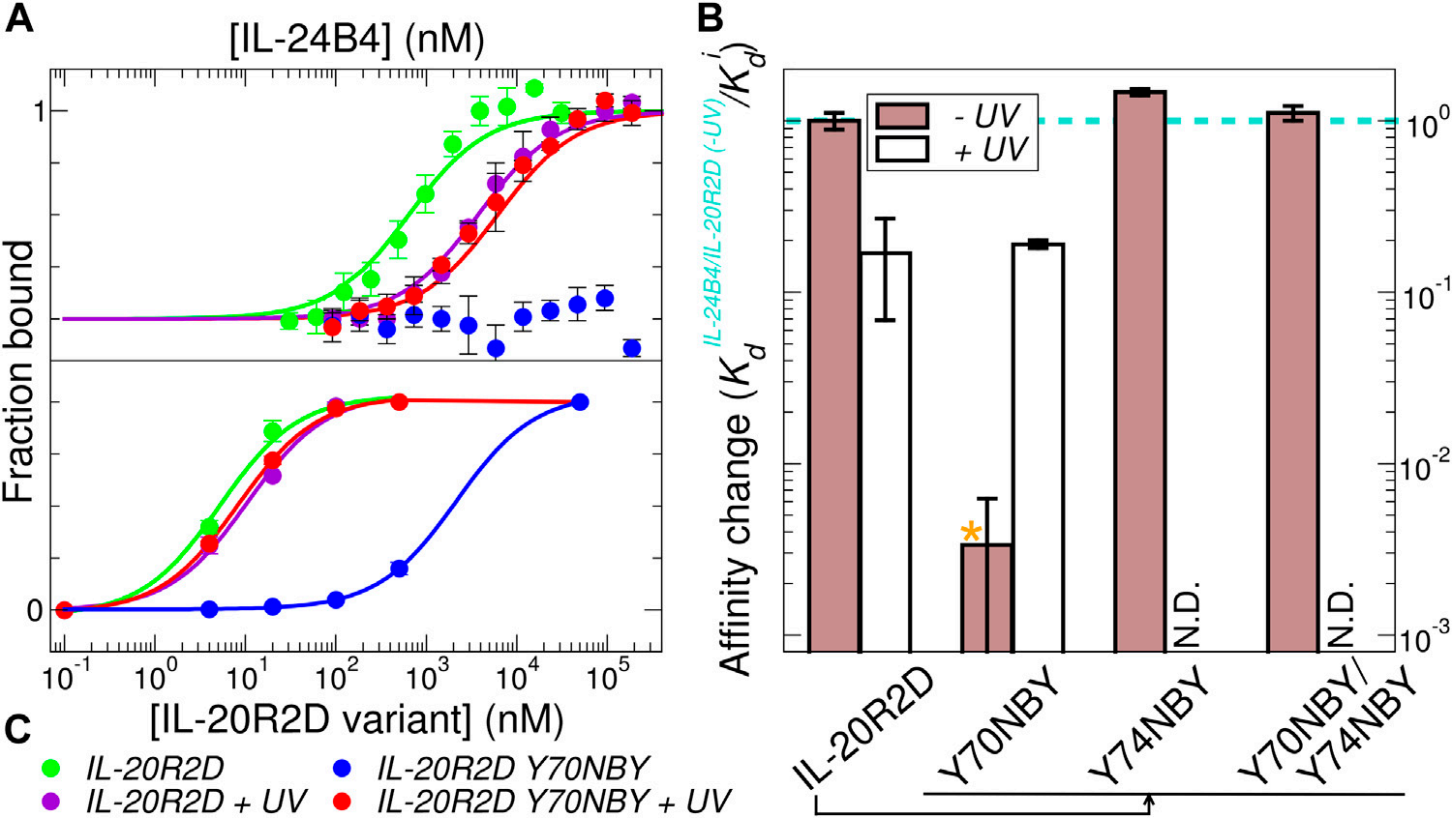
Estimation of binding affinities by microscale thermophoresis and yeast surface display. **(A)** Fraction of bound molecules as a function of IL-24B4 concentration according to MST experiments. **(B)** Fold-change in binding affinity relative to IL-24B4/IL-20R2D without UV irradiation of the four studied variants (IL-20R2D, IL-20R2D Y70NBY, IL-20R2D Y74NBY, and IL-20R2D Y70NBY/Y74NBY) by MST. The bars represent the average and standard deviation of 3 independent experiments. No significant binding was detected for the mixtures between IL-20R2D Y70NBY and IL-24B4 within the concentration range used. Therefore, the reported *apparent K*_*d*_ (orange asterisk) should be considered as lower boundary. **(C)** Fraction of bound molecules as a function of IL-20R2D concentration according to yeast display experiments. Solid lines in panels **(A)** and **(C)** are fits to Eq. 1 in order to retrieve the dissociation constants (see *K*_*d*_ values in Table 1).

We determined binding affinities by MST, which measures temperature-induced changes in fluorescence intensity, using fluorescently labeled variants at a fixed concentration and mixing with variable amounts of unlabeled binding partners. Unfortunately, the obtained amounts of IL-24B4 204NBY were too low. Hence, we concentrated on NBY-containing mutants of IL-20R2D for which we obtained sufficient yields for biophysical studies. The dissociation constant (*K*_*d*_) of the complex between IL-24B4 and IL-20R2D is ∼0.5 μM (see *K*_*d*_ values in Table 1 and raw data in Supplementary Figure S9) in agreement with the literature (Zahradník et al., 2019). Exposure to UV light slightly diminished the binding affinity by a factor of 6 (compare green and violet traces in Figure 5A). Of the three tested variants containing photocaged non-canonical amino acids, IL-20R2D Y74NBY and the double-mutant IL-20R2D Y70NBY/Y74NBY bound to IL-24B4 with similar affinities as their canonical counterparts (Figure 5B). Non-irradiated IL-20R2D Y70NBY showed virtually no binding to IL-24B4 within the concentration range used (Figure 5A), suggesting a decrease of binding affinity by a factor of at least 300 relative to the canonical complex (Figure 5B). Gratifyingly, photoconversion of IL-20R2D Y70NBY back to IL-20R2D by UV light shifted the binding curve towards the values observed for the canonical protein (Figure 5A). Thus, we found one residue position in IL-20R2D, Y70, where NBY significantly blocks the heterodimerization with IL-24B4. In such a case, complex formation can be observed after cage photolysis by UV light. Unexpectedly, the double-mutant displayed high binding affinity in the absence of UV irradiation suggesting non-additive effects of the two mutations where the inhibitory effect of Y70NBY is somehow compensated for by Y74NBY.

**TABLE 1:** Binding affinities between IL-24B4 and IL-20R2D variants. Dissociation constant (*K*_*d*_) and half-maximal effective concentration (*EC*_50_) values are expressed as the mean ± s.d. (3 biological replicates for MST, 3 technical replicates for yeast display, and 2 technical replicates for Western blot).

	*Method*	*MST*	*Yeast display*	*Cell signaling (pSTAT*3*)*
Partner 1^a^	Partner 2	*K*_*d*_ (nM)	*K*_*d*_ ^b^ (nM)	*EC*_50_ ^c^ (nM)
*IL-*24*B*4	*IL-*20*R*2*D* ^c^	630 ± 140	5.0 ± 0.6	0.28 ± 0.07
*IL-*24*B*4 *Y*204*NBY*	*IL-*20*R*2*D* ^c^	N.P.	N.D.	0.9 ± 0.5
*IL-*24*B*4 *Y*204*NBY + UV*	*IL-*20*R*2*D* ^c^	N.P.	N.D.	0.76 ± 0.24
*IL-*24*B*4	*IL-*20*R*2*D + UV*	3,700 ± 400	9.6 ± 1.6	N.P.
*IL-*24*B*4	*IL-*20*R*2*D Y*70*NBY*	>94,000	∼2000	N.P.
*IL-*24*B*4	*IL-*20*R*2*D Y*70*NBY + UV*	3,300 ± 600	7.6 ± 0.4	N.P.
*IL-*24*B*4	*IL-*20*R*2*D Y*74*NBY*	430 ± 30	N.D.	N.P.
*IL-*24*B*4	*IL-*20*R*2*D Y*70*NBY/Y*74*NBY*	570 ± 60	N.D	N.P.

^a^ For microscale thermophoresis (MST) experiments the partner 2 was held constant and partner 1 titrated, while the opposite is true for yeast display experiments.

^b^ *K*_*d,*_ values obtained from yeast display should be considered *apparent* because they reflect not only affinity but also avidity due to the high protein densities on the cell surface.

^c^ Production of phosphorylated STAT3 (pSTAT3) as a function of added partner 1. Note that in this case the partner 2 corresponds to IL-20R2/IL-22R1, expressed in HeLa cells.

N.D, means not determined; N.P, indicates a measurements that it is not possible due to low sample amounts or limitations of the cell assays (see main text more details).

#### 3.4.2 Yeast display measurements

To support the previous results in a more cellular context, we also estimated binding affinities using yeast display. The four purified and fluorescently labeled IL-20R2D variants (canonical and non-canonical, with and without UV stimulation) were incubated at different concentrations with yeast cells expressing on their surface IL-24B4 fused to a fluorescent protein. The fluorescent signal arising from each binding partner was then analyzed by flow cytometry (Supplementary Figure S10). In these plots, binding events appear along the diagonal. UV illumination did not cause a significant change (2-fold reduction) in the population of IL-20R2D/IL-24B4 complex (Figure 5C). In the case of IL-20R2D Y70NBY in the absence of UV light, complex formation with IL-24B4 was largely attenuated within the concentration range used (Figure 5C). Upon illumination, the number of heterodimers increased and reached similar levels as for the canonical complex (Figure 5C). The apparent *K*_*d*_ values, including both affinity and avidities, are listed in Table 1.

When comparing the relative trends, both MST and yeast display agree in suggesting low levels of complex formation between IL-20R2D Y70NBY and IL-24B4 in the absence of light. However, the binding affinities estimated by yeast display were higher (approximately 100-fold) than those determined by MST in diluted protein solutions, probably due to avidity (multiple interaction events) and crowding effects. In summary, the two methods point to two completely different binding scenarios. On one hand, low binding affinity of IL-20R2D Y70NBY (caged receptor) against IL-24B4 in the dark. On the other hand, high binding affinity (similar to the parental proteins) upon UV illumination and subsequent decaging.

#### 3.4.3 Cell signaling assays

Eventually, we measured the ability of our engineered variants IL-20R2D and IL-24B4 to activate the JAK/STAT signaling cascade in a human cell line (HeLa). To this end, we quantified the levels of the transcription factor STAT3 phosphorylated at tyrosine705 (pSTAT3 for short), which is a well-known marker implicated in the signaling of IL-24 and other cytokines (Darnell et al., 1994). pSTAT3 levels after 30 min exposure with canonical or non-canonical interleukin variants were determined by Western blotting using an anti-pSTAT3 antibody and subsequent enhanced chemiluminiscence detection.

Initially, we checked whether our engineered versions (IL-24B4 and IL-20R2D) and the endogenous levels of native receptors (IL-20R2 and IL-22R1) present in HeLa cells can yield detectable amounts of pSTAT3. As a positive control we used interferon-α (IFNα), a cytokine that is known to induce rapid changes in JAK/STAT phosphorylation and initiate the signaling (Pellegrini et al., 1989). Little production of pSTAT3 was found in non-transfected cells regardless of the presence of IL-24B4 (Supplementary Figure S11A). On the contrary, significant amounts of pSTAT3 were found in transfected cells upon addition of IL-24B4 (Supplementary Figure S11A) suggesting that HeLa cells express limited amounts of endogenous receptors and require a boost of IL-24 receptors to enhance signal-to-noise. Similar amounts of pSTAT3 were found in the case of interferon-α stimulation (Supplementary Figure S11B). To test the capability of IL-20R2D to transduce biological signals, we created a chimeric receptor (IL-20R2DC) where the extracellular portion of full-length IL-20R2 was replaced by the designed IL-20R2D (Supplementary Table S1). The results (Supplementary Figure S11C) support the notion that IL-20R2DC can bind IL-24B4 and trigger signaling.

Surprisingly, we found that IL-20R2D signals through the same receptors and pathway although at high protein concentrations (Supplementary Figure S12A). Signal transduction through IL-20R2 was previously observed for IFNαR2 (Pattyn et al., 1999). Such a background level of pSTAT3 induction by IL-20R2D would complicate the interpretation of the results when both IL-24B4 and IL-20R2D are present. Taken together, these results confirm that the designed variants, IL-24B4 and IL-20R2D, recapitulate the role of native proteins, i.e., IL-24B4 binds its cognate membrane-bound receptors (IL-20R2, IL-22R1) and initiates the JAK/STAT pathway. However, a limitation of our cell signaling assays is that they do not unambiguously inform on the effect of IL-20R2D.

**FIGURE 6:**
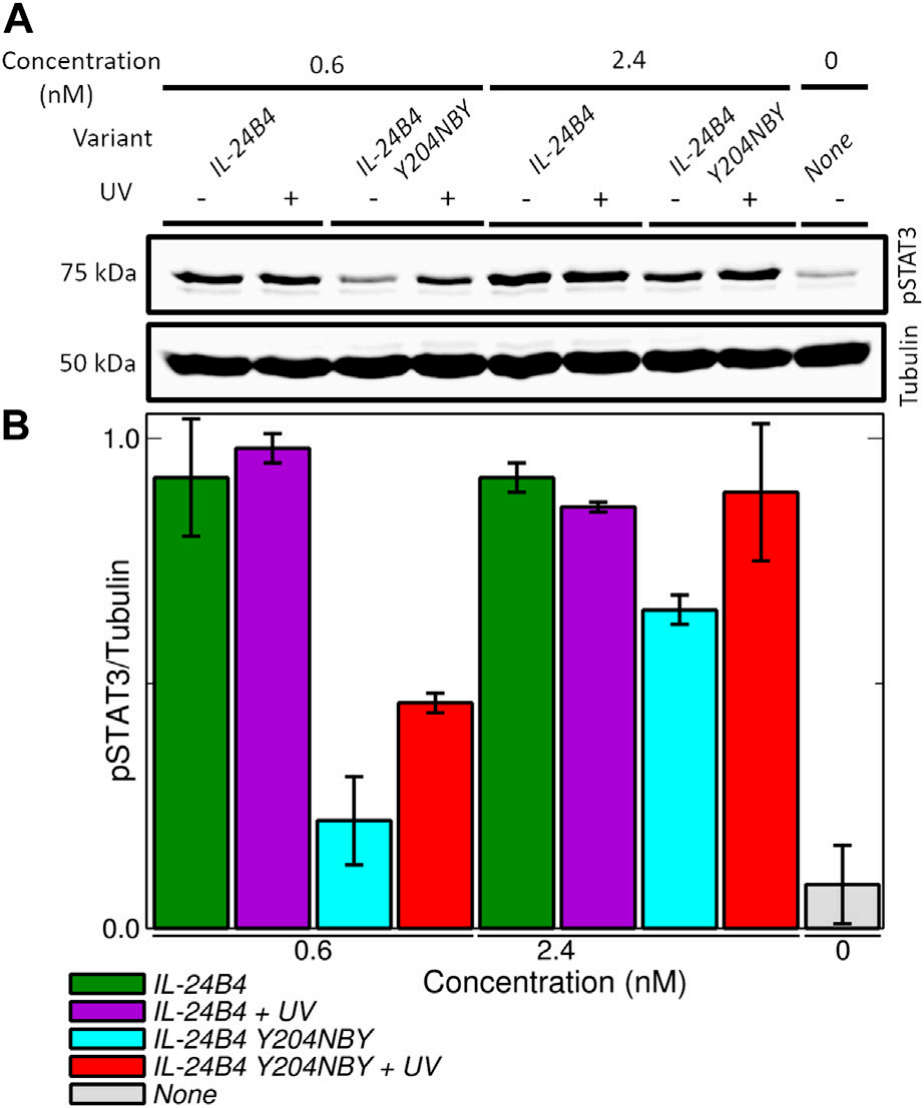
Signaling assays in HeLa cells transfected with plasmids encoding IL-20R2/IL-22R1. **(A)** Western blots showing the production of phosphorylated STAT3 (pSTAT3) upon induction of JAK/STAT signaling pathway by exogenously added amounts of IL-24B4 and its photocaged variant Y204NBY both in the presence and absence of UV irradiation (λ = 365 nm, 5 min at 100 mW). **(B)** Quantification of pSTAT3 (normalized by tubulin) as a function of the added variants and concentrations. The bars represent the average and standard deviation of 2 independent experiments.

Therefore, we focused on the IL-24B4 Y204NBY mutant, which could be purified in sufficient amounts for the cell assays. We measured the intracellular levels of pSTAT3 reporter after incubation with different concentrations of IL-24B4 and IL-24B4 Y204NBY without and with UV irradiation (Supplementary Figure S12B). The more IL-24B4 was added to the cells the more pSTAT3 was obtained until saturating levels were reached. A non-cooperative Hill model was fitted to the dose-response curves (Supplementary Figure S12C) and the resulting half-maximal effective concentration (*EC*_*50*_) values are reported in Table 1. The *EC*_*50*_ of caged IL-24B4 Y204NBY was ∼3 times larger than that of parental IL-24B4 but similar to the decaged counterpart considering experimental uncertainties. At concentrations higher than *EC*_*50*_, all four IL-24B4 variants behaved similarly as expected (Figure 6). Only at concentrations near *EC*_*50*_, the caged Y204NBY variant signaled less than the parental protein, and UV irradiation increased pSTAT3 levels but without reaching the amounts observed for the parental protein (Figure 6). We conclude that IL-24B4 Y204NBY is another promising candidate for future studies.

## 4 Discussion

Since the interaction between human interleukin-24 and its receptor IL-20R2 triggers the JAK/STAT signaling pathway, and acts on angiogenesis and cell proliferation, we are interested in controlling this interleukin-receptor system. In this work, we have genetically encoded in each binding partner *ortho*-nitrobenzyl-tyrosine, an unnatural photocaged analogue of tyrosine, to photoregulate IL-24/IL-20R2 interactions. Our main finding is that IL-20R2D Y70NBY and, to a lesser extent, IL-24B4 Y204 hamper complex formation and subsequent activation of the STAT3 cascade. Mild UV irradiation partially restores heteroassociation and cell signaling.

### 4.1 Photocontrolling protein-protein interactions involving interleukins with ncAA

Protein-protein interactions (PPI) lie at the heart of many biological processes ranging from metabolic pathways to signaling cascades. Moreover, abnormal PPI are associated with various diseases, including cancer (Lu et al., 2020). Therefore, the modulation of PPI is important not only for basic research but also for the development of new drugs. Research related to the control of PPI has focused primarily on antigen-antibody interactions (Lucchi et al., 2021).

Among several approaches to photocontrol PPI (Carrasco-López et al., 2020; Gil et al., 2020), genetically encoded photo-responsive non-canonical amino acids offer several advantages: i) a precise level of temporal and spatial control over protein binding; ii) the small size of the modification (typical caging groups are less than 500 Da in size); and iii) the possibility of generating the modified protein directly by the biosynthetic machinery within the cell (Courtney and Deiters, 2018). However, there are also challenges associated with their use. First, the suppression efficiencies are variable, context-dependent and may result in severe drops of protein yields, particularly in the case of multi-site incorporation (Amiram et al., 2015). Second, the introduction of non-native side-chains may affect protein folding, stability, binding and dynamics (Rogers et al., 2018; Kesgin-Schaefer et al., 2019; Chaudhari et al., 2023). Third, due to the usually large interfacial areas that are stabilized by multiple non-covalent interactions (hydrogen bonds, hydrophobic interactions, *etc.*), targeting PPI with single-residue perturbations is not straightforward, typically requiring extensive screening campaigns (Bridge et al., 2019; O'Shea et al., 2022). This contrasts with the photocontrol of interactions between proteins and small ligands or substrates, which typically depend on one or a reduced number of residues closely localized in space, e.g., an enzyme’s catalytic site (Nguyen et al., 2014; Zhou et al., 2020).

The interleukin 10 family, which includes IL-24, plays important roles in cell differentiation, apoptosis, and tumor growth, and thus represents an attractive therapeutic target (Chada et al., 2005; Cunningham et al., 2005). Disrupted cytokine-receptor signaling and JAK/STAT phosphorylation cascade may lead to a variety of diseases, such as skin conditions, cancers, and disorders affecting the immune system (Aaronson and Horvath, 2002; Hu et al., 2021). Members of the IL-10 are also involved in host-pathogen interactions, wound healing, and have potential in the treatment of respirator, inflammatory and autoimmune diseases (Iyer and Cheng, 2012; Traupe, 2017; Liu et al., 2023; Reis et al., 2023). To the best of our knowledge, there is only one example of inhibition of the interaction between IL-24 and its receptors, which is based on antibody binding to IL-20R1 and IL-22R1 (Chada et al., 2004). The light-dependent assembly between IL-24 and its receptors may be an alternative route toward the development of a new therapeutic strategy to combat such diseases and conditions.

### 4.2 Heterologous expression of interleukins and receptors containing ncAA

To obtain IL-24, the receptor IL-20R2, and their photoactivatable variants, we combine canonical and ncAA mutagenesis.

Depending on protein sequence and need for post-translational modifications, obtaining sufficient yields of stable proteins can be a serious bottleneck. Obtaining “difficult” proteins requires multiple expression attempts (Peleg and Unger, 2012) or cumbersome and cost-ineffective expression systems (Hopkins et al., 2010). On top of these effects, the incorporation of ncAA may cause further decreases in protein yields and alter protein stability or function in ways that are difficult to predict (Chaudhari et al., 2023). Indeed, all our proteins of interest are naturally N-glycosylated and bear disulfide bonds, which make them hard to produce in traditional *E. coli* expression systems. Production of wild-type IL-24 in high quantities is only possible with the co-expression of its interacting partner (Lubkowski et al., 2018). Overall, protein production renders the IL-24 signaling complex a very challenging target for (photo)xenoprotein engineering. Therefore, we adopted an alternative strategy to obtain active proteins while avoiding long and severe optimization trials based on structure- and sequence-based protein design using the 20 canonical amino acids (Goldenzweig et al., 2016). Such a bioinformatics design optimizes the amino acid sequence of IL-24 and IL-20R2 to stabilize their expression in bacteria, rendering them more suitable for functional studies. Both engineered variants, named IL-24B4 and IL-20R2D, are biologically active and support signal transduction through the JAK/STAT pathway.

Next, the light-inducible variants are made by adding a photocaged amino acid, NBY, into IL-24B4 and IL-20R2D by genetic code expansion technology. We looked for candidate residues in both interacting partners and found three target tyrosines. The single tyrosine mutant of IL-24B4, Y204NBY, did not express at sufficient and cost-effective levels for subsequent biophysical investigations by MST. Thus, IL-24B4 Y204NBY could only be studied in cell assays, which require relatively low amounts of material. In the case of the receptor, all ncAA variants including two single mutants and one double mutant were purified at the required amounts for all three methods employed in this work (MST, yeast display and human cell assays). NBY did not substantially affect protein conformation but it had a negative impact on protein’s thermal stability, particularly in the case of the double mutant.

### 4.3 Monitoring IL-24 – IL-20R2 interactions by biophysical methods and cell assays

IL-24B4 Y204NBY exhibits minimal albeit reproducible differences in cell signaling assays when comparing parental and caged variants.

We find one clear “hit” confirmed by both MST and yeast display: IL-20R2D Y70NBY shows low affinity for IL-24B4 and the interaction can be activated by UV light to reach native-like levels. This in line with previous studies reporting that a single NBY residue is sufficient to diminish PPI (Bridge et al., 2019; Jedlitzke et al., 2019; Joest et al., 2021; Jedlitzke and Mootz, 2022). Here we used an innovative approach of yeast surface display to gain better control over our experiment and see the residual concentrations. We mixed yeasts expressing IL-24B4 with a low amount of yeasts expressing its affinity matured counterpart, which has more than two orders of magnitude higher affinity (IL-24S). The analysis showed detectable but low, below saturation, binding signals of IL-24S with IL-20R2D Y70NBY (Supplementary Figure S10). Given the difference in binding affinity, the concentration of binding-capable ligands was below 1% of total concentration.

Unfortunately, due to limitations in our signaling assays, including both the choice of synthetase and the background signaling effect of IL-20R2D, we could not test the effect of IL-20R2D Y70NBY in human cells. It was previously reported that dimerization of IFNαR2 is sufficient for induction of interferon-regulated genes but not for full activity (Pattyn et al., 1999). Here, we show that purified IL-20R2D added to the cells can trigger signaling albeit at high concentrations, This problem may be partially circumvented by using general reporter cell lines and/or reporter assays based on gene expression (Mock et al., 2020; Cho et al., 2023) with further research needed. As an alternative to the genetic encoding of NBY in sequence-optimized truncated receptors recombinantly expressed in *E. coli*, one could biosynthetically incorporate photocaged tyrosines in full-length IL-20R2 receptors expressed in HeLa cells by using an orthogonal translation system derived from *Methanosarcina barkeri* (Arbely et al., 2012).

The more effective blocking of heterocomplex assembly with Y70NBY compared to Y204NBY can be rationalized in structural terms: Y70 of IL-20R2 is found at the core of the IL-24/IL-20R2 interface while Y204 of IL-24 is located in a flexible region at one edge of the interaction surface. We notice that the use of NBY to photocontrol IL-24-dependent PPI necessitates further improvements. Y70NBY and particularly Y204NBY do not fully prevent complex assembly, but rather shift the binding equilibrium towards higher concentrations (lower affinities). IL-24/IL-20R2 complex may still be formed and signal transduction started if large concentrations of photocaged binding partners are employed. In principle, substantial affinity differences between native and caged variants are preferred. The dynamical range is much narrower for IL-24B4 Y204NBY (3-fold difference in *EC*_*50*_) than for IL-20R2D Y70NBY (at least 300-fold difference in *K*_*d*_ according to both MST and yeast display). Larger dynamical ranges could be achieved through the installment of photocages on each partner, for instance Y70NBY in IL-20R2D and Y204NBY in IL-24B4. Although residues at the binding interface seem the natural choice for replacement, other distant sites may also influence PPI through allosteric communication, which has been reported for some interleukins (Bowman and Geissler, 2012; De Paula et al., 2020).

Strikingly, the two single mutants (IL-20R2 Y70 NBY and IL-20R2D Y74NBY) and the double mutant (IL-20R2D Y70NBY/Y74NBY) show clear signs of non-additive effects on protein binding. Y70NBY inhibits association with IL-24B4 while Y74NBY and Y70NBY/Y74NBY do not significantly alter protein-protein interactions. Non-additive interactions between mutations (epistasis) are common in proteins and play a crucial role in protein engineering (Reetz, 2013). There is no clear picture of the mechanisms that cause epistasis, although effects on protein stability, conformation and dynamics have all been invoked (Starr and Thornton, 2016). Our CD and thermal denaturing experiments suggest minimal differences among the mutants. All variants show similar secondary structure content. The melting temperature of the mutants is reduced with respect to the parental proteins and the Tm of the double mutant Y70NBY/Y74NBY is further reduced thus suggesting additive effects. Therefore, protein structure and thermal stability do not seem to be the major causes of the negative (antagonistic/deleterious) epistasis observed when combining Y70NBY and Y74NBY mutations. We hypothesize that the two mutations together alter IL-20R2D structural dynamics, a phenomenon that has been described for other proteins (Wagner et al., 1995; Acevedo-Rocha et al., 2021).

Interestingly, we found evidence that in some cases UV-irradiation alone reduced the binding affinity. Although we did not detect clear signs of photoinduced chemical modifications, e.g., photo-oxidation (the masses of IL-24B4 and IL-20R2D before/after UV are virtually identical), the affinity between IL-24B4 and IL-20R2 was reduced 6-fold (MST-based experiments) or 2-fold (yeast surface display experiments). This result implies that our UV irradiation protocol damages the proteins to a certain extent. We suggest that by carefully controlling the illumination conditions (wavelength, power density and time) such an undesired effect may be eliminated. Moreover, UV light can potentially damage nucleic acids depending on the dose used and it does not penetrate deeply into tissues (Kielbassa et al., 1997). Consequently, for *in vivo* applications requiring native-like binding affinities it would be beneficial to use other photocages. Suitable candidates would be the nitropiperonyl moiety, which features longer absorption wavelengths (Gautier et al., 2010; Luo et al., 2017) or coumarins, which can be activated by two-photon excitation in the near infrared range (Luo et al., 2014). Apart from photocaged ncAAs, which enable unidirectional OFF-to-ON switch of protein binding by light, one could use other ncAA. For instance, in applications requiring bidirectional photocontrol of protein binding (Jankovic et al., 2019; Myrhammar et al., 2020; Zhang et al., 2022), photoswitchable ncAA like azobenzene-phenylalanine constitute an excellent alternative (Bose et al., 2005; Luo et al., 2018; Israeli et al., 2021).

Overall, our results suggest the feasibility of regulating interactions involving protein partners from the interleukin family, IL-24 and IL-20R2, with UV light and photocaged tyrosines introduced at certain residue positions. In the future, other cytokine/receptor pairs and non-antibody-based protein scaffolds (Kolářová et al., 2021; Pham et al., 2021; Huličiak et al., 2023) may benefit from a similar approach and offer excellent interaction control.

## Data Availability

The original contributions presented in the study are publicly available. This data can be found here: https://doi.org/10.5281/zenodo.7877998.
