# Supplementary material for: Regulation of IL-24/IL-20R2 complex formation using photocaged tyrosines and UV light
Source: Front Mol Biosci. 2023 Jul 7;10:1214235. doi: 10.3389/fmolb.2023.1214235 (PMC10361524; doi:10.3389/fmolb.2023.1214235)
Supplement: Supplementary file 1 [file DataSheet1.PDF]

Supplementary Material

Regulation of IL-24/IL-20R2 complex formation using photocaged tyrosines and UV light

Phuong Ngoc Pham, Jiří Zahradník, Lucie Kolářová, Bohdan Schneider, Gustavo Fuertes*

*** Correspondence:** Corresponding Author: gustavo.fuertes@ibt.cas.cz

1 Supplementary text.

1.1 Note S1: Design of IL-20R2D

We applied the PROSS algorithm on two available structures in PDB, 6df3 (Lubkowski et al., 2018) and 4doh (Logsdon et al., 2012). The two structures differ with root mean square deviation (RMSD) value 0.945 Å across all 190 residue pairs, a value that is higher for certain flexible parts of protein including the main binding loop with helix (amino acids E69 – W88, RMSD 1.243 Å). Mutations in the cytokine binding site were permitted because our previous results showed that such an approach may lead to the identification of residues crucial for binding yet destabilizing the protein (Zahradník et al., 2019). In total, 21 and 20 stabilizing mutations were suggested in structure 6df3:H and 4doh:B, respectively. All mutations are listed in **table S2**. Approximately 50 % of predicted stabilizing mutations were robust in calculations and the same for both structures. 6 mutations were predicted only in 6df3:H, while 4 mutations were identified only in 4doh:B. We decided to incorporate both, robust mutations and mutations specific for IL-20R2, in IL-24 signaling complex structure. Manual inspection revealed that one of these suggested mutations, T79R, may stabilize the binding helix but be in conflict with the binding itself. Another additional mutation, S90Q, may influence the binding by changes in binding loop flexibility. Both mutations were omitted in the designed protein. A new disulfide bond was predicted between A59C and L117C and these mutations were allowed. Although this disulfide bond was identified in fish species, it is not present in mammalian sequences. Supporting this observation, a simple energy minimization procedure in UCSF Chimera showed marginal changes required for disulfide bond formation.

2 Supplementary tables.

2.1 Table S0.

Material, reagent or software	*Source*	*Catalog number*
**Proteins and antibodies**		
Protease Inhibitor Cocktail	Sigma-Aldrich	P8340
Phosphatase Inhibitor Cocktail 2	Sigma-Aldrich	P5726
Phosphatase Inhibitor Cocktail 3	Sigma-Aldrich	P0044
Human IFN-a 2b Recombinant Protein®	Cell Signalling Technology	#36000
Monoclonal Anti-a-Tubulin, clone DM1A in mouse	Sigma-Aldrich	T9026
Phospho-Stat3 (Tyr705) (D3A7) XP Rabbit mAb	Cell Signalling Technology	#9145
Goat Anti-Rabbit IgG (H + L)-HRP Conjugate	Bio-rad	#1706515
Goat Anti-Mouse IgG (H + L)-HRP Conjugate	Bio-rad	#1706516
**Kits**		
jetPRIME® Versatile DNA/siRNA Transfection Reagent	Polyplus transfection	114-07
QPRO-BCA Kit Standard	Cyanagen	PRTD1
His-Tag Labeling Kit – RED-Tris-NTA 2nd Generation	Nanotemper	MO-L018
**Chemicals**		
Igepal® CA-630	MP Biomedicals	11452731
HisPur™ Ni-NTA Resin	Thermo Scientific	88221
Strep-Tactin®XT 4Flow® resin	IBA	2-5010-025
Strep-Tactin®XT elution buffer	IBA	2-1042-025
O-(2-Nitrobenzyl)-L-tyrosine Hydrochloride	Accela	207727-86-4
L-(+)-Arabinose	Formedium	ARA001
Bovine serum albumin (BSA)	Biosera	PAO214C0O4
DMEM High Glucose	Biosera	MS01N61007
Immobilon® Forte Western HRP Substrate	Millipore	WBLUF0100
**Other materials**		
VWR Tissue culture Plate	Avantor	10062-892
Trans-Blot Turbo Mini 0.2 µm Nitrocellulose Transfer Packs	Bio-rad	1704158

Key resources table.

2.2 Table S1.

Protein	Sequence	Mutations relative to WT
IL-24B4[a]	MSKHHHHSGHHHTGHHHHSGSHHHSGSAAGGEEDKKPAGGEGGGAHINLKVKGQDGNEVFFRIKRSTQLKKLMNAYCDRQSVDMTAIAFLFDGRRLRAEQTPDELEMEDGDEIDAMLHQTGGA**FHFGPCRVEGVVPQELWEAFWAVRDTLQAQDNITDVRLLRAEVLQNVSDAESCYLVHQLLRFYLDTVFKNYHNKTAELRTLKSFSTLANNFVLIVSDLQPCQEQNMCSSREEAHRRFLQFQRAFEQLDVEAAATKALGEIDILLTWMQKFYQL**GGSWSHPQFEK	29: Q60R, K62E, K68E, K77R, M80L, S88D, A89V, Q93R, Q94A, T111Q, E114R, K118D, R127K, V129A, V131L, Q150D, S154C, N157Q, E158N, F160C, I162S, D164E, S165E, L172Q, R174Q, K178E, L186A, V193I, K205Q
IL-24B4[b]	MQKLISEEDLTSLEDDPEAAYTTRGGKIPAS**QEFHFGPCRVEGVVPQKLWEAFWAVRDTLQAQDNITDVRLLRAEVLQNVSDAESCYLVHQLLRFYLDTVFKNYHNKTAELRTLKSFSTLANNFLQIRKDLQPCQEQNMCSSREEAHRRFLQFQRAFEQLDVEAAATKALGEIDILLRWMQKFYQL**	29: Same as above
IL-24S[b]	MQKLISEEDLTSLEDDPEAAYTTRGGKIPAS**QEFHFGPCRVKRVVPQELWEAFWAVRDTLRAQDNITDVRLLRAEVLQNVSDAESCYLVHQLLRFYLGTVFKNYHDKTAELRTLKSLSTLANNFYIIVRDLQQCQEQNMCSSREEAHRRFLQFQRAFEQLDVEAAATKALGEIDILLRWMQKFYQL**	40: Q60R, G63R, K68E, K77R, M80L, Q81R, Q85N, S88D, A89V, Q93R, Q94A, Q99N, T111Q, E114R, K118G, H124Y, Q126D, R127K, V129A, V131L, F137L, V145Y, L146I, S149R, Q150D, P153Q, S154C, N157Q, E158N, F160C, I162S, D164E, S165E, L172Q, R174Q, K178E, L186A, V193I, T198R, K205Q
IL-20R2D[a]	MSKHHHHSGHHHTGHHHHSGSHHHSGSAAGGEEDKKPAGGEGGGAHINLKVKGQDGNEVFFRIKRSTQLKKLMNAYCDRQSVDMTAIAFLFDGRRLRAEQTPDELEMEDGDEIDAMLHQTGGA**LPAPQNLSVLSVNMKHLLTWSPVICPGETVYYSVEYQGEYESLYRSHIWIPSSWCQLTTGPWCDITDDITATVPYNLRVRAECGSQTSAWSTLKHPFNRQDTILTPPGMEVTKDG**	23: T46V, M53T, A59C, T79R, S90Q, E93T, E96W, V99I, T116E, L117C, L126T, N134Q, S135D, R140P, I145V, L166Y, A168F, E177Q,

	**FHLVIELEDLGPQFEFYVFYWRREPGAQVHVKVV RSGGIPVHLETMEPGAEYCVKAQTYVKAIGRYSA FSQTQCV**GGSWSHPQFEK	E178V, M182V, A200E, F208Y, E222Q
IL-20R2[c]	**LPAPQNLSVLSTNMKHLLMWSPVIAPGETVYYSV EYQGEYESLYTSHIWIPSSWCSLTEGPECDVTDD ITATVPYNLRVRATLGSQTSAWSILKHPFNRNST ILTRPGMEITKDGFHLVIELEDLGPQFEFLVAYW RREPGAEEHVKMVRSGGIPVHLETMEPGAAYCVK AQTFVKAIGRYSAFSQTECVEVQGEAIPLVLALF AFVGFMLILVVVPLFVWKMGRLLQYSCCPVVVLP DTLKITNSPQKLISCRREEVDACATAVMSPEELL RAWIS**	0
IL-20R2DC[c]	**LPAPQNLSVLSVNMKHLLTWSPVICPGETVYYSV EYQGEYESLYRSHIWIPSSWCQLTTGPWCDITDD ITATVPYNLRVRAECGSQTSAWSTLKHPFNRQDT ILTPPGMEVTKDGFHLVIELEDLGPQFEFYVFYW RREPGAQVHVKVVRSGGIPVHLETMEPGAEYCVK AQTYVKAIGRYSAFSQTQCVEVQGEAIPLVLALF AFVGFMLILVVVPLFVWKMGRLLQYSCCPVVVLP DTLKITNSPQKLISCRREEVDACATAVMSPEELL RAWIS**	23: T46V, M53T, A59C, T79R, S90Q, E93T, E96W, V99I, T116E, L117C, L126T, N134Q, S135D, R140P, I145V, L166Y, A168F, E177Q, E178V, M182V, A200E, F208Y, E222Q
IL-20R2D[d]	**ALPAPQNLSVLSVNMKHLLTWSPVICPGETVYYS VEYQGEYESLYRSHIWIPSSWCQLTTGPWCDITD DITATVPYNLRVRAECGSQTSAWSTLKHPFNRQD TILTPPGMEVTKDGFHLVIELEDLGPQFEFYVFY WRREPGAQVHVKVVRSGGIPVHLETMEPGAEYCV KAQTYVKAIGRYSAFSQTQCV**	23: Same as above
IL-22R1[c]	**MRTLLTILTVGSLAAHAPEDPSDLLQHVKFQSSN FENILTWDSGPEGTPDTVYSIEYKTYGERDWVAK KGCQRITRKSCNLTVETGNLTELYYARVTAVSAG GRSATKMTDRFSSLQHTTLKPPDVTCISKVRSIQ MIVHPTPTPIRAGDGHRLTLEDIFHDLFYHLELQ VNRTYQMHLGGKQREYEFFGLTPDTEFLGTIMIC VPTWAKESAPYMCRVKTLPDRTWTYSFSGAFLFS MGFLVAVLCYLSYRYVTKPPAPPNSLNVQRVLTF QPLRFIQEHVLIPVFDLSGPSSLAQPVQYSQIRV SGPREPAGAPQRHSLSEITYLGQPDISILQPSNV PPPQILSPLSYAPNAAPEVGPPSYAPQVTPEAQF PFYAPQAISKVQPSSYAPQATPDSWPPSYGVCME GSGKDSPTGTLSSPKHLRPKGQLQKEPPAGSCML GGLSLQEVTSLAMEESQEAKSLHQPLGICTDRTS DPNVLHSGEEGTPQYLKGQLPLLSSVQIEGHPMS**	0

	**LPLQPPSRPCSPSDQGPSPWGLLESLVCPKDEAK SPAPETSDLEQPTELDSLFRGLALTVQWES**	

Protein sequences used in this study. Tag-free sequences are indicated in bold. Mutated residues are underlined. The three tyrosine (Y) residues targeted for replacement by ortho-nitrobenzyl-tyrosine (NBY) are labeled in green. These include Y204 in IL-24B4, plus Y70 and Y74 in IL-20R2D. [a] Variants used for microscale thermophoresis. [b] Variants used for yeast display. [c] Variants used for signaling assays in human cells. [d] Original variant used to show that the feasibility of expressing engineered versions of IL-20R2D in *E. coli* (figure S3B).

2.3 Table S2.

#	Primer	Sequence (5' to 3')
1	*Rbs-HisSumo-for*	CAATTCCCCTCTAGAAATAATTTTGTTTAACTTTAAGAAGGAGATATAC CATGAGCAAGC
2	*IL24B4-Strep-rev*	TGGTGGTGCTCGAGTCACTTTTCGAACTGCGGGTGGCTCCAGCTGCCGC CCAGTTGATAAAACTTTTGCATCCAGGTAAGAAGG
3	*IL20R2-string-for*	GATGCGTCCGGCGTAGAGG
4	*IL20R2-string-rev*	CAGCCGGATCTCAGTGGTGG
5	*IL20R2-Y70tag-for*	GGAATAGCAGGGCGAGTACGAGAGCC
6	*IL20R2-Y70tag-rev*	GCCCTGCTATTCCACGCTGTAGTACACTGTCTCG
7	*IL20R2-Y74tag-for*	CGAGTAGGAGAGCCTGTACCGCAGCC
8	*IL20R2-Y74tag-rev*	GCTCTCCTACTCGCCCTGGTATTCCACG
9	*IL20R2-Y74tag-rev2*	GCTCTCCTACTCGCCCTGCTATTCCACG
10	*IL24-Y204tag-for*	GTTTTAGCAACTGGGCGGCAGCTG
11	*IL24-Y204tag-rev*	CAGTTGCTAAAACTTTTGCATCCAGGTAAGAAGGATGTCG
12	*IL20R2_for*	CAAGCTGGCTAGCGTTTAAACTTAAGCTTGCCACCATGCAGACTTTCAC AATGGTTCTAGAAGAAATCTGG
13	*IL20R2-rev*	CGGGCCCTCTAGACTCGAGCGGCCGCTCATGAGATCCAGGCCCTGAGGA GTTCC
14	*IL22R1-for*	CAAGCTGGCTAGCGTTTAAACTTAAGCTTGCCACCATGAGGACGCTGCT GACCATCTTGAC
15	*IL22R1-rev*	CGGGCCCTCTAGACTCGAGCGGCCGCTCAGGACTCCCACTGCACAGTCA G

Oligos used in this study.

2.4 Table S3.

Proteins and conditions	Calculated mass (Da)	Experimental mass (Da)
IL-24B4	32,497	32,490
IL-24B4 + UV	32,497	32,490
IL-2B4 Y204NBY	32,632	32,630
IL-2B4 Y204NBY + UV	32,497	32,490
IL-20R2D	36,158	36,160
IL-20R2D +UV	36,158	36,160
IL20R2D Y70NBY	36,293	36,290
IL20R2D Y70NBY + UV	36,158	36,160
IL-20R2D Y74NBY	36,293	36,290
IL-20R2D Y74NBY +UV	36,158	36,160
IL-20R2D Y70NBY/Y74NBY	36,428	36,430 (16,910, 15,690)
IL-20R2D Y70NBY/Y74NBY + UV	36,158	36,180 (16,910, 15,690)

Protein quality control and UV-decaging efficiency by mass spectrometry. All masses (calculated and experimental) are reported as average masses.

2.5 Table S4.

	Secondary structure (%)				
Variant	**α-helix**	**β-sheet antiparallel**	**β-sheet parallel**	**Turn**	**Others**
IL-24B4	32.4	10.5	0	12.1	45
IL-24B4 Y204	30.5	13.6	0	12.6	43.3
IL-20R2D	7.6	33.5	0	16.1	42.8
IL-20R2D Y70NBY	11.1	29.1	1.7	13.7	44.4
IL-20R2D Y74NBY	9.6	32.3	0.6	12.3	45.2
IL-20R2D Y70NBY/Y74NBY	8.6	29.9	2.3	11.7	47.5

Secondary structure content of IL-24 and IL-20R2 variants. Estimations were done based on the recorded circular dichroism spectra in the wavelength range from 200 to 250 nm using the BeStSel server.

2.6 Table S5.

Variant	**Melting temperature (°C)**
IL-24B4	64.4±0.3
IL-24B4 Y204NBY	57.7±0.1
IL-20R2D	44.9±0.2
IL-20R2D Y70NBY	37.8±0.1
IL-20R2D Y74NBY	42.6±0.1
IL-20R2D Y70NBY/Y74NBY	35.3±0.2

Thermal stability of IL-24 and IL-20R2 variants. Values are indicated as the mean ± s.d. of two replicate measurements of the same samples. Estimations of melting temperatures (T_m) were done based on differential scanning fluorimetry using the first derivative of the tryptophan fluorescence emission ratio at 350 nm and 330 nm (with excitation at 280 nm).

2.7 Table S6.

**Proteins and conditions**	**Expected molecular weight (kDa)**	**Apparent molecular weight (kDa)**
IL-24B4	32	44
IL-24B4 + UV		48
IL-2B4 Y204NBY		43
IL-2B4 Y204NBY + UV		46
IL-20R2D	36	53
IL-20R2D +UV		55
IL20R2D Y70NBY		43
IL20R2D Y70NBY + UV		52
IL-20R2D Y74NBY		42
IL-20R2D Y74NBY +UV		51
IL-20R2D Y70NBY/Y74NBY		42
IL-20R2D Y70NBY/Y74NBY + UV		51

Molecular weights estimated from size-exclusion chromatography. Proteins at a concentration of ~0.5 mg/mL were injected on a Superdex 75 Increase gel filtration column. The main peak was pooled, concentrated, irradiated with UV light and re-injected into the column. A calibration curve was constructed by running three proteins of known molecular weight (bovine serum albumin, carbonic anhydrase and cytochrome c) and then interpolating the ratio between the elution volume and the void volume (Vo). Vo was determined as the elution volume of dextran blue. Elution buffer was in all cases 50 mM Tris 100 mM NaCl pH=8.0.

3 Supplementary figures

3.1 Figure S1.

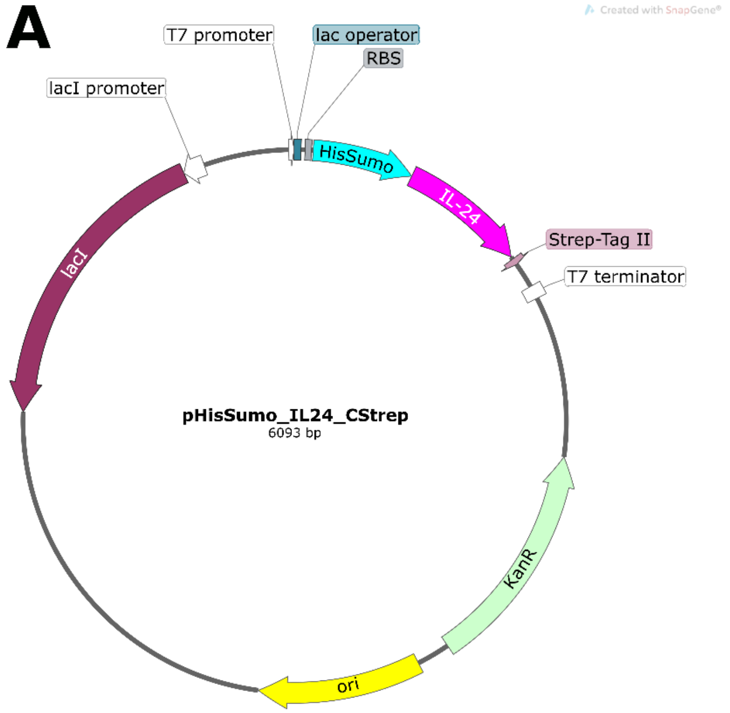

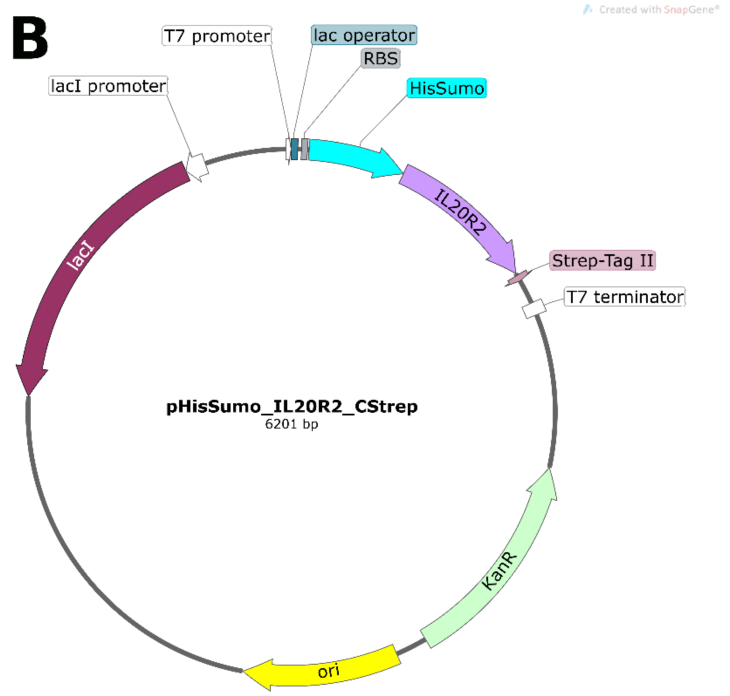

Plasmids created in this study. (A) pHisSumo_IL-24B4_Cstrep. (B) pHisSumo_IL-20R2D_CStrep.

3.2 Figure S2.

Position		52	53	54	55	56	57	58	59	60	61	62	63	64	65	66	67	68	69	70	71	72	73	74	75	76	77	78	79	80	81	82	83	84	85	86	87	88	89	90
IL-24wt PDB: 6df3	1	Q	E	F	H	F	G	P	C	Q	V	K	G	V	V	P	Q	K	L	W	E	A	F	W	A	V	K	D	T	M	Q	A	Q	D	Q	I	T	S	A	R
IL-24P7	2		A							R		E															R			L								D	V	
IL-24B PDB: 6gg1	3		A							R		E															R			L								D	V	
IL-24affi1	4									R		E						E									R			L								D	V	
IL-24affi2	5									R		E	R					E									R			L	R							D	V	
IL-24affiA	6									R		K	R					E									R			L	R							D	V	
IL-24S	7									R		K	R					R									R			L	R							D	V	
																																			N					

		91	92	93	94	95	96	97	98	99	100	101	102	103	104	105	106	107	108	109	110	111	112	113	114	115	116	117	118	119	120	121	122	123	124	125	126	127	128	129
	1	L	L	Q	Q	E	V	L	Q	Q	V	S	D	A	E	S	C	Y	L	V	H	T	L	L	E	F	Y	L	K	T	V	F	K	N	H	H	Q	R	T	V
	2			R	A					N												Q			R				D						Y		N	K		A
	3			R	A																	Q			R				D						Y			K		A
	4			R	A																	Q			R				D						Y			K		A
	5			R	A																	Q			R				D						Y			K		A
	6			R	A																	Q			R				G			R			Y		S	K		A
	7			R	A																	Q			R				G			F			Y		D	K		A
										N																											N			

IL-20RB distance		130	131	132	133	134	135	136	137	138	139	140	141	142	143	144	145	146	147	148	149	150	151	152	153	154	155	156	157	158	159	160	161	162	163	164	165	166	167	168
3 Å	1	E	V	R	T	L	K	S	F	S	T	L	A	N	N	F	V	L	I	V	S	Q	L	Q	P	S	Q	E	N	E	M	F	S	I	R	D	S	A	H	R
4 Å	2		L														L	Q		R	K	D				C			Q	N		C		S		E	E			
5 Å	3		L																			D				C			Q	N		C		S		E	E			
6 Å	4		L														Y	I				D				C			Q	N		C		S		E	E			
	5		L														Y	I				D				C			Q	N		C		S		E	E			
	6		L						L								Y	I			S	D			Q	C			Q	N		C		S		E	E			
	7		L						L								Y	I		V	R	D			Q	C			Q	N		C		S		E	E			

IL-22RA distance		169	170	171	172	173	174	175	176	177	178	179	180	181	182	183	184	185	186	187	188	189	190	191	192	193	194	195	196	197	198	199	200	201	202	203	204	205	206
3 Å	1	R	F	L	L	F	R	R	A	F	K	Q	L	D	V	E	A	A	L	T	K	A	L	G	E	V	D	I	L	L	T	W	M	Q	K	F	Y	K	L
4 Å	2				Q		Q				E								A							I					R							Q	
5 Å	3				Q		Q				E								A							I					R							Q	
	4				Q		Q				E								A							I					R							Q	
	5				Q		Q				E								A							I					R							Q	
	6				Q		Q				E								A							I					R							Q	
	7				Q		Q				E								A							I					R							Q	

IL-24 affinity maturation towards IL-20R2D. The mutations denoted in black were incorporated previously during the stabilization process (Zahradník et al., 2019). Mutations highlighted in red were dominantly accumulated (>70 % of clones) during rounds of directed evolution by yeast display. Variants bearing selected mutations were named IL-24affi1, 2, and A. The best variant obtained at the end of the process was labeled as IL-24S. Green Ns show positions of N-glycosylations that were mutated to glutamines.

3.3 Figure S3.

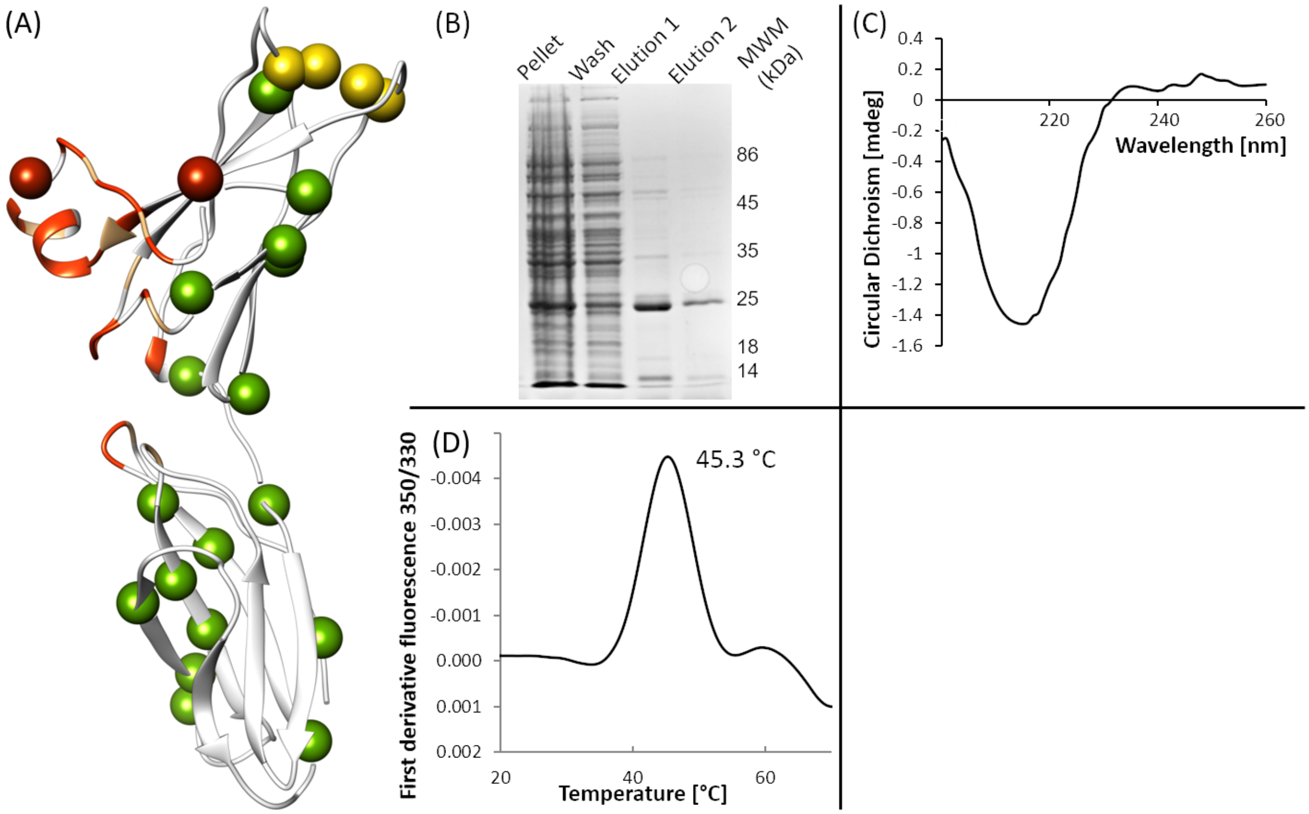

IL-20R2D design and expression. (A) Molecular model of IL-20R2 (PDB 6DF3) highlighting the position of the 23 residues predicted to improve protein solubility and stability. The color code is: yellow - new cysteine bridge introduced into IL20RBD; Orange is within 5Å from the cytokine; Red spheres - mutations in close proximity to binding interface; Green spheres - mutations introduced by our design. (B) SDS-PAGE showing the successful expression and purification by immobilized metal (Ni^{2+}) affinity chromatography of the designed variant (IL-20R2D) in *E. coli*. (C) The circular dichroism spectra of purified IL-20R2D suggests qualitatively that the protein is well-folded protein and has predominantly β-sheet content. (D) The thermal denaturation curve of purified IL-20R2D, based on the intrinsic tryptophan fluorescence, indicates a melting temperature of ~45 °C.

3.4 Figure S4.

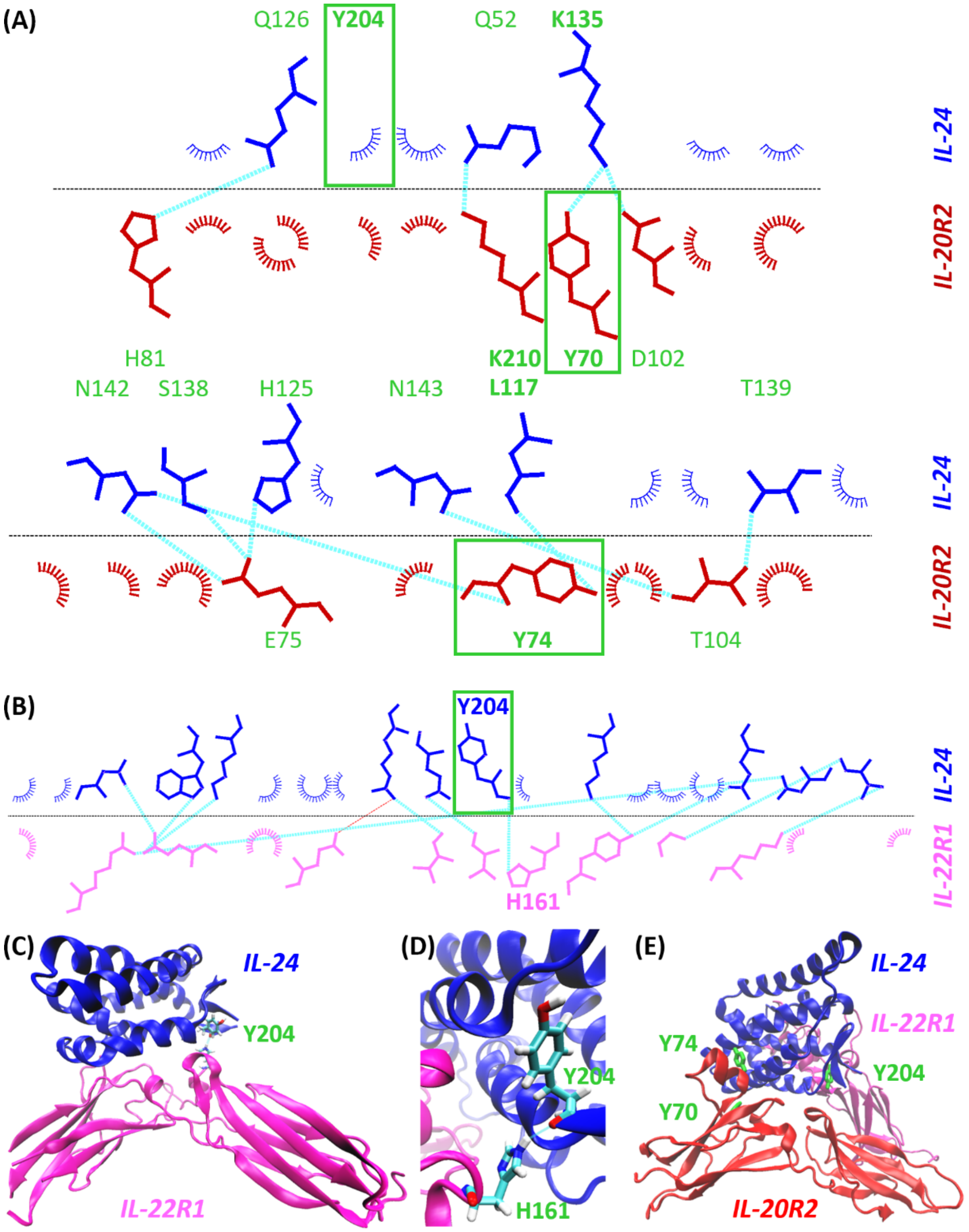

Selection of target residues for substitution with photocaged counterparts. (A) Scheme of the interaction interface between IL-24 (blue) and IL-20R2 (red). (B) Scheme of the interaction interface between IL-24 (blue) and IL-22R1 (magenta). In (A) and (B), hydrogen bonds are indicated as cyan dashed lines. Residues that make contacts through Van der Waals interactions are indicated as semi-circles. The chosen tyrosine residues (Y204, Y70, Y74) are highlighted in a green box. (B) 3D model of the complex between IL-24 and IL-22R2 based on X-ray crystallography (PDB 6DF3). (C) Close-up view of the H-bond between tyrosine204 (IL-24) and histidine161 (IL-22R1). (D) 3D model of the ternary complex between IL-24, IL-22R1, and IL-20R2 (red) based on X-ray crystallography (PDB 6DF3).

3.5 Figure S5.

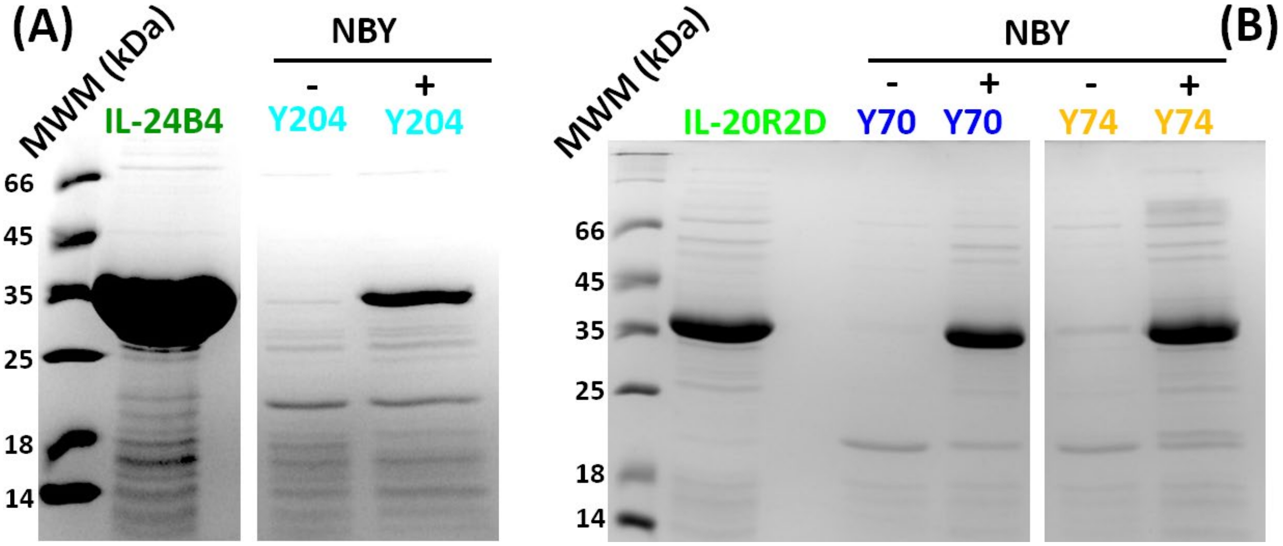

Expression and purification of parental and caged interleukins. (A) SDS-PAGE of IL-24B4 Y204TAG with (1 mM) and without added non-canonical amino acid NBY in TB medium. (B) SDS-PAGE of IL-20R2D Y70TAG and IL-20R2D Y74TAG with (1 mM) and without added non-canonical amino acid NBY in TB medium. All shown proteins retain the SUMO tag and the expected molecular weights are ~32 kDa and ~37 kDa for IL-24B4 and IL-20R2D variants, respectively.

3.6 Figure S6.

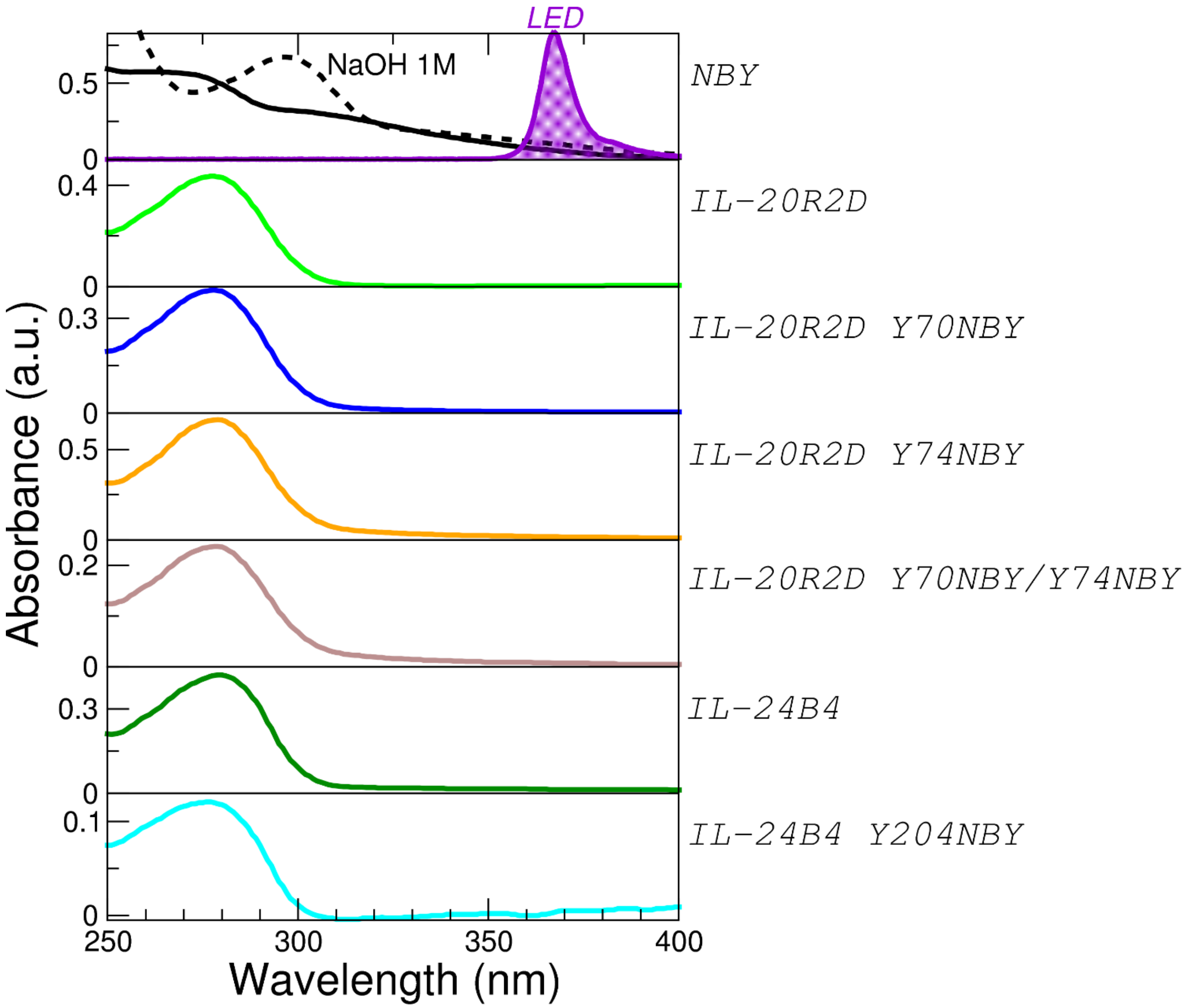

UV/Visible spectra of parental and caged interleukins/receptors. All spectra have been recorded in Tris 50 mM NaCl 100 mM pH=8 unless otherwise indicated. The spectral output of the LED used for UV-decaging experiments is shown on the top panel.

3.7 Figure S7.

(A)

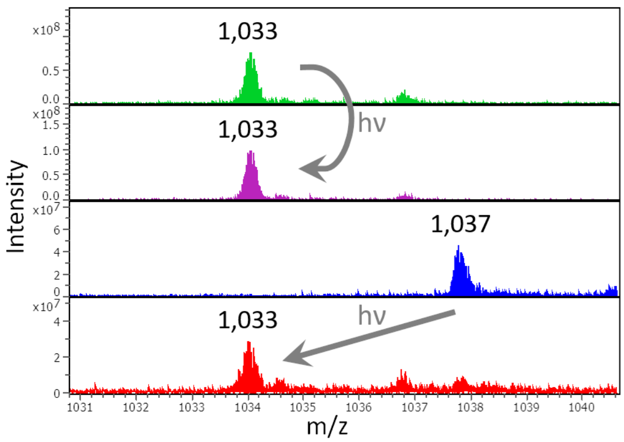

(B)

Protein	Calculated mass (Da)	Measured mass (Da)
IL-20R2D	36,136	36,137
IL-20R2D +UV	36,136	36,136
IL-20R2D Y70NBY	36,271	36,269
IL-20R2D Y70NBY +UV	36,136	36,136

Analysis of the effect of protein UV irradiation by high-resolution mass spectrometry. (A) Mass-to-charge ratio of the most abundant ion (35 positive charges). (B) Deconvoluted monoisotopic masses along with expected masses. For both, green represents IL-20R2D before UV irradiation (λ=365 nm, 5 min at 100 mW), purple represents IL-20R2D after UV, blue represents IL-20RD Y70NBY before UV, and red represents IL-20R2D Y70NBY after UV. Notice that IL-20R2D Y70NBY is converted back to IL-20R2D by UV light.

3.8 Figure S8.

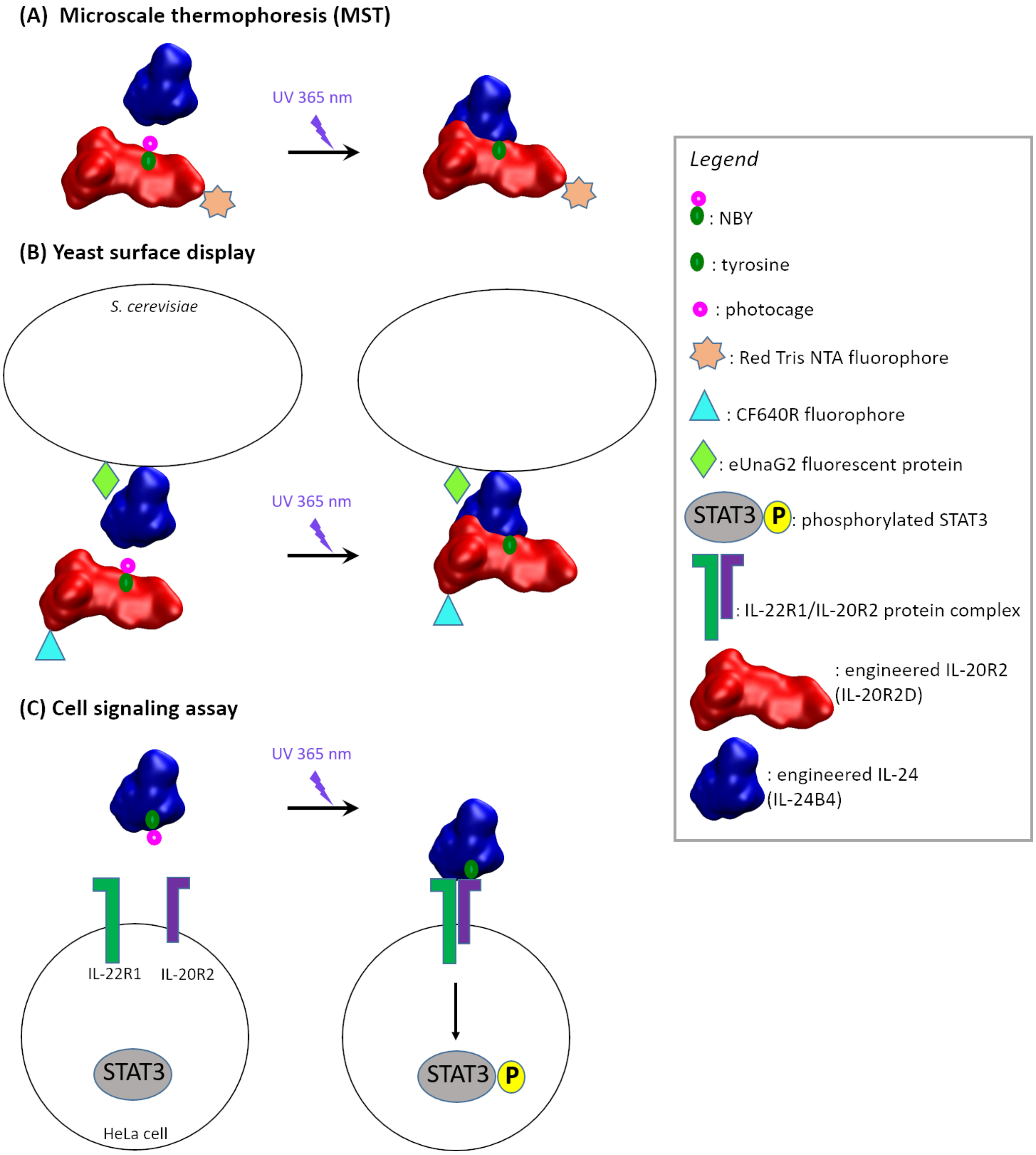

Scheme of the three assays used in this study to measure interleukin/receptor interactions. (A) Microscale thermophoresis to monitor binding between IL-24B4 and fluorescently labeled IL-20R2D in solution. (B) Yeast surface display followed by flow cytometry to monitor binding between fluorescently tagged IL-24B4 expressed on the surface of yeast cells and soluble fluorescently labeled IL-20R2D. (C) Signaling assays in HeLa cells to monitor the ability of IL-24B4 to trigger a phosphorylation cascade through IL-22R1/IL-20R2 heterodimer. Phosphorylated STAT3 (pSTAT3) is detected by immunoblotting with anti-pSTAT3 antibody and chemiluminiscence. The photocaged proteins are irradiated in solution and then incubated with yeast (panel B) or human cells (panel C).

3.9 Figure S9.

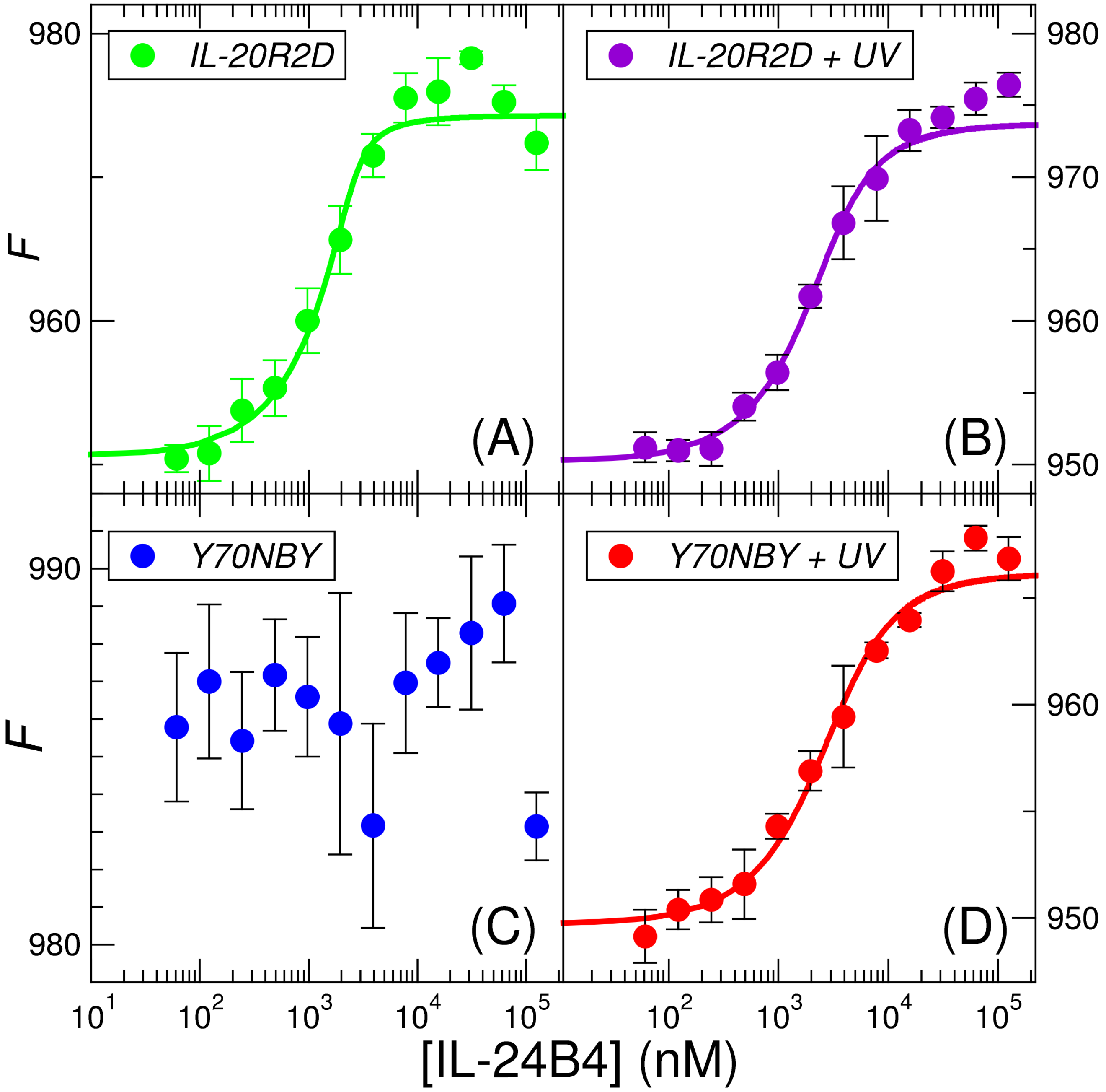

Estimation of binding affinities by microscale thermophoresis. (A) Non-normalized fluorescence changes (*F*) as a function of IL-24B4 concentration. Solid lines are fits to equation 1 in order to retrieve the dissociation contants (K_d, see **table 1**). (A) IL-20R2D before UV irradiation (green, λ=365 nm, 5 min at 100 mW). (B) IL-20R2D after UV (purple). (C) IL-20R2D Y70NBY before UV (blue). (D) IL-20R2D Y70NBY after UV (red).

3.10 Figure S10.

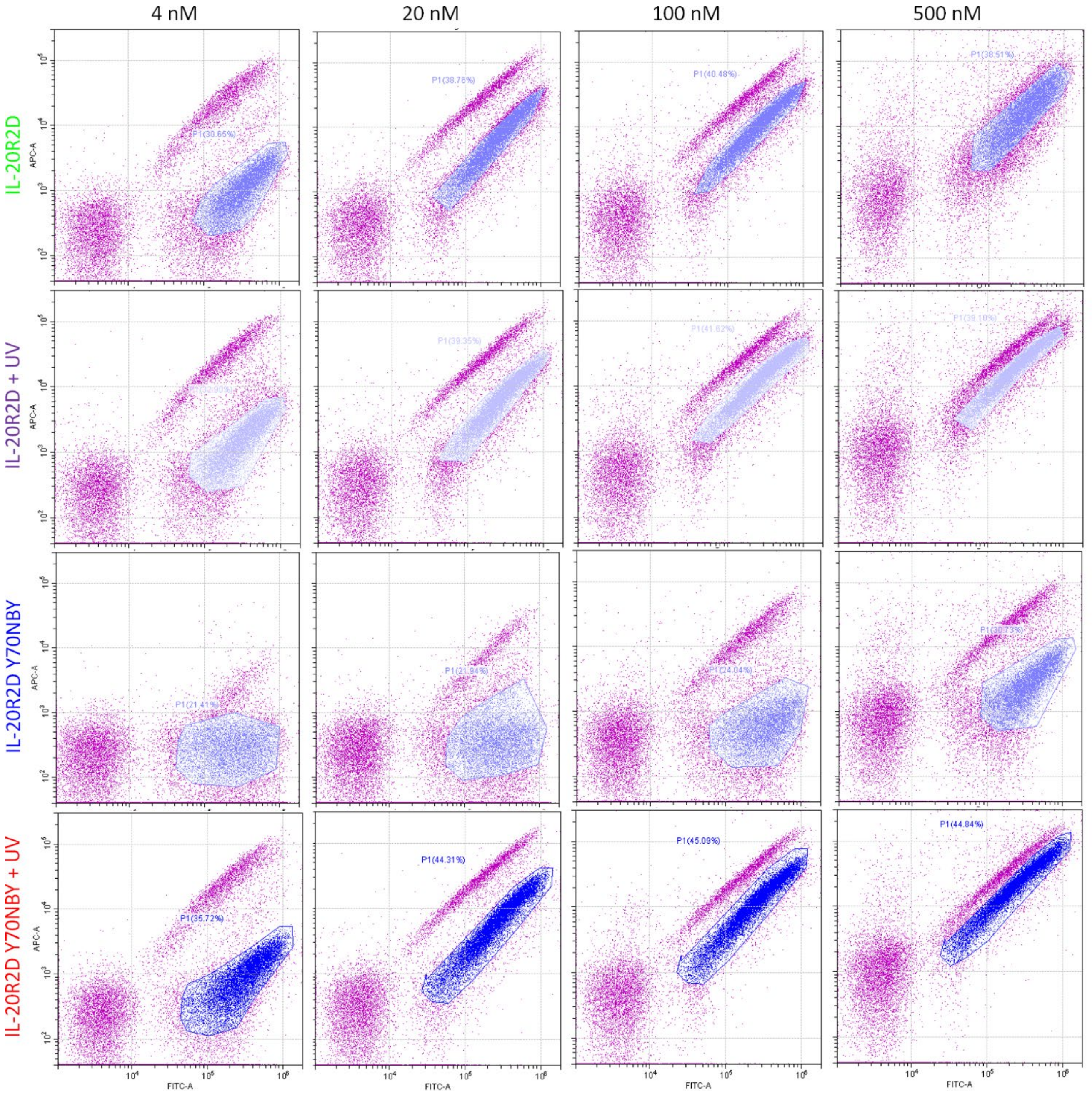

Estimation of binding affinities by yeast surface display. Fluorescence-activated cell sorting dot plots of “red” APC fluorescence channel (IL-20R2D or IL-20R2D Y70NBY labeled with CF640 added at the concentrations indicated on top of the graphs) *vs.* “green” FITC fluorescence channel (IL-24B4 fused to eUnaG2 in pJYDC vector). The tested yeast populations were mixed with yeasts expressing affinity matured IL-24S with 300-times tighter binding to IL-20R2D (approx. 5 % of the total cell number). This population allows for detection of residual signals of binding and offer *in situ* sensitive positive control without largely affecting the measured population. UV irradiation (λ=365 nm, 5 min at 100 mW). Apparent dissociation constant (K_d) values are reported in **table 1**. Populations of IL-24B4 are highlighted by gating.

3.11 Figure S11.

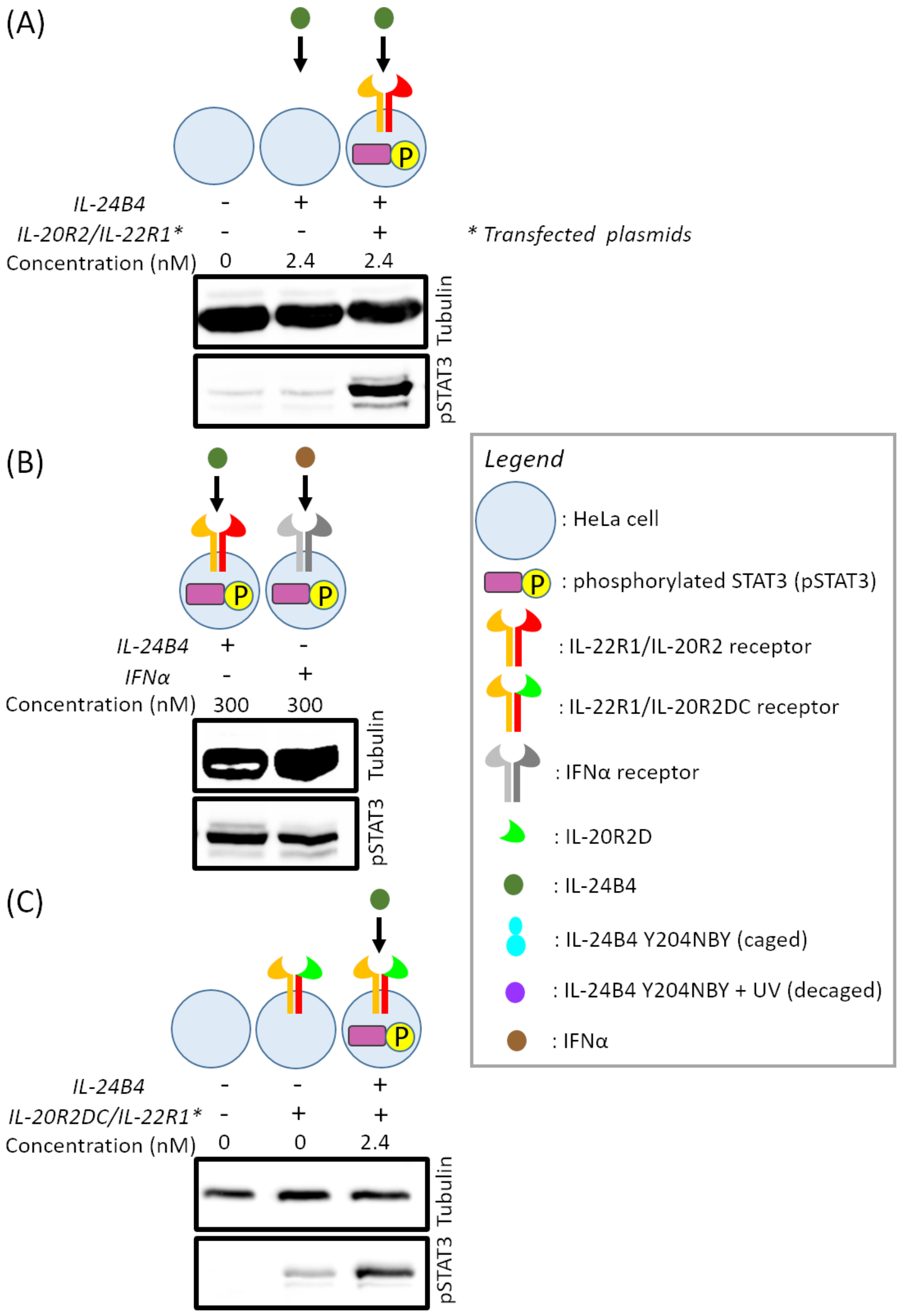

Effect of the engineered interleukin/receptor variants on the JAK/STAT signaling pathway (part I). Panels (A) to (C) show Western blots of phosphorylated STAT3 (pSTAT3) and tubulin upon incubation of caged/decaged variants for 30 minutes with live HeLa cells at the indicated concentrations and conditions. (A) Effect of exogenously added IL-24B4 on HeLa cells with or without plasmids encoding cognate receptors. (B) Effect of exogenously added interferon-α (IFNα) on HeLa cells expressing cognate receptors on their surface. (C) Effect of chimeric IL-20R2 (extracellular domain replaced by IL-20R2D) expressed on the surface of HeLa cells.

3.12 Figure S12.

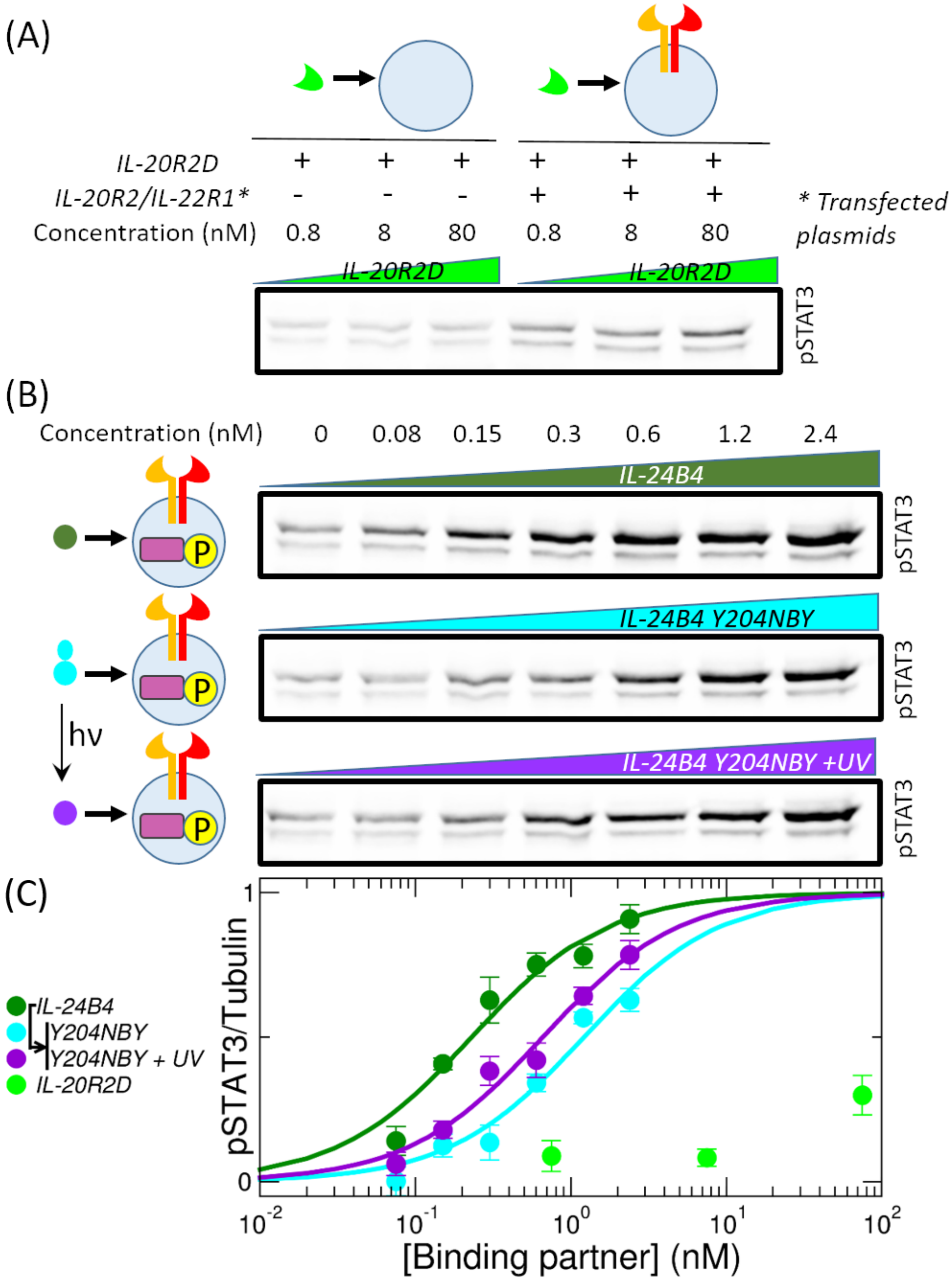

Effect of the engineered interleukin/receptor variants on the JAK/STAT signaling pathway (part II). Panels (A) and (B) show Western blots of phosphorylated STAT3 (pSTAT3) upon incubation of interleukin variants for 30 minutes with live HeLa cells at the indicated concentrations and conditions. (A) Effect of exogenously added IL-20R2D on HeLa cells with or without plasmids encoding cognate receptors. (B) Effect of exogenously added protein variants (IL-24B4, IL-B4B4 Y204NBY before UV, IL-24B4 Y204NBY after UV, and IL-20R2D) on HeLa cells expressing cognate receptors on their surface. (C) Plots of pSTAT3 band intensities (normalized by the corresponding tubulin band intensities) as a function of the concentration of added binding partner. Solid lines are fits to equation 3 in order to retrieve EC_{50} values (**table 1**). See legend in figure S11.
