# Supplementary figures and images for: Regulation of IL-24/IL-20R2 complex formation using photocaged tyrosines and UV light
Source: Front Mol Biosci. 2023 Jul 7;10:1214235. doi: 10.3389/fmolb.2023.1214235 (PMC10361524; doi:10.3389/fmolb.2023.1214235)

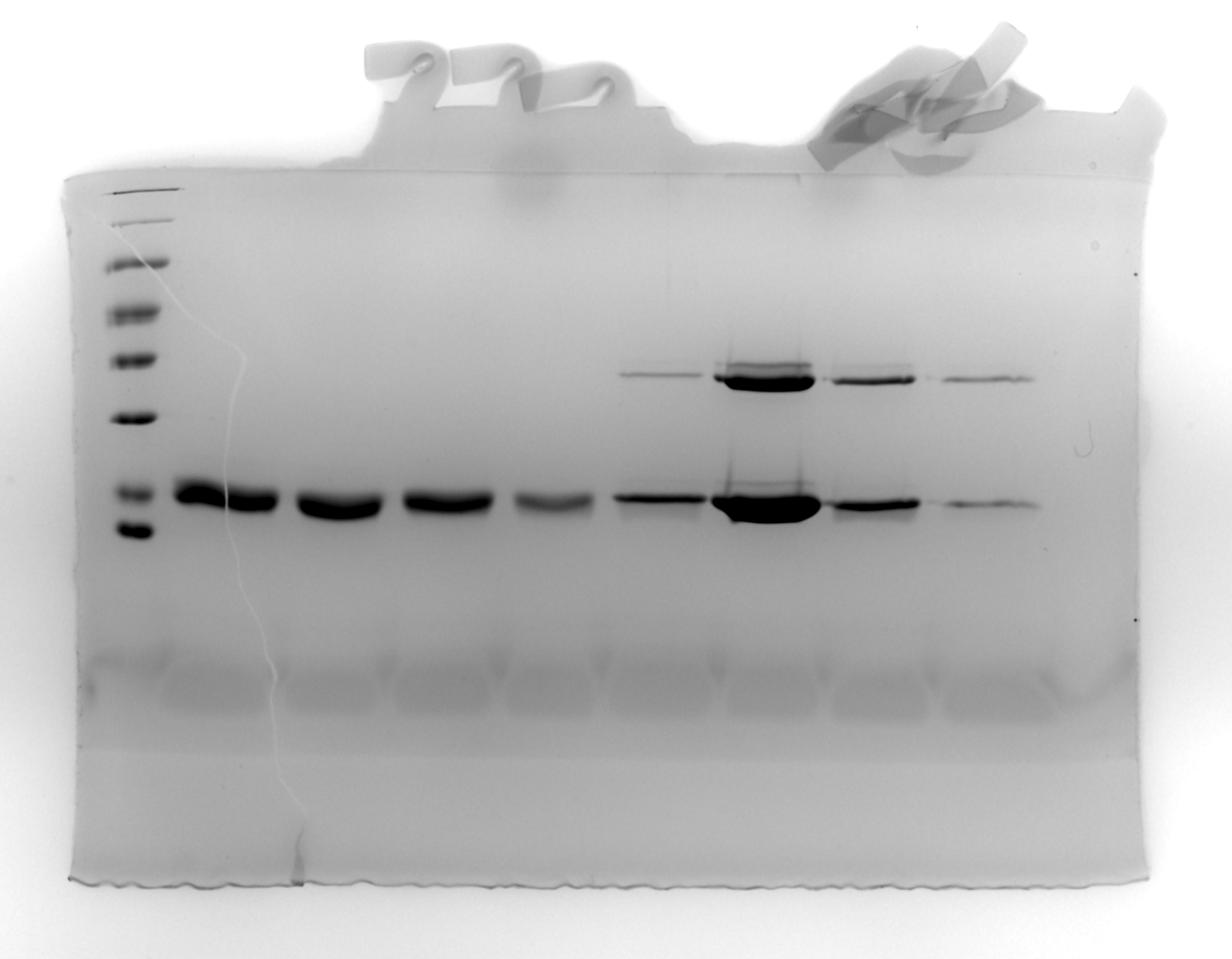

Supplement: Supplementary file 2 [file DataSheet2.zip › Figure3_SDSPAGE/22.06.01_13.12.13_Figure3Aright.tif]

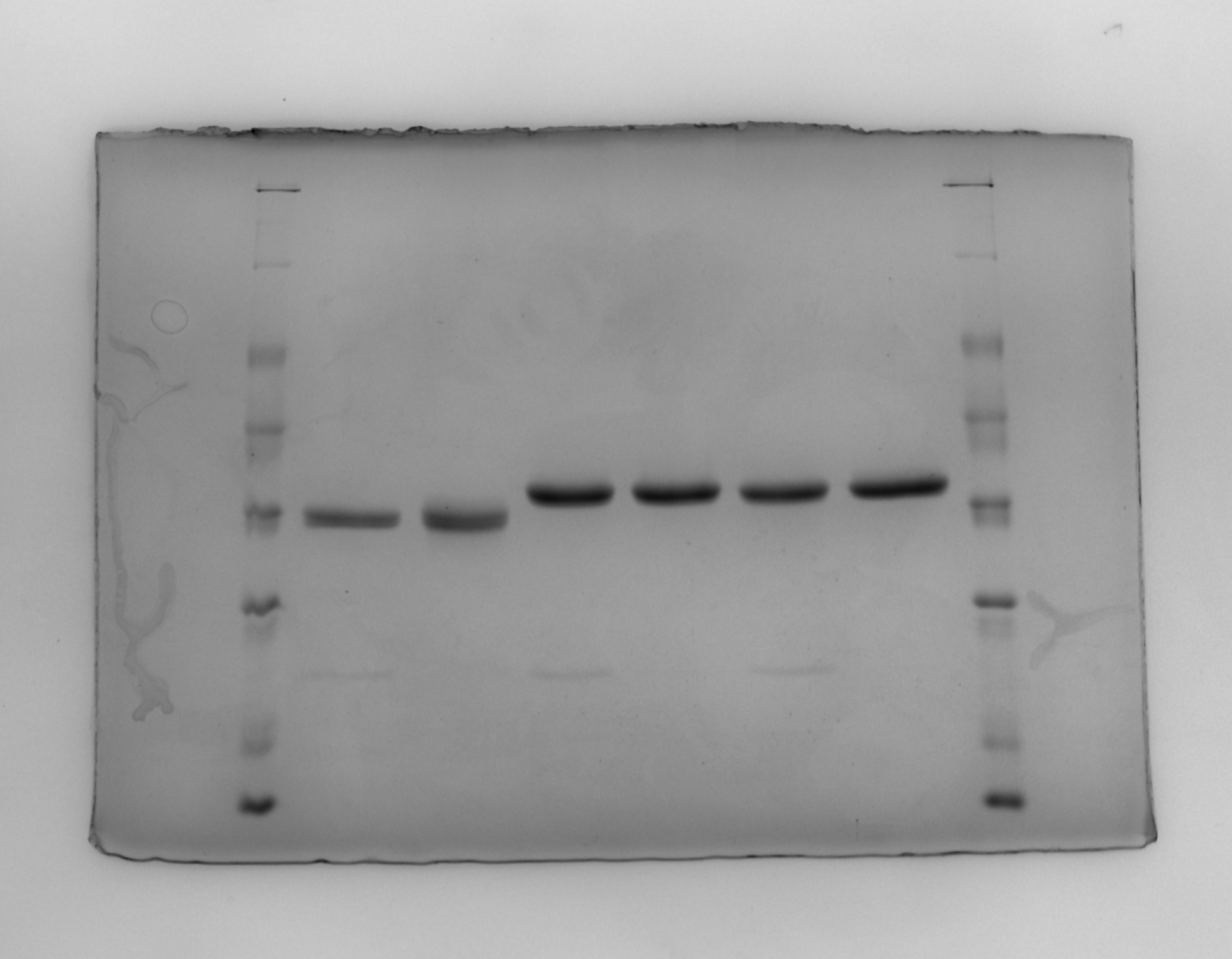

Supplement: Supplementary file 2 [file DataSheet2.zip › Figure3_SDSPAGE/23.04.14_08.53.25_Figure3Aleft.tif]

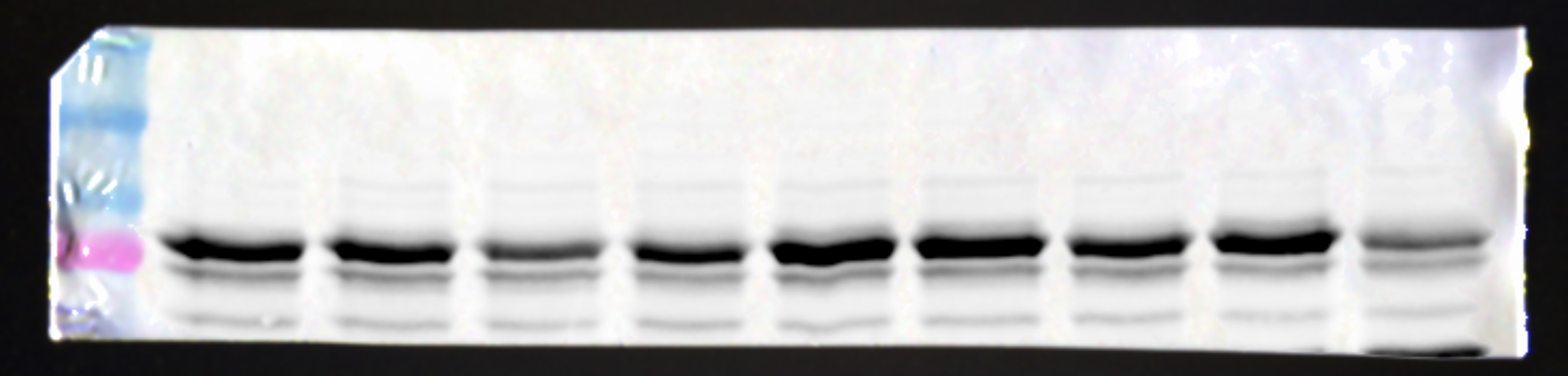

Supplement: Supplementary file 2 [file DataSheet2.zip › Figure6_WB/pSTAT3/23.04.12_08.34.31_ECL+Marker.tif]

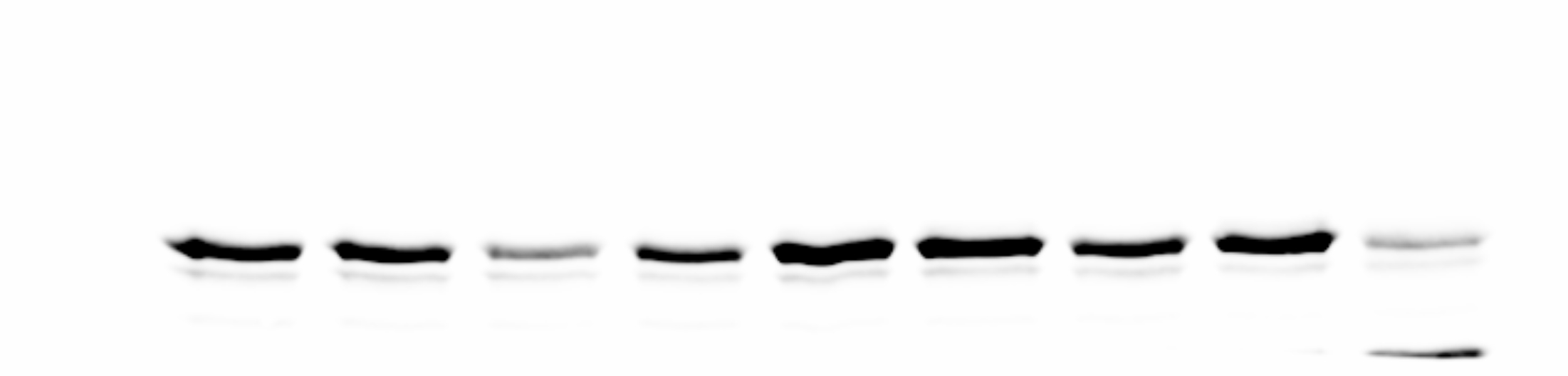

Supplement: Supplementary file 2 [file DataSheet2.zip › Figure6_WB/pSTAT3/23.04.12_08.34.31_ECL.tif]

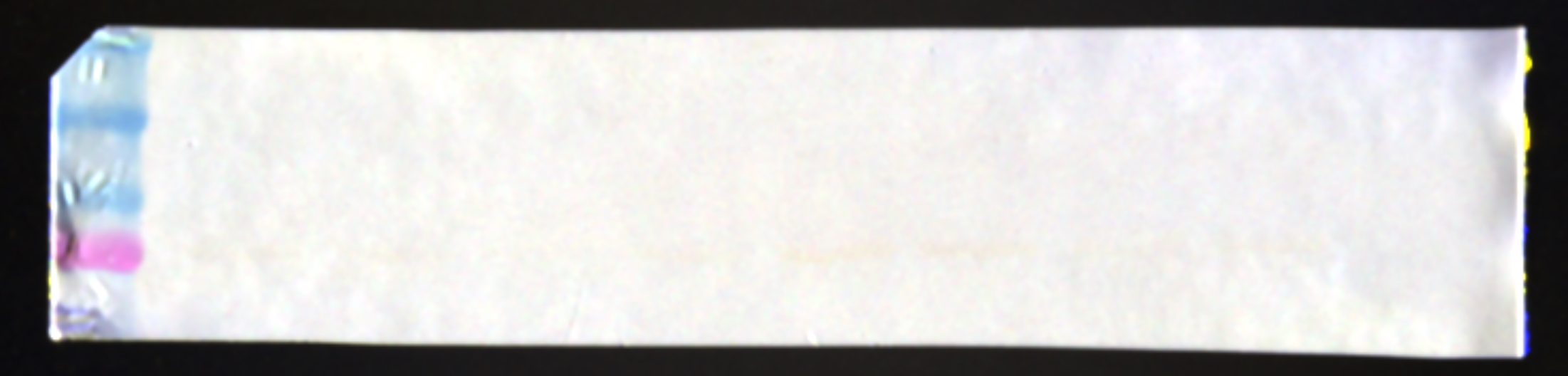

Supplement: Supplementary file 2 [file DataSheet2.zip › Figure6_WB/pSTAT3/23.04.12_08.34.31_marker.tif]

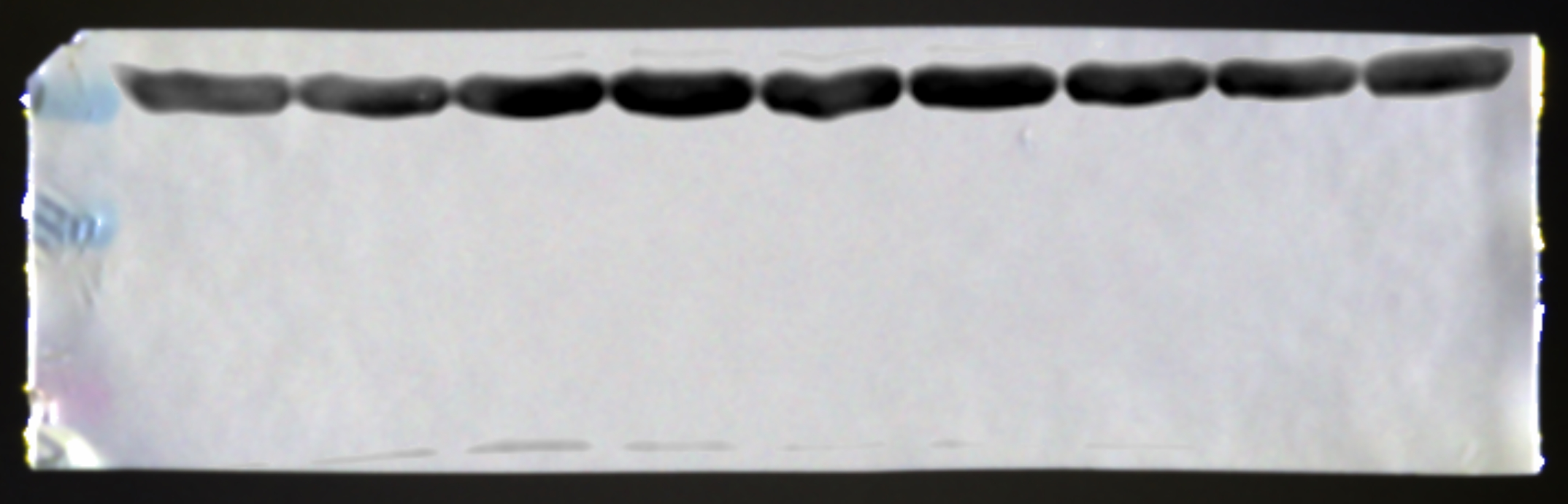

Supplement: Supplementary file 2 [file DataSheet2.zip › Figure6_WB/Tubulin/23.04.12_08.05.02_ECL+Marker.tif]

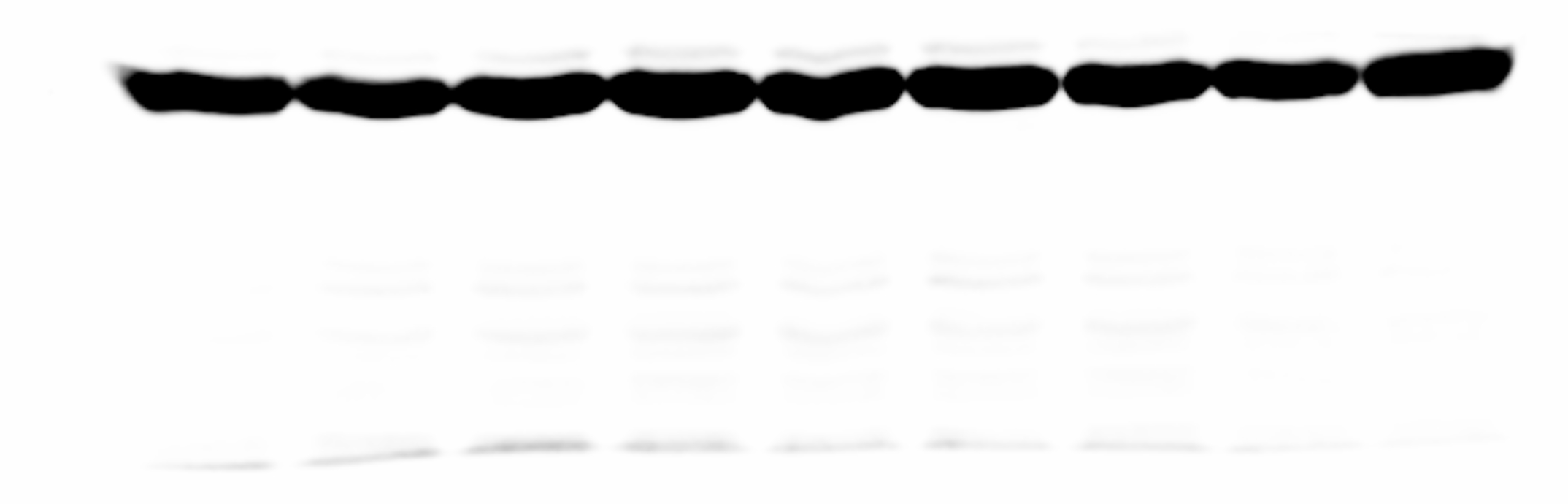

Supplement: Supplementary file 2 [file DataSheet2.zip › Figure6_WB/Tubulin/23.04.12_08.05.02_ECL.tif]

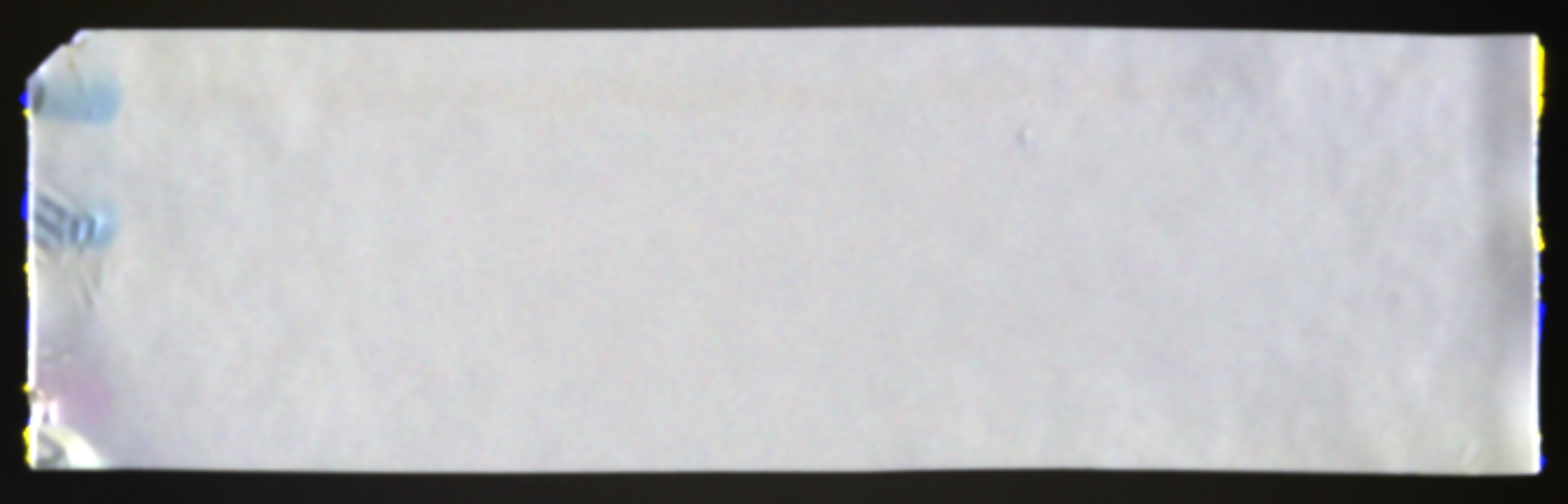

Supplement: Supplementary file 2 [file DataSheet2.zip › Figure6_WB/Tubulin/23.04.12_08.05.02_marker.tif]

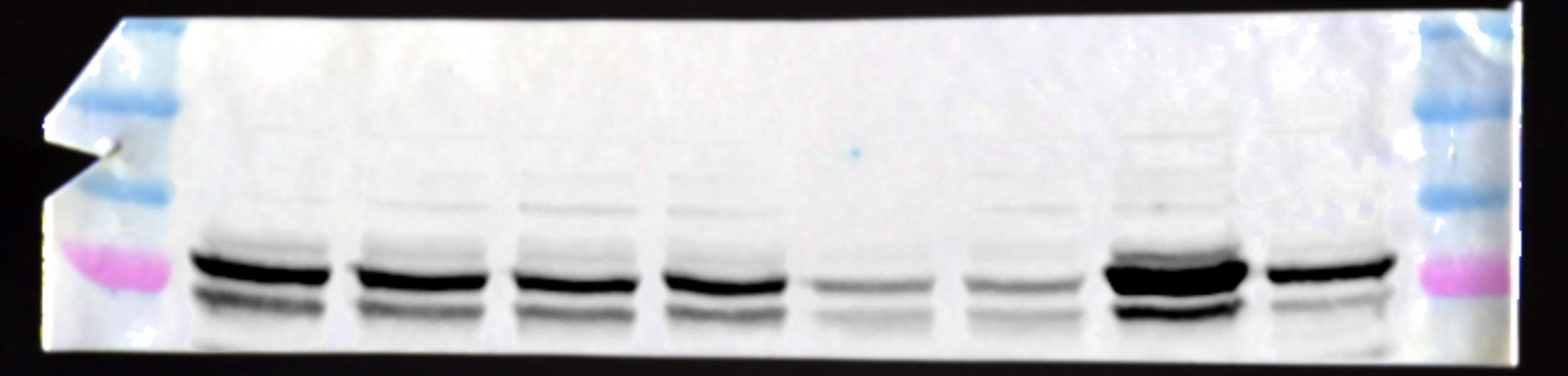

Supplement: Supplementary file 2 [file DataSheet2.zip › FigureS11_WB/FigureS9A/pSTAT3/23.04.18_08.47.22_ECL+Marker.tif]

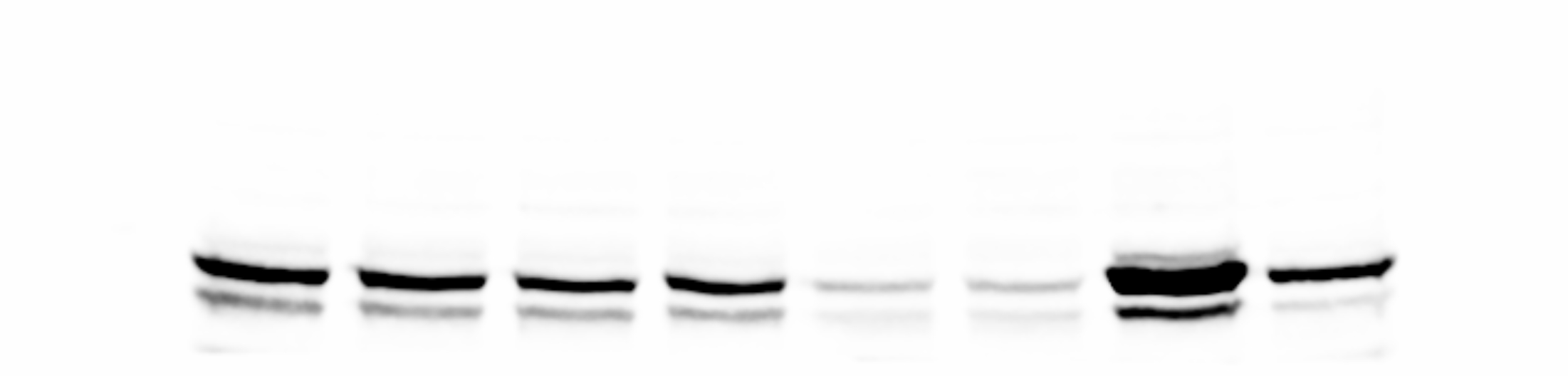

Supplement: Supplementary file 2 [file DataSheet2.zip › FigureS11_WB/FigureS9A/pSTAT3/23.04.18_08.47.22_ECL.tif]

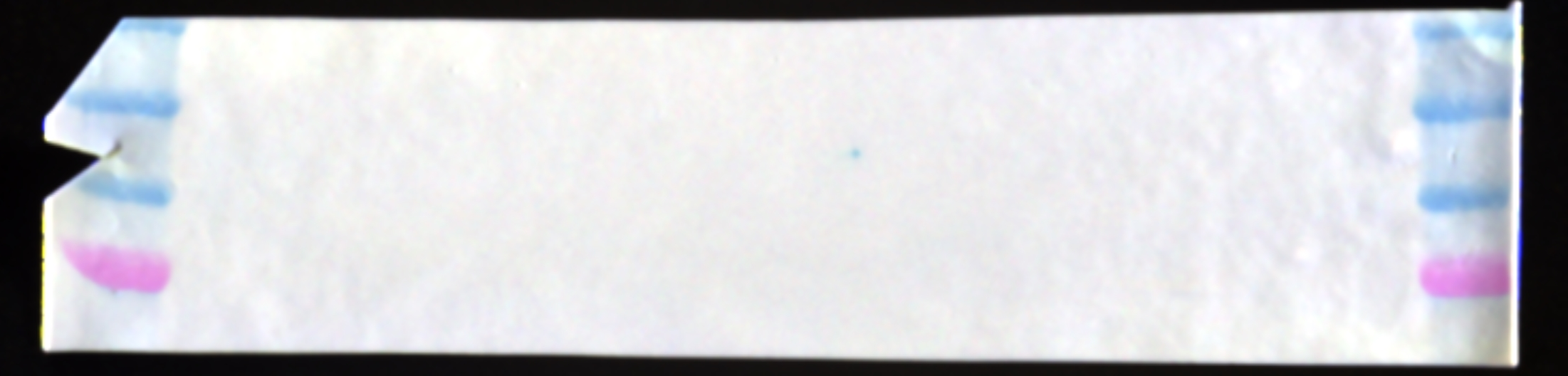

Supplement: Supplementary file 2 [file DataSheet2.zip › FigureS11_WB/FigureS9A/pSTAT3/23.04.18_08.47.22_marker.tif]

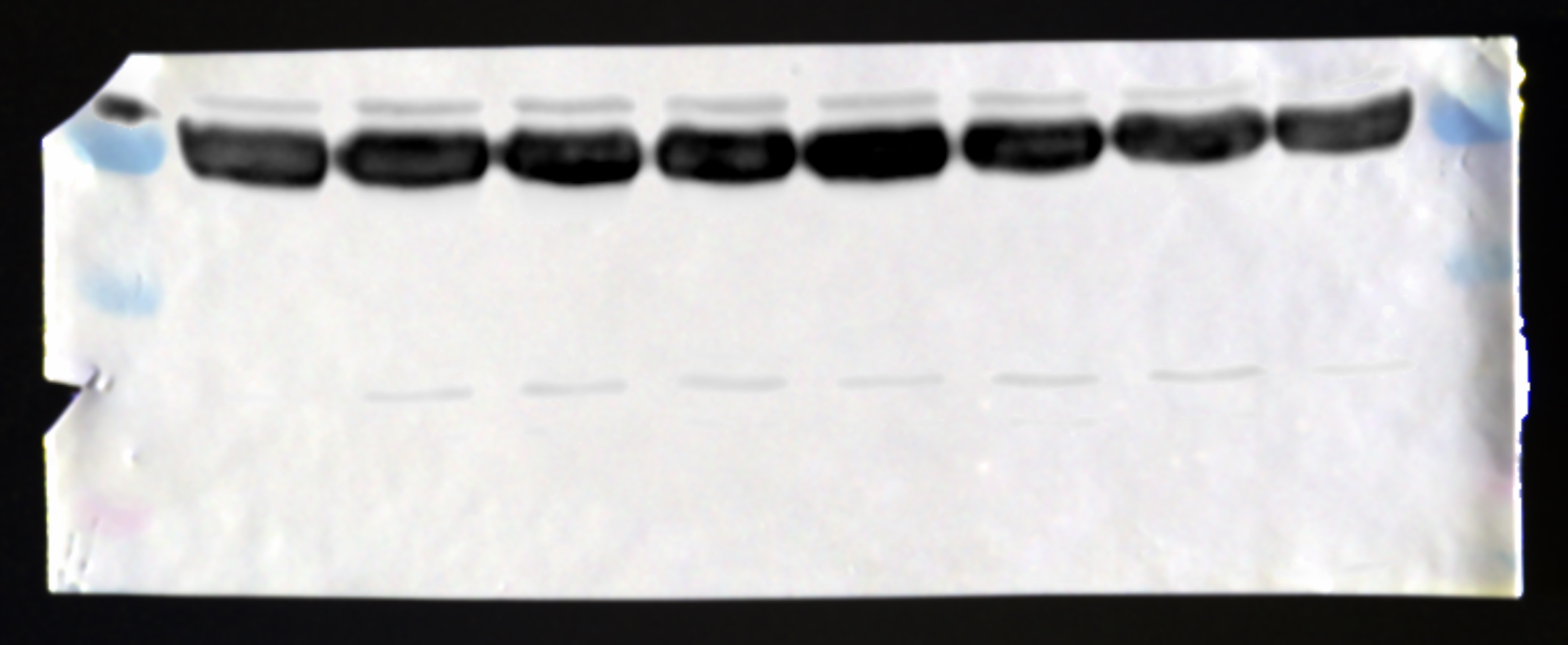

Supplement: Supplementary file 2 [file DataSheet2.zip › FigureS11_WB/FigureS9A/Tubulin/23.04.18_08.07.27_ECL+Markerc.tif]

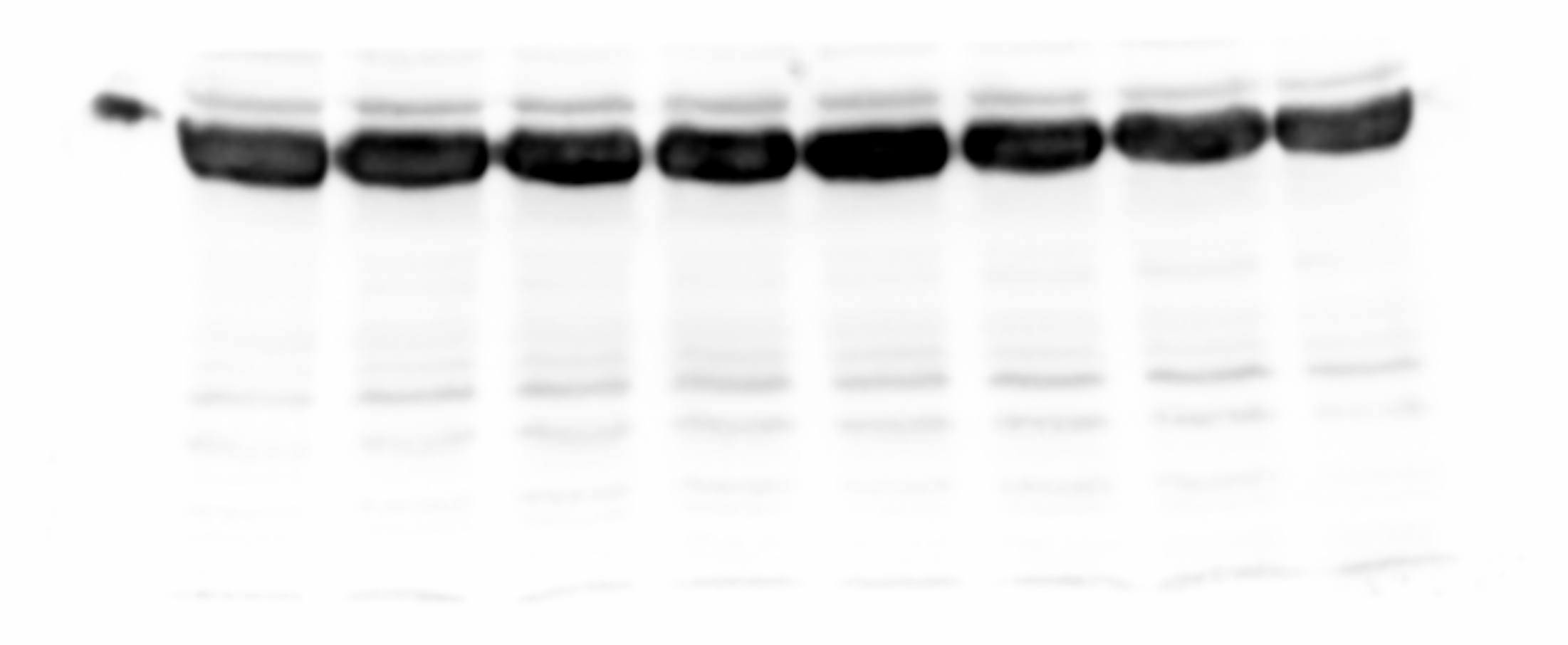

Supplement: Supplementary file 2 [file DataSheet2.zip › FigureS11_WB/FigureS9A/Tubulin/23.04.18_08.07.27_ECL.tif]

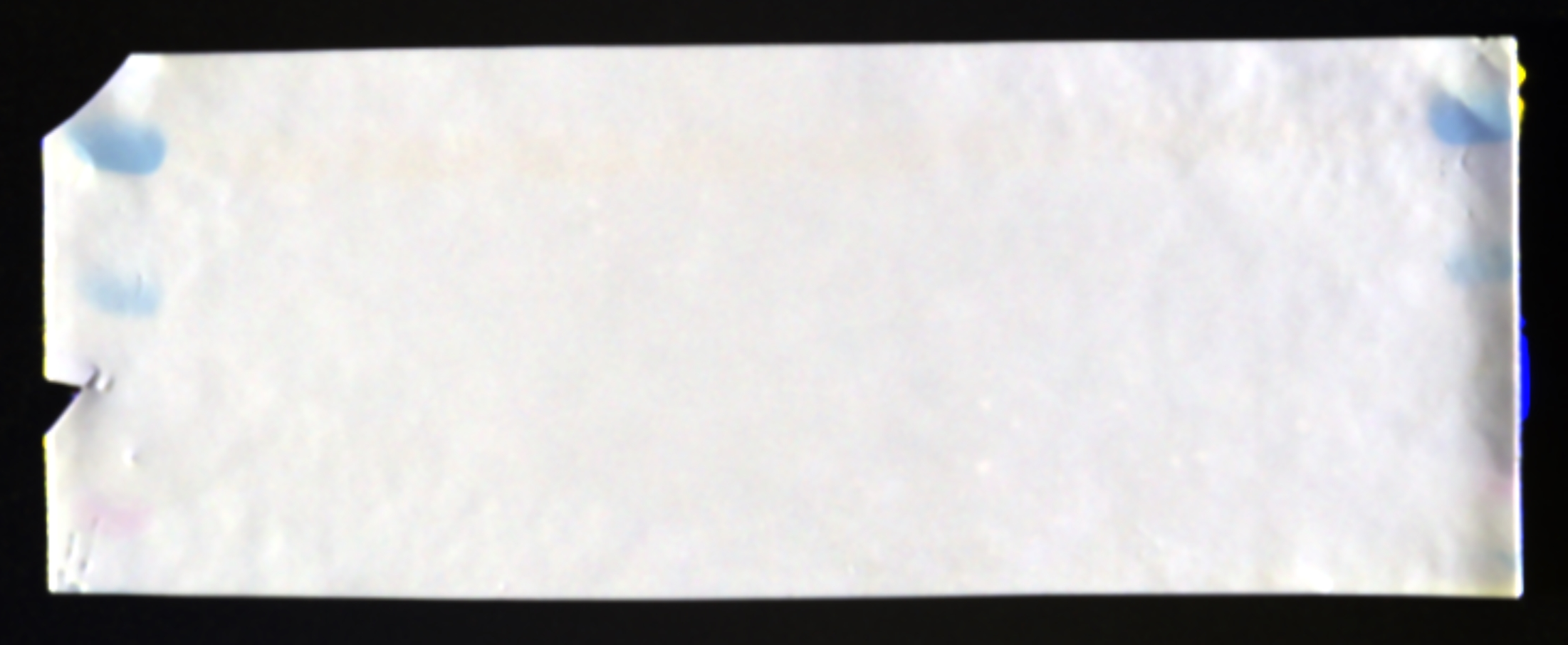

Supplement: Supplementary file 2 [file DataSheet2.zip › FigureS11_WB/FigureS9A/Tubulin/23.04.18_08.07.27_marker.tif]

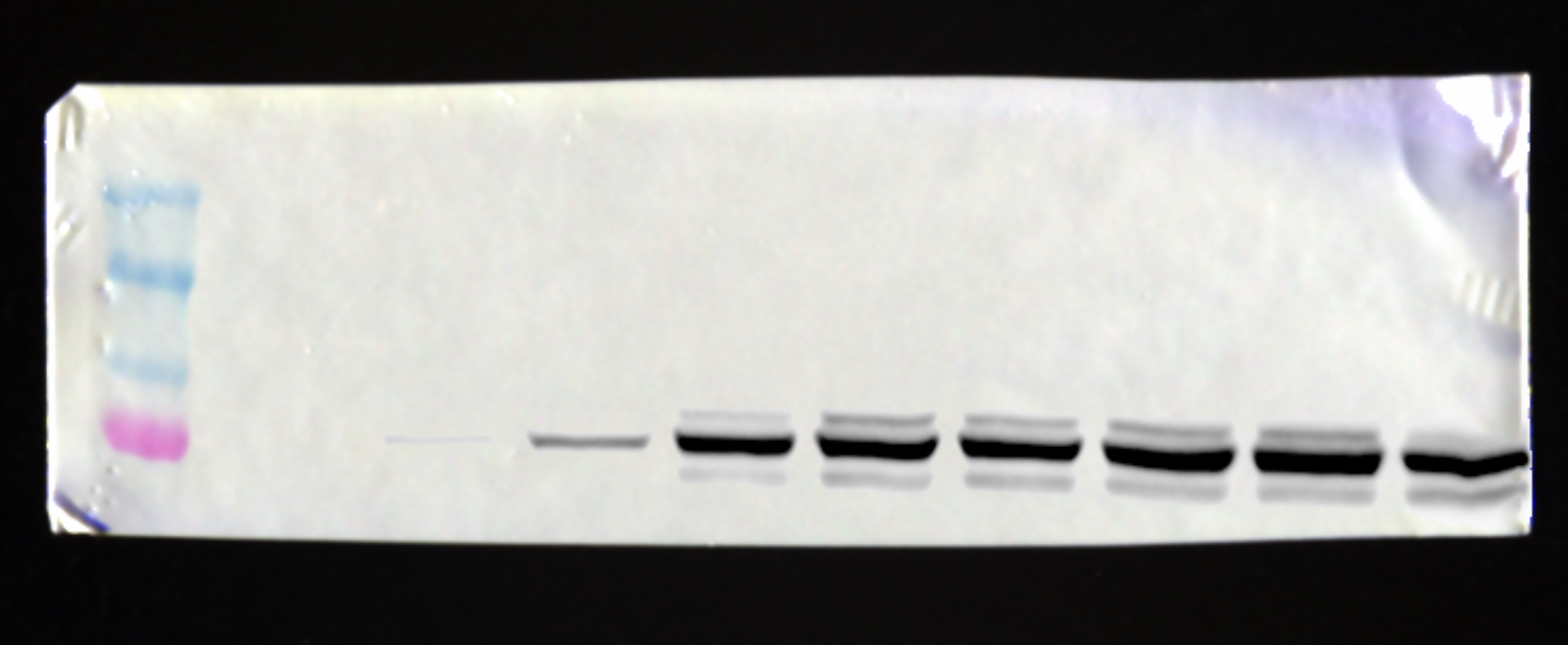

Supplement: Supplementary file 2 [file DataSheet2.zip › FigureS11_WB/FigureS9B/pSTAT3/23.04.04_08.40.36_ECL+Marker.tif]

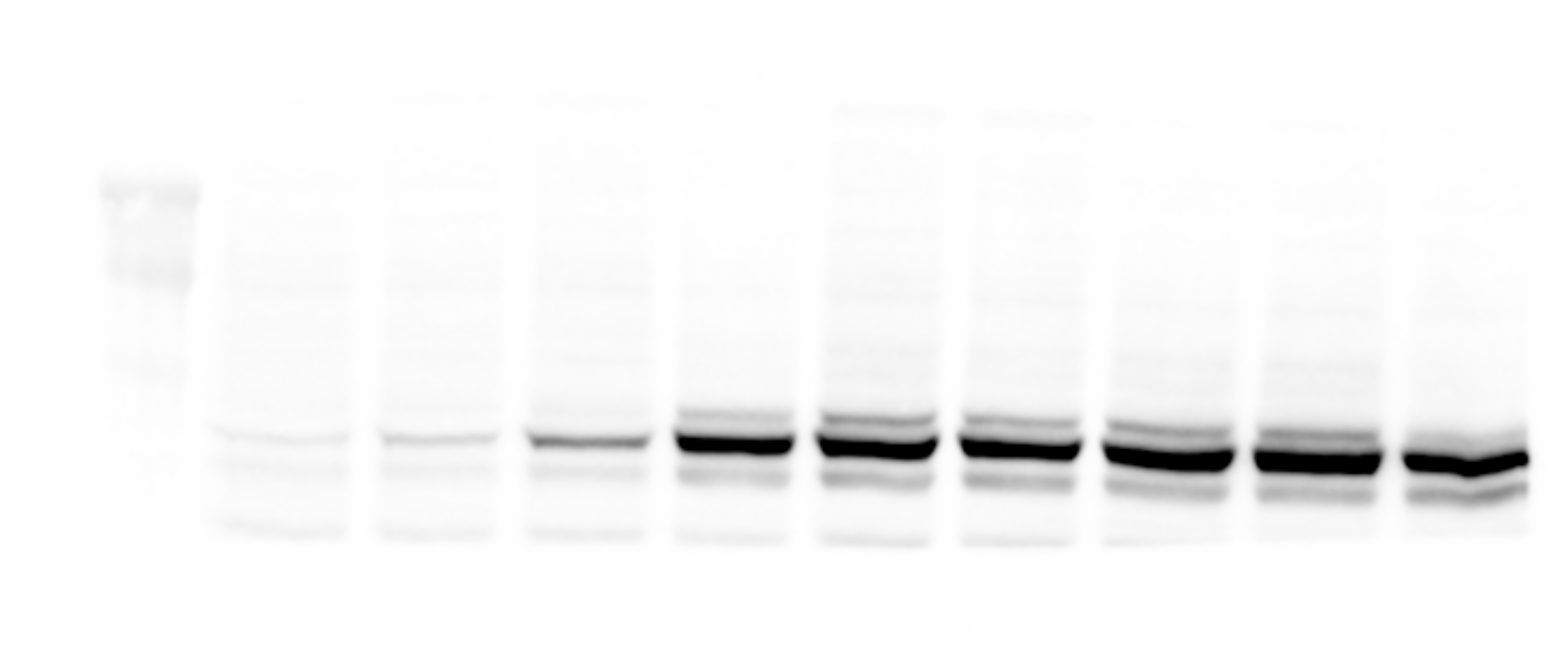

Supplement: Supplementary file 2 [file DataSheet2.zip › FigureS11_WB/FigureS9B/pSTAT3/23.04.04_08.40.36_ECL.tif]

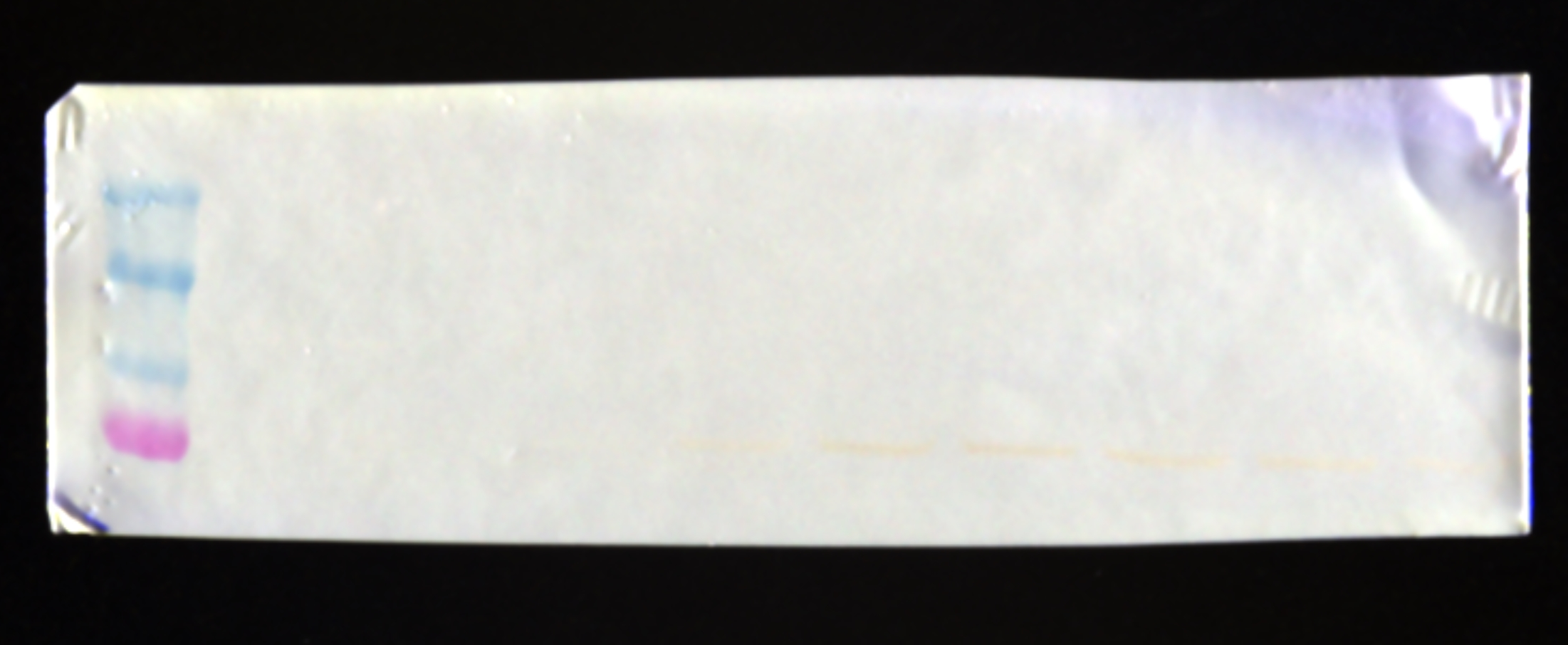

Supplement: Supplementary file 2 [file DataSheet2.zip › FigureS11_WB/FigureS9B/pSTAT3/23.04.04_08.40.36_marker.tif]

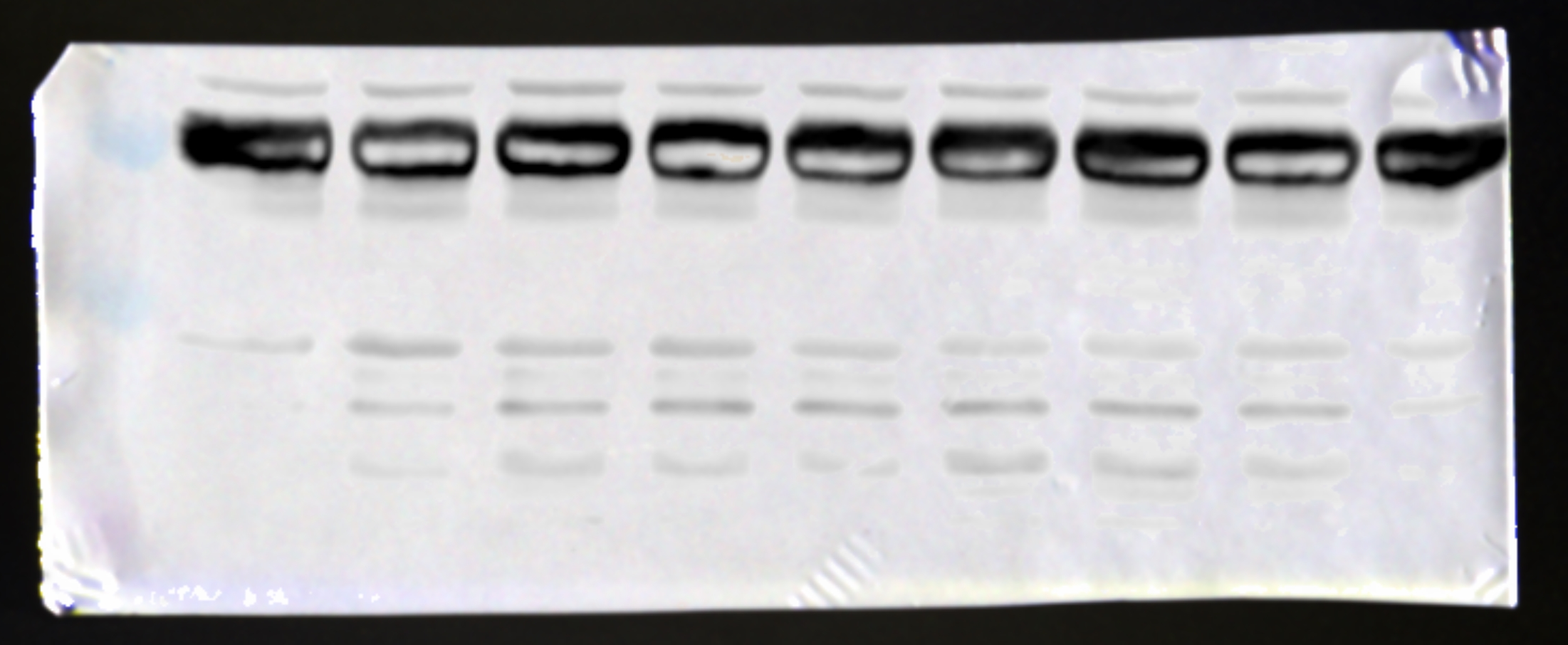

Supplement: Supplementary file 2 [file DataSheet2.zip › FigureS11_WB/FigureS9B/Tubulin/23.04.04_09.03.49_ECL+Markerc.tif]

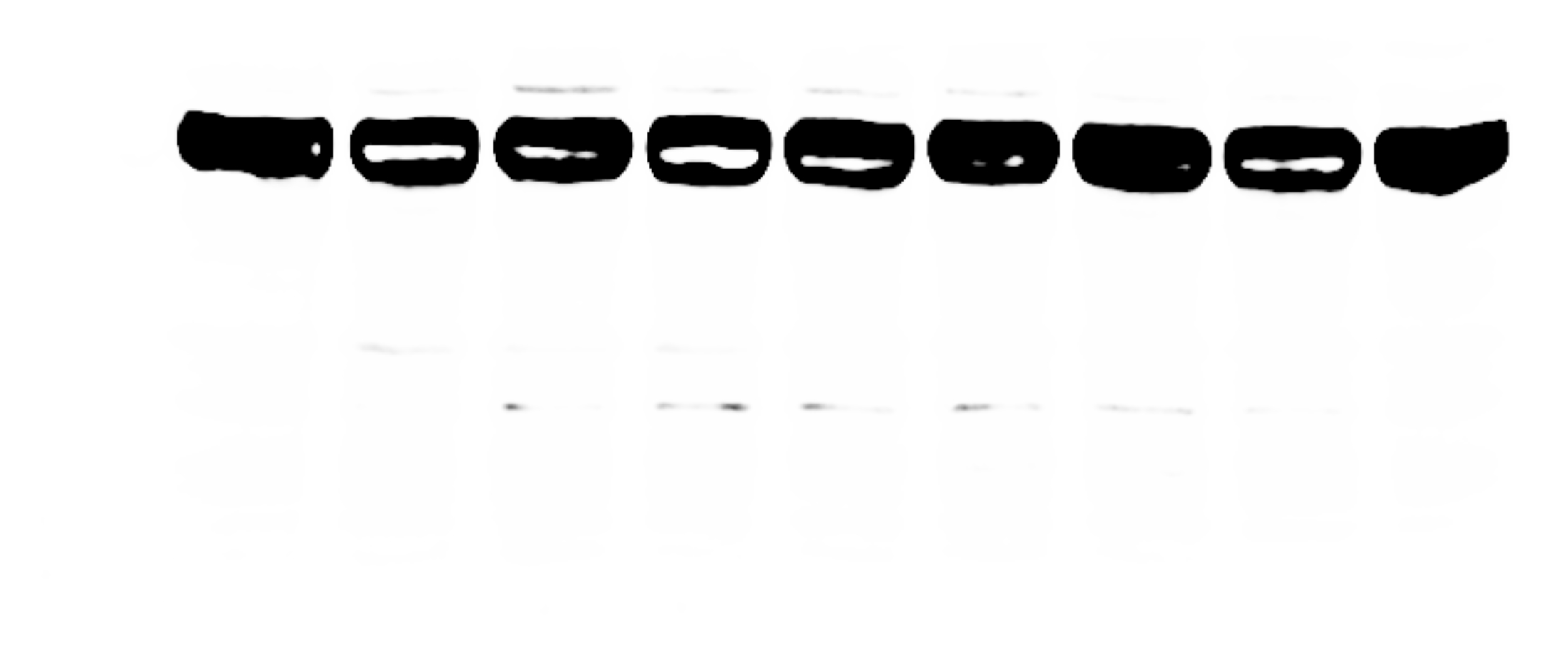

Supplement: Supplementary file 2 [file DataSheet2.zip › FigureS11_WB/FigureS9B/Tubulin/23.04.04_09.03.49_ECL.tif]

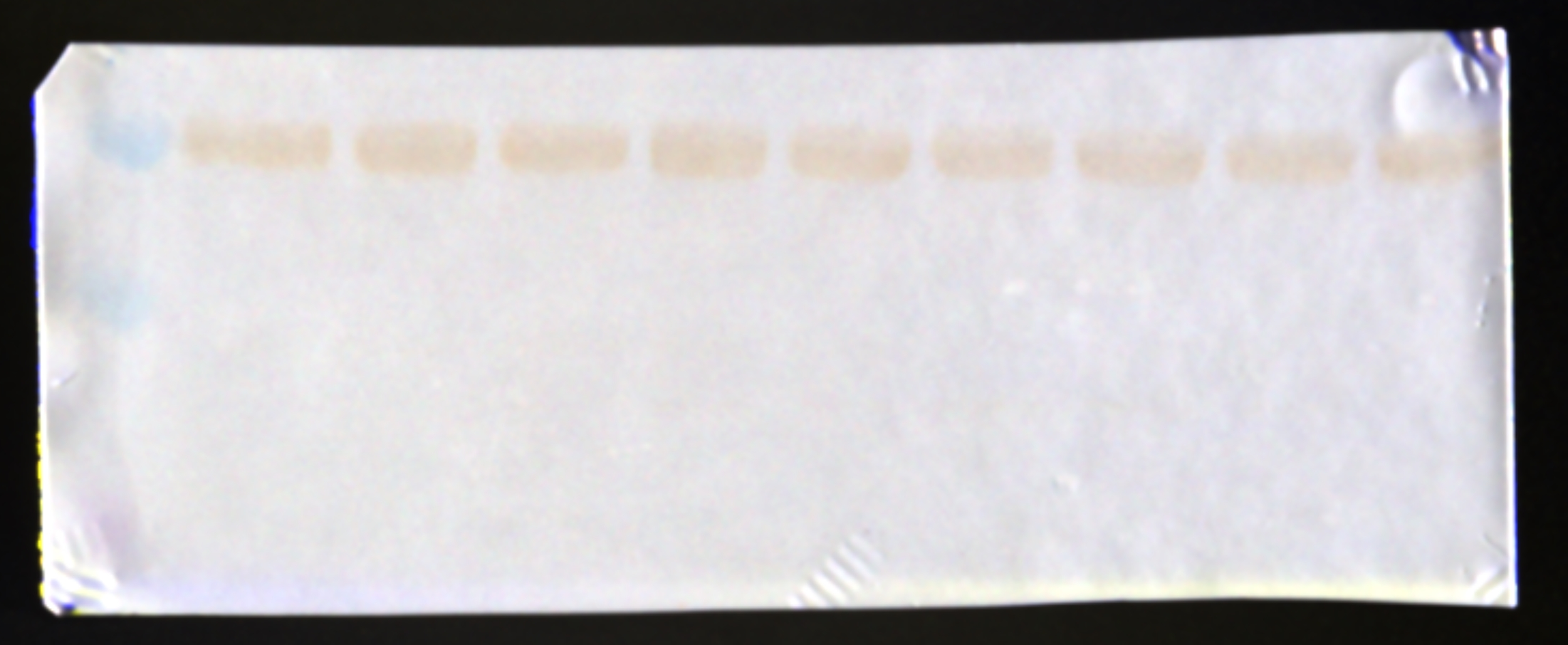

Supplement: Supplementary file 2 [file DataSheet2.zip › FigureS11_WB/FigureS9B/Tubulin/23.04.04_09.03.49_marker.tif]

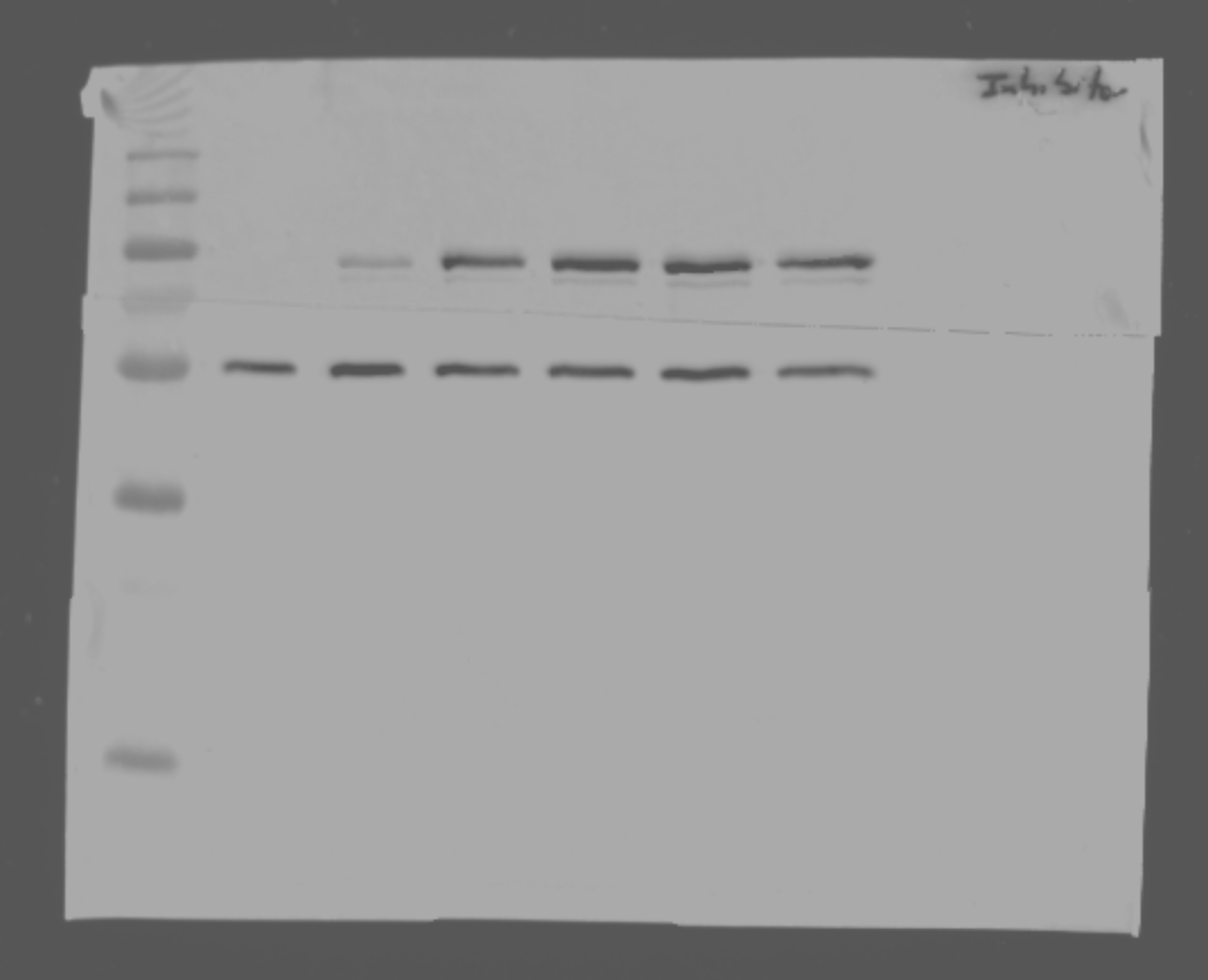

Supplement: Supplementary file 2 [file DataSheet2.zip › FigureS11_WB/FigureS9C/pSTAT3(down)+Tubulin(Top)/IL20R2DC_ECL+Marker.tif]

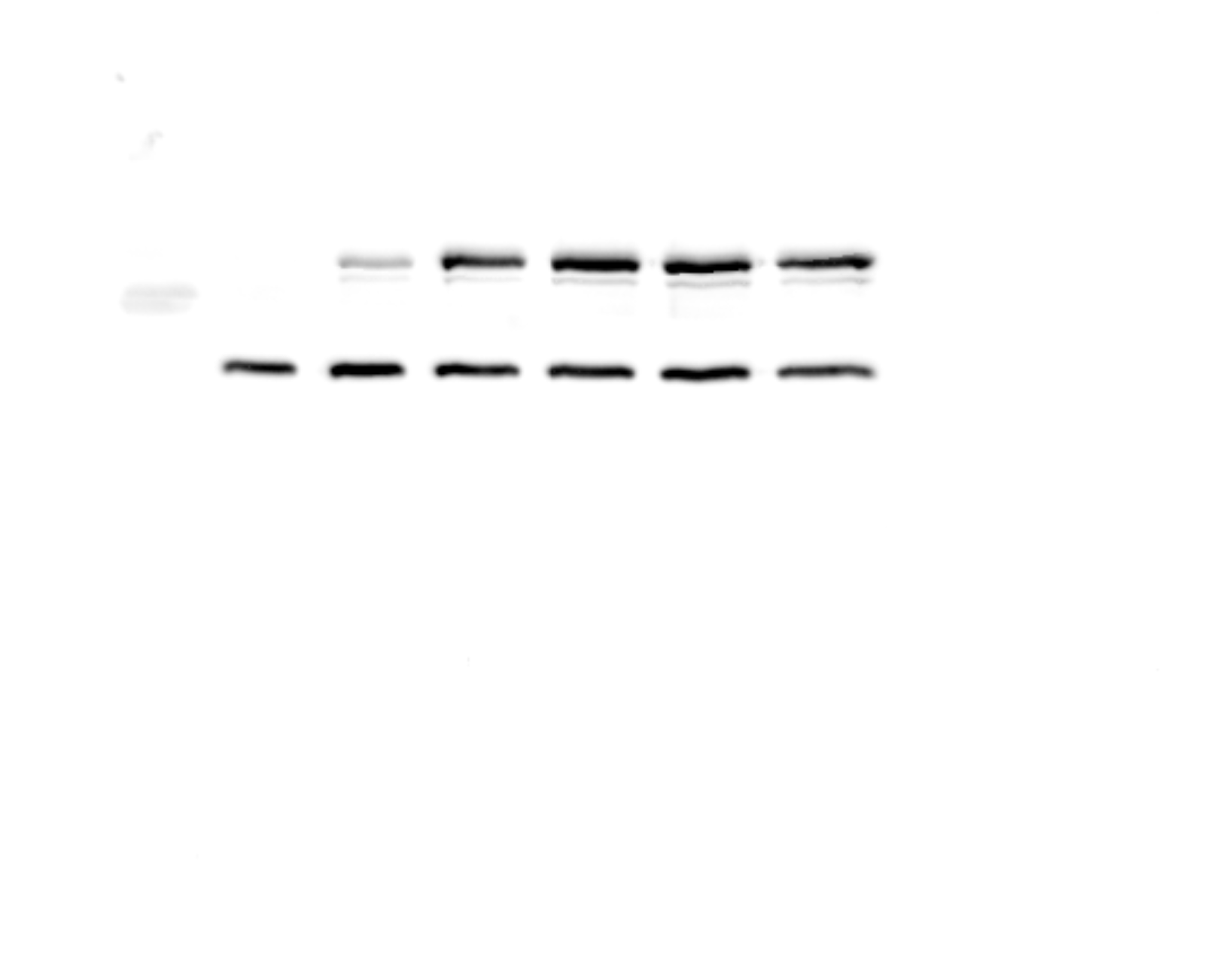

Supplement: Supplementary file 2 [file DataSheet2.zip › FigureS11_WB/FigureS9C/pSTAT3(down)+Tubulin(Top)/IL20R2DC_ECL.tif]

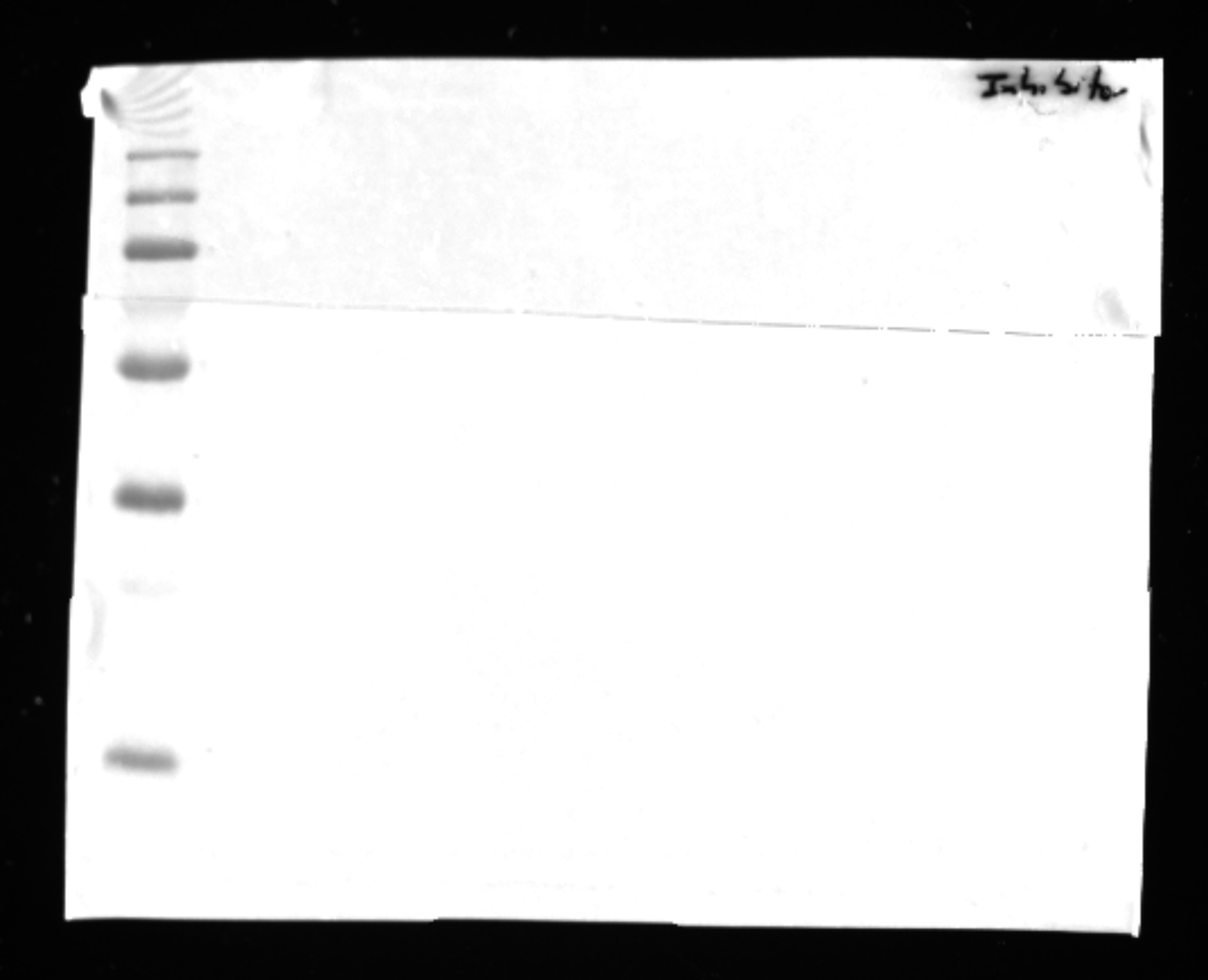

Supplement: Supplementary file 2 [file DataSheet2.zip › FigureS11_WB/FigureS9C/pSTAT3(down)+Tubulin(Top)/IL20R2DC_marker.tif]

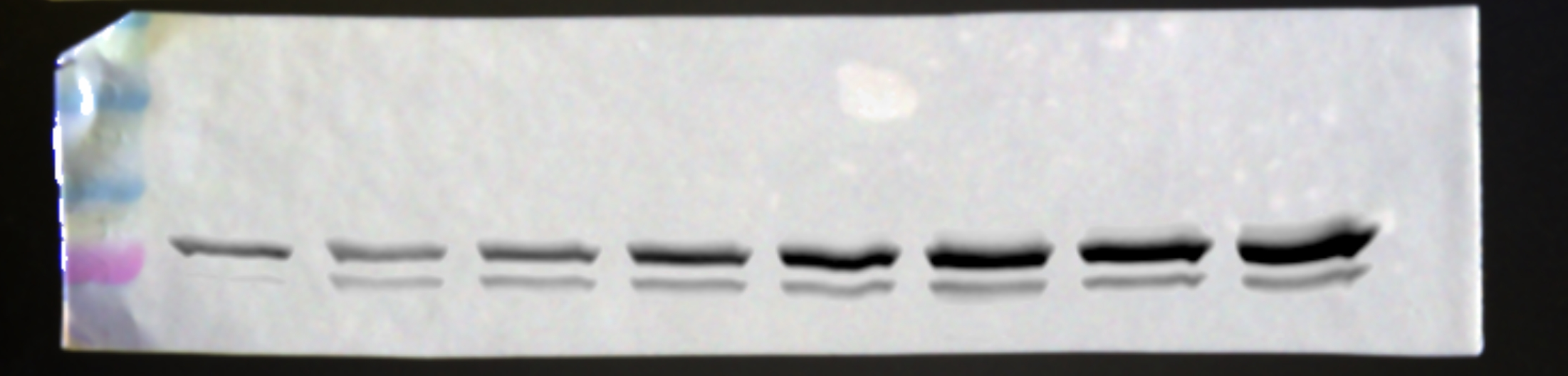

Supplement: Supplementary file 2 [file DataSheet2.zip › FigureS12_WB/FigureS11A/pSTAT3/IL24B4(TOP)/23.04.18_08.57.53_ECL+Marker.tif]

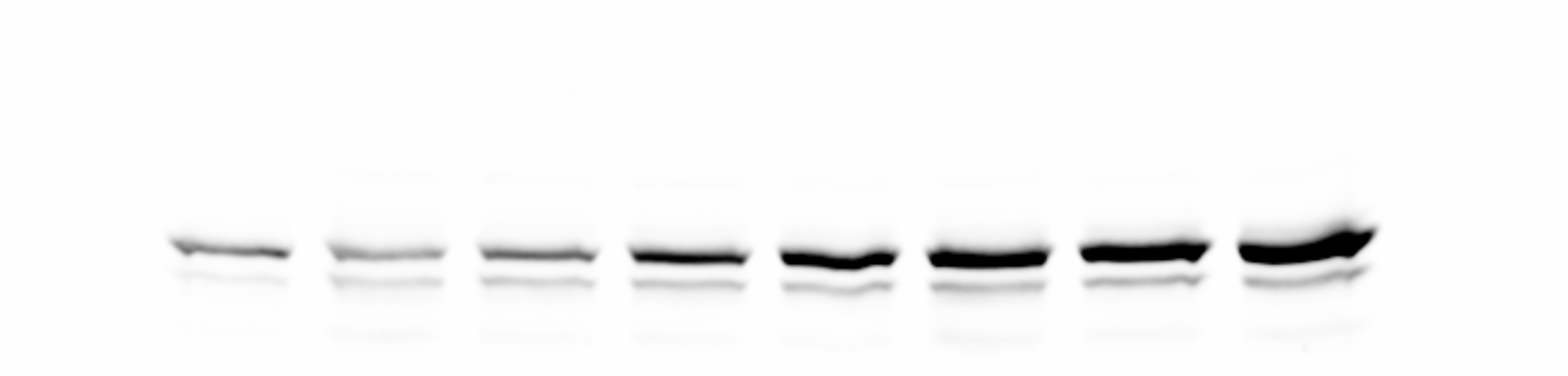

Supplement: Supplementary file 2 [file DataSheet2.zip › FigureS12_WB/FigureS11A/pSTAT3/IL24B4(TOP)/23.04.18_08.57.53_ECL.tif]

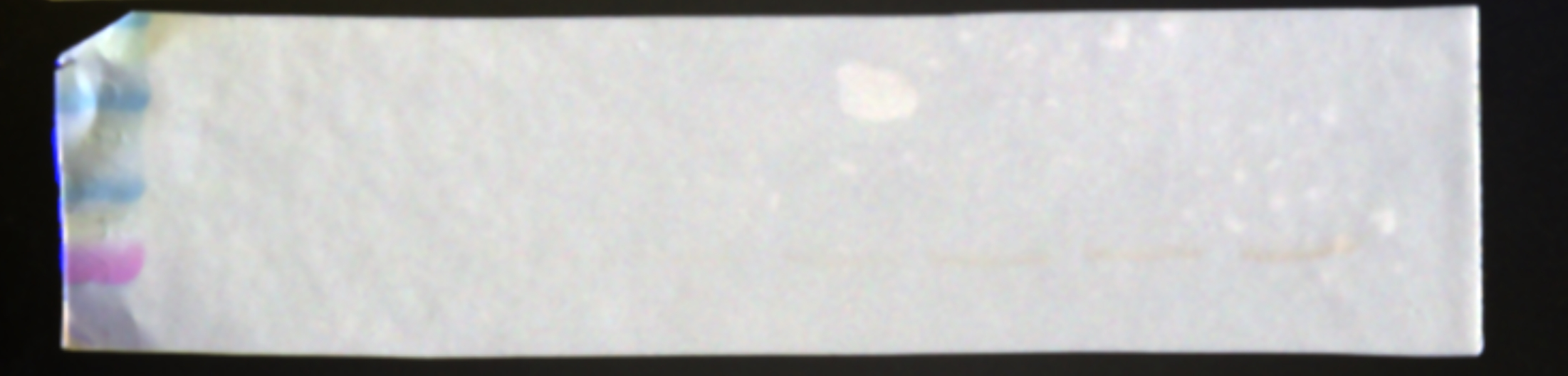

Supplement: Supplementary file 2 [file DataSheet2.zip › FigureS12_WB/FigureS11A/pSTAT3/IL24B4(TOP)/23.04.18_08.57.53_marker.tif]

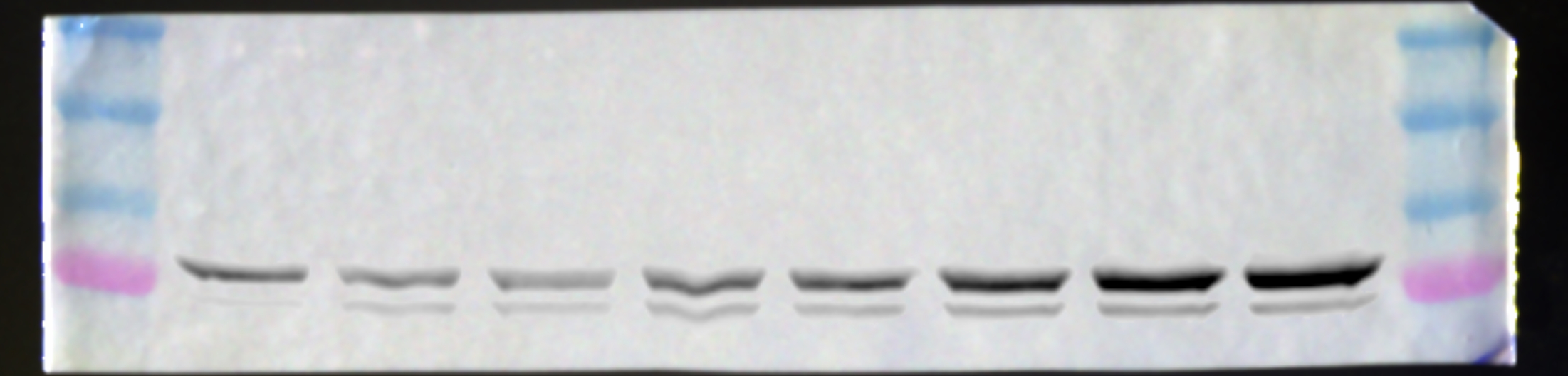

Supplement: Supplementary file 2 [file DataSheet2.zip › FigureS12_WB/FigureS11A/pSTAT3/Y204NBY(MEDIUM)/23.04.18_08.57.53_ECL+Marker.tif]

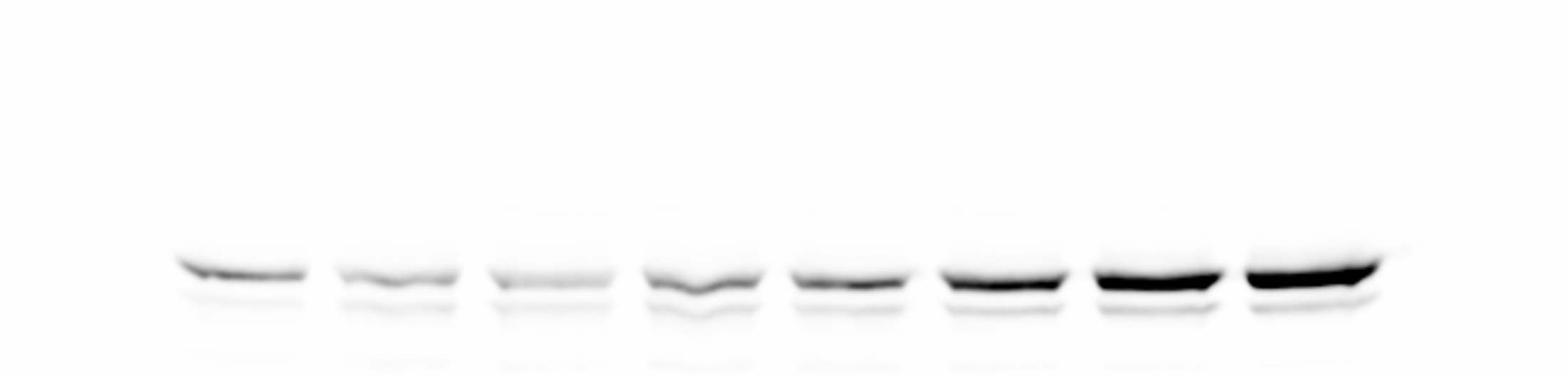

Supplement: Supplementary file 2 [file DataSheet2.zip › FigureS12_WB/FigureS11A/pSTAT3/Y204NBY(MEDIUM)/23.04.18_08.57.53_ECL.tif]

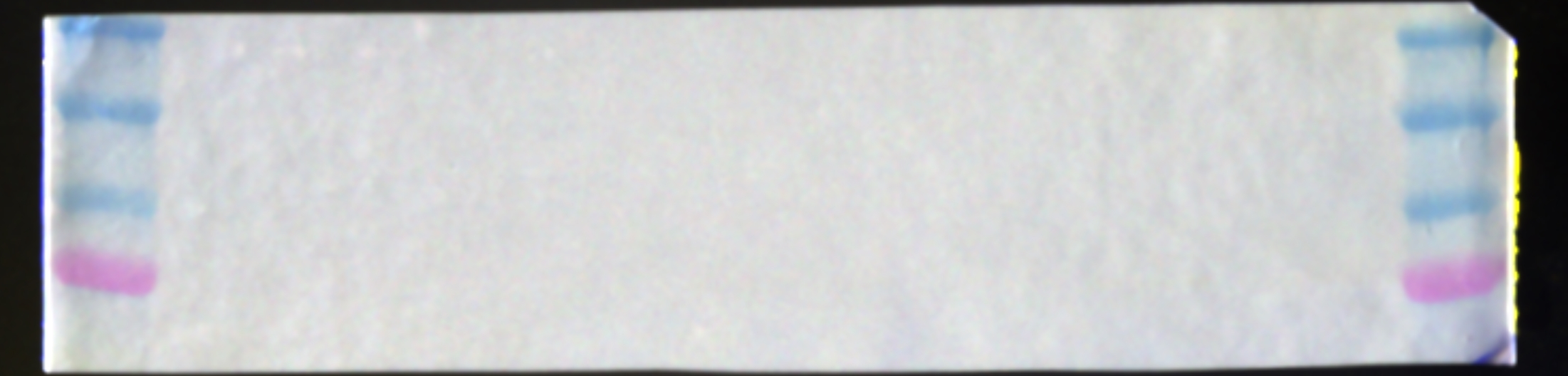

Supplement: Supplementary file 2 [file DataSheet2.zip › FigureS12_WB/FigureS11A/pSTAT3/Y204NBY(MEDIUM)/23.04.18_08.57.53_marker.tif]

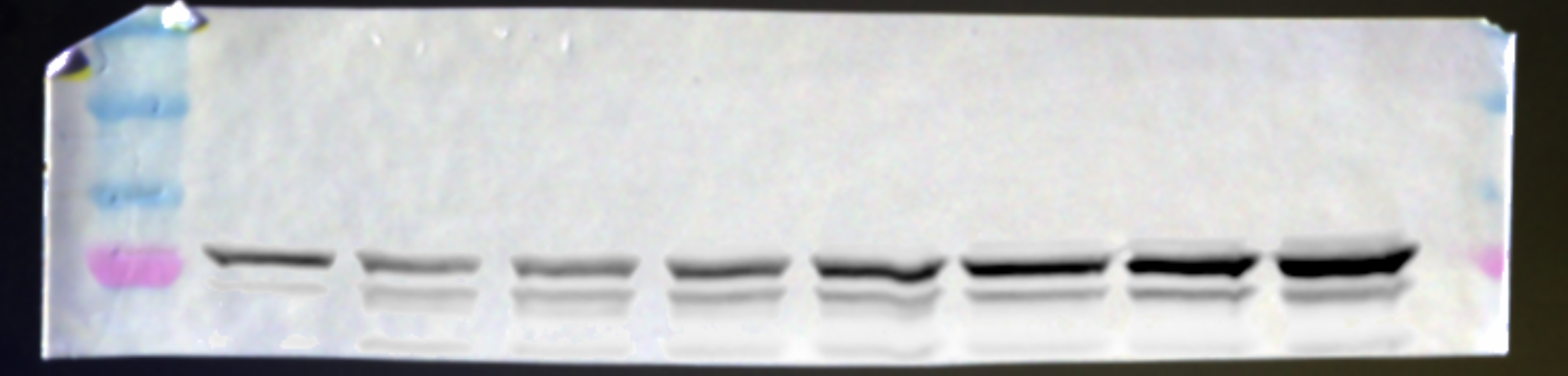

Supplement: Supplementary file 2 [file DataSheet2.zip › FigureS12_WB/FigureS11A/pSTAT3/Y204NBY+UV(BOTTOM)/23.04.18_08.57.53_ECL+Marker.tif]

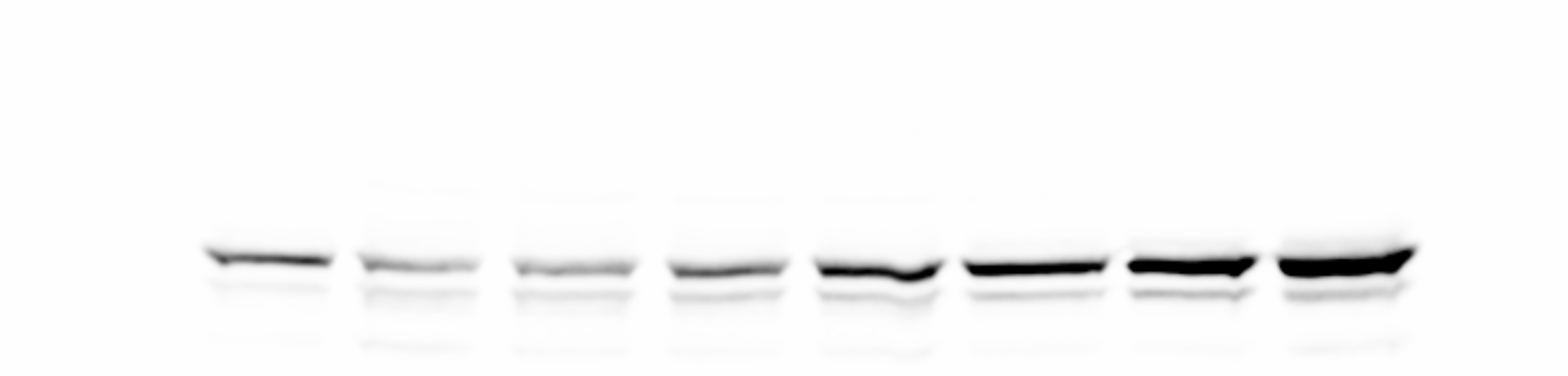

Supplement: Supplementary file 2 [file DataSheet2.zip › FigureS12_WB/FigureS11A/pSTAT3/Y204NBY+UV(BOTTOM)/23.04.18_08.57.53_ECL.tif]

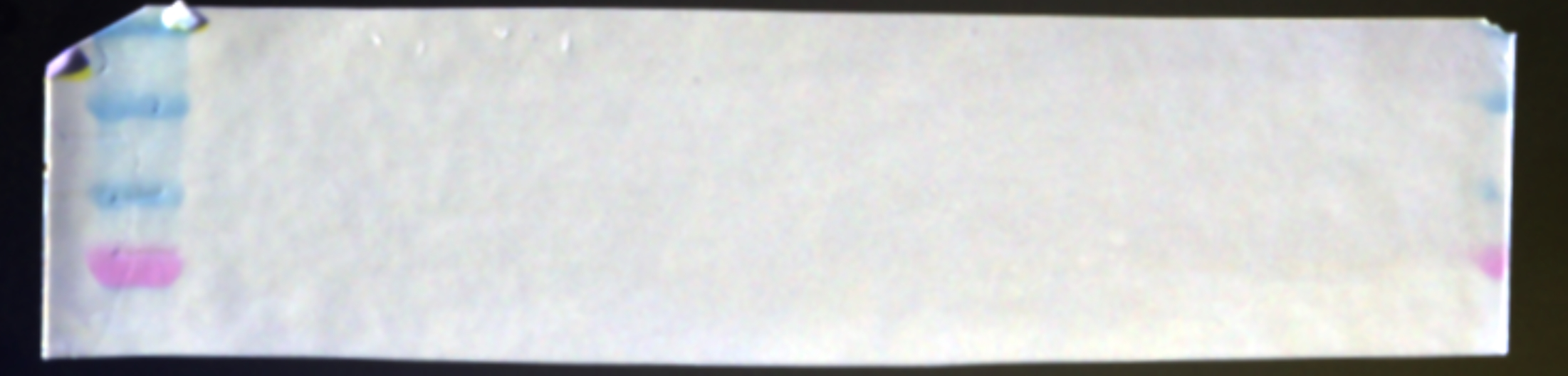

Supplement: Supplementary file 2 [file DataSheet2.zip › FigureS12_WB/FigureS11A/pSTAT3/Y204NBY+UV(BOTTOM)/23.04.18_08.57.53_marker.tif]

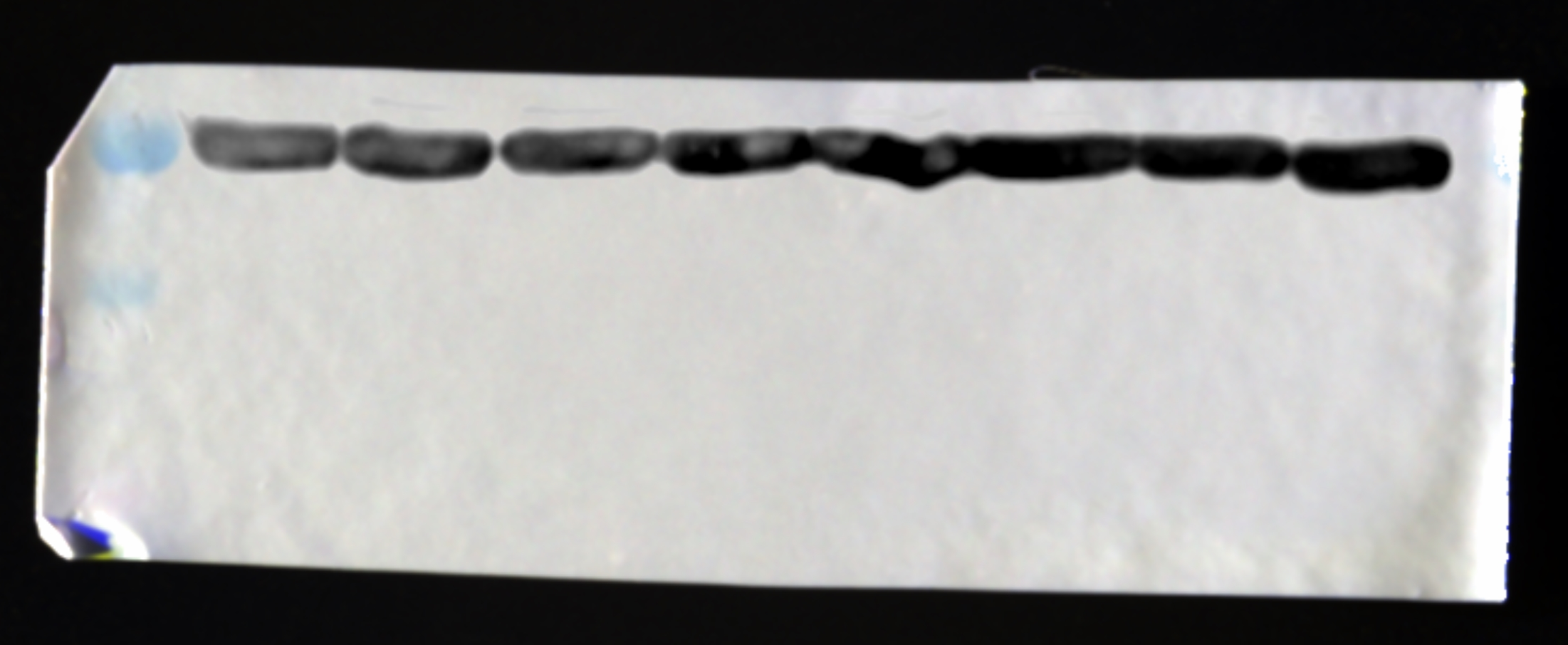

Supplement: Supplementary file 2 [file DataSheet2.zip › FigureS12_WB/FigureS11A/Tubulin/204+/23.04.18_08.03.41_ECL+Marker.tif]

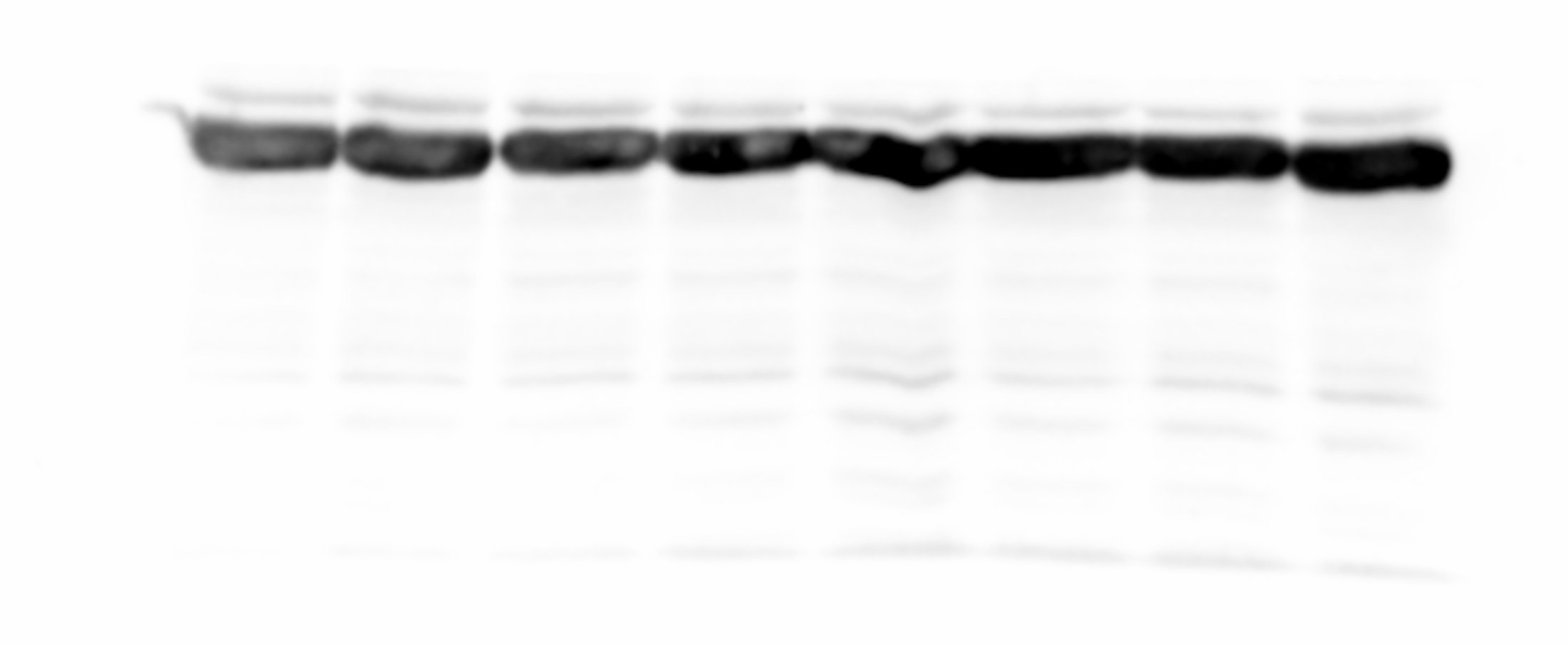

Supplement: Supplementary file 2 [file DataSheet2.zip › FigureS12_WB/FigureS11A/Tubulin/204+/23.04.18_08.03.41_ECL.tif]

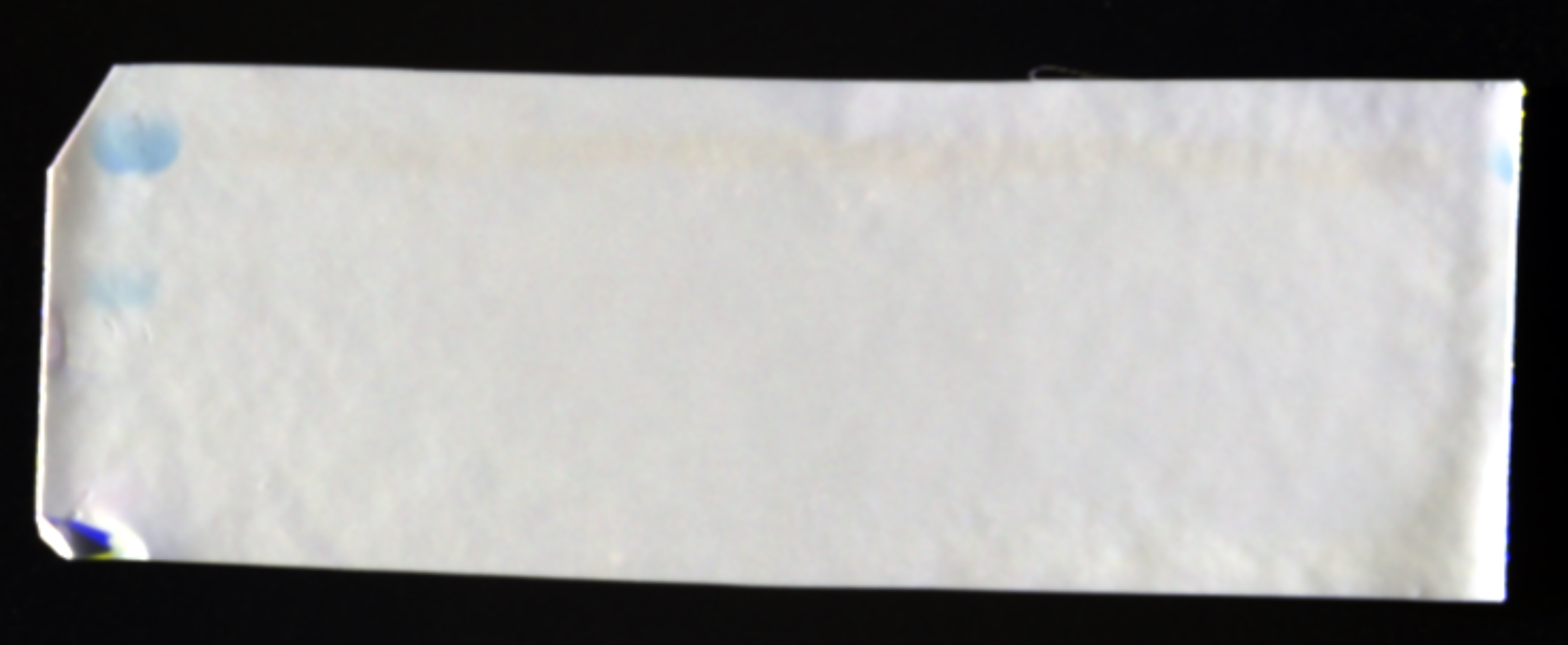

Supplement: Supplementary file 2 [file DataSheet2.zip › FigureS12_WB/FigureS11A/Tubulin/204+/23.04.18_08.03.41_marker.tif]

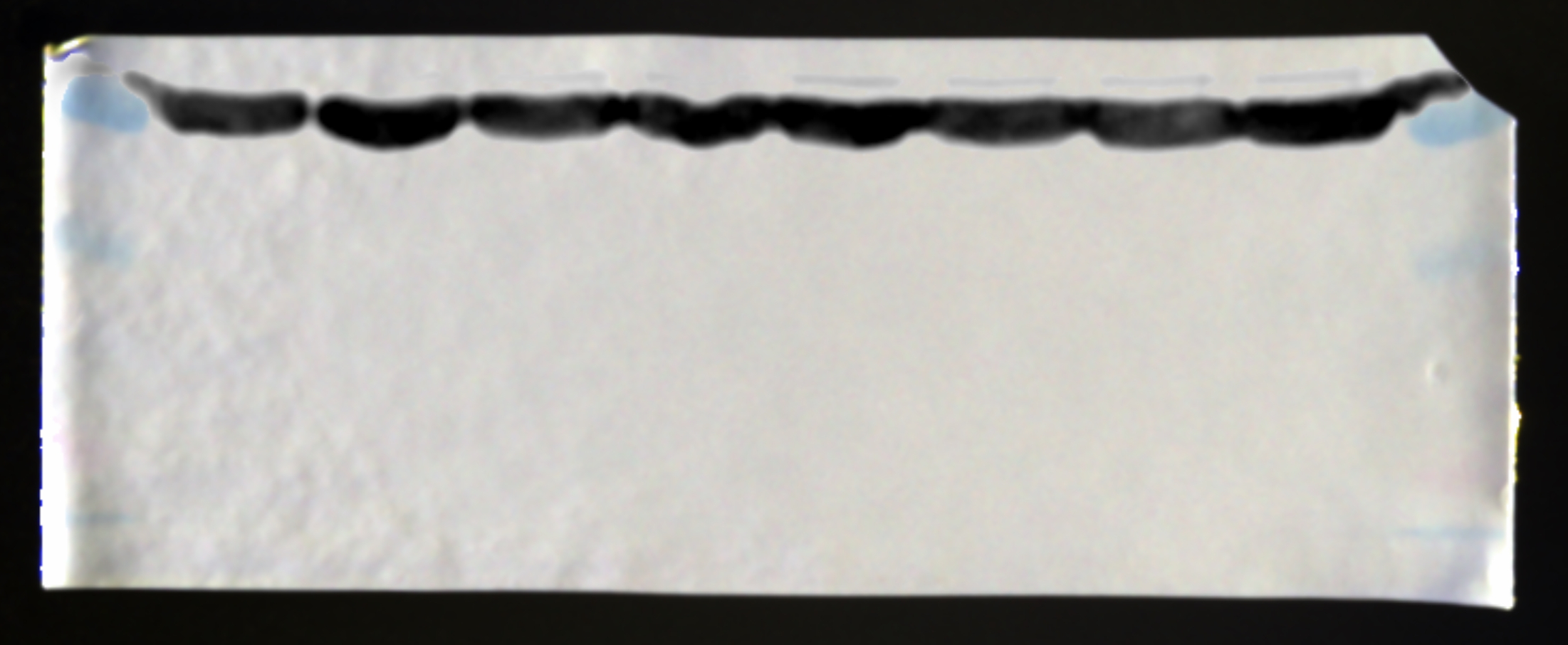

Supplement: Supplementary file 2 [file DataSheet2.zip › FigureS12_WB/FigureS11A/Tubulin/204-/23.04.18_08.01.14_ECL+Marker.tif]

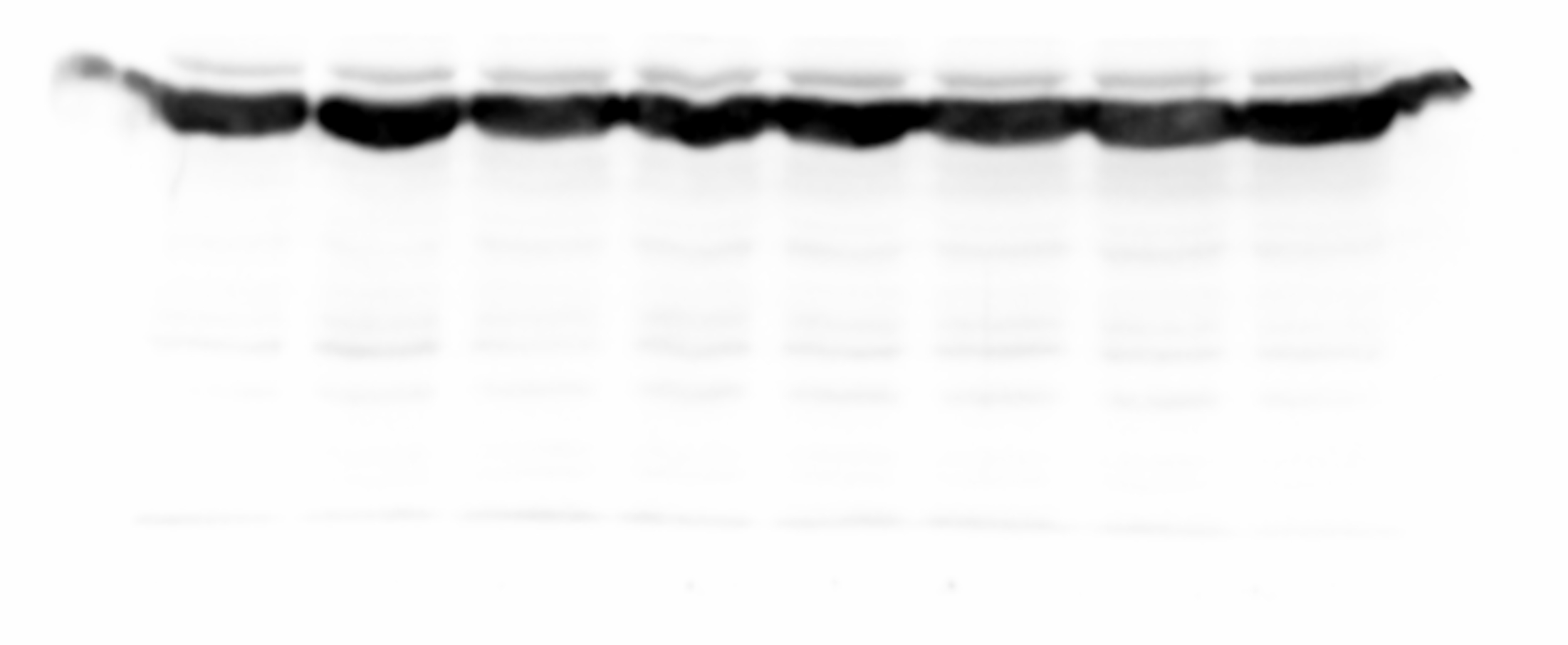

Supplement: Supplementary file 2 [file DataSheet2.zip › FigureS12_WB/FigureS11A/Tubulin/204-/23.04.18_08.01.14_ECL.tif]

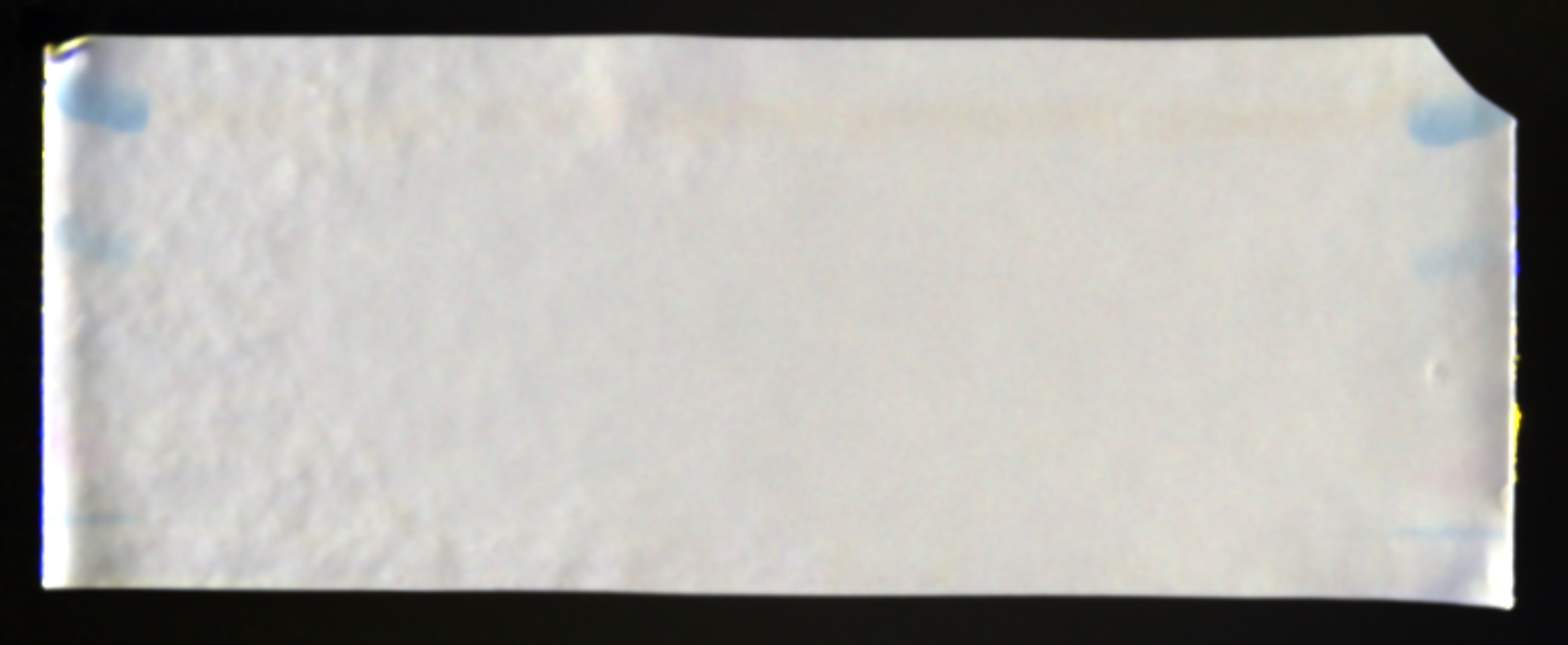

Supplement: Supplementary file 2 [file DataSheet2.zip › FigureS12_WB/FigureS11A/Tubulin/204-/23.04.18_08.01.14_marker.tif]

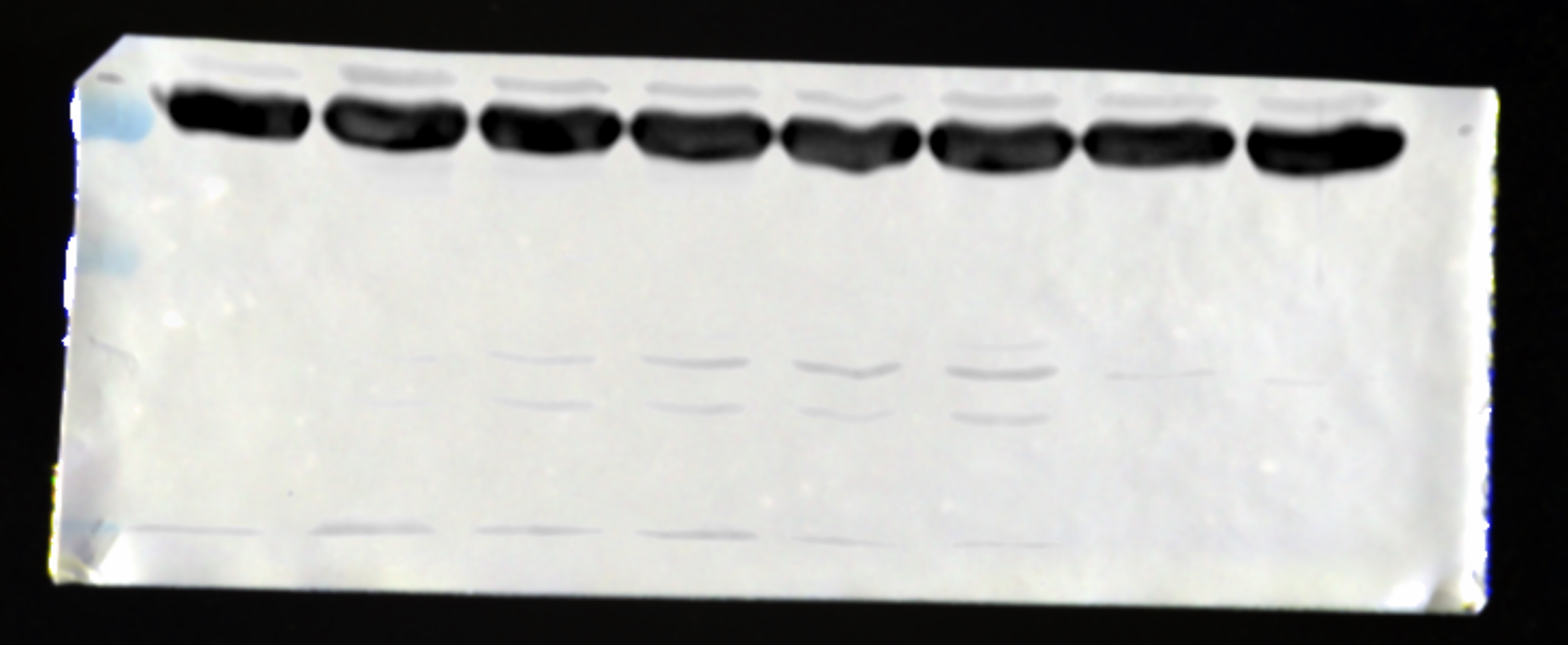

Supplement: Supplementary file 2 [file DataSheet2.zip › FigureS12_WB/FigureS11A/Tubulin/b4/23.04.18_07.58.36_ECL+Marker.tif]

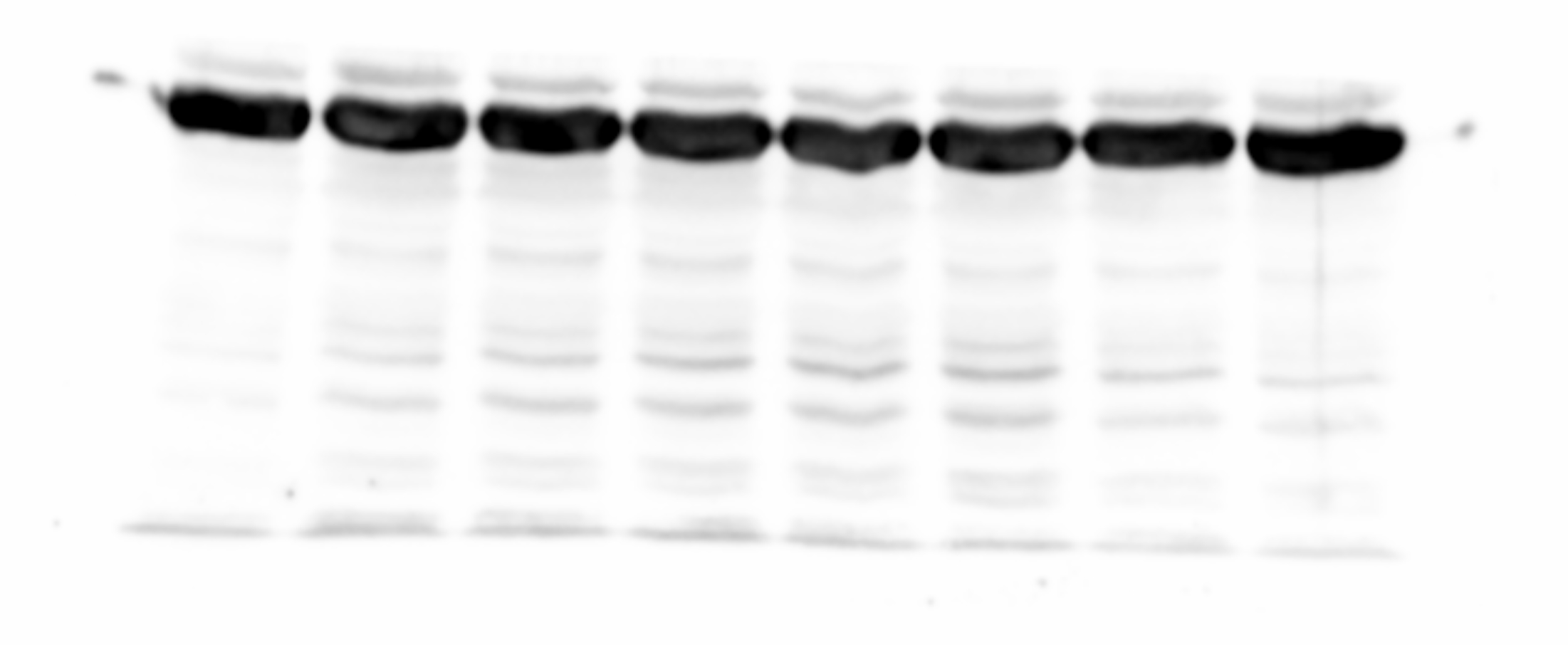

Supplement: Supplementary file 2 [file DataSheet2.zip › FigureS12_WB/FigureS11A/Tubulin/b4/23.04.18_07.58.36_ECL.tif]

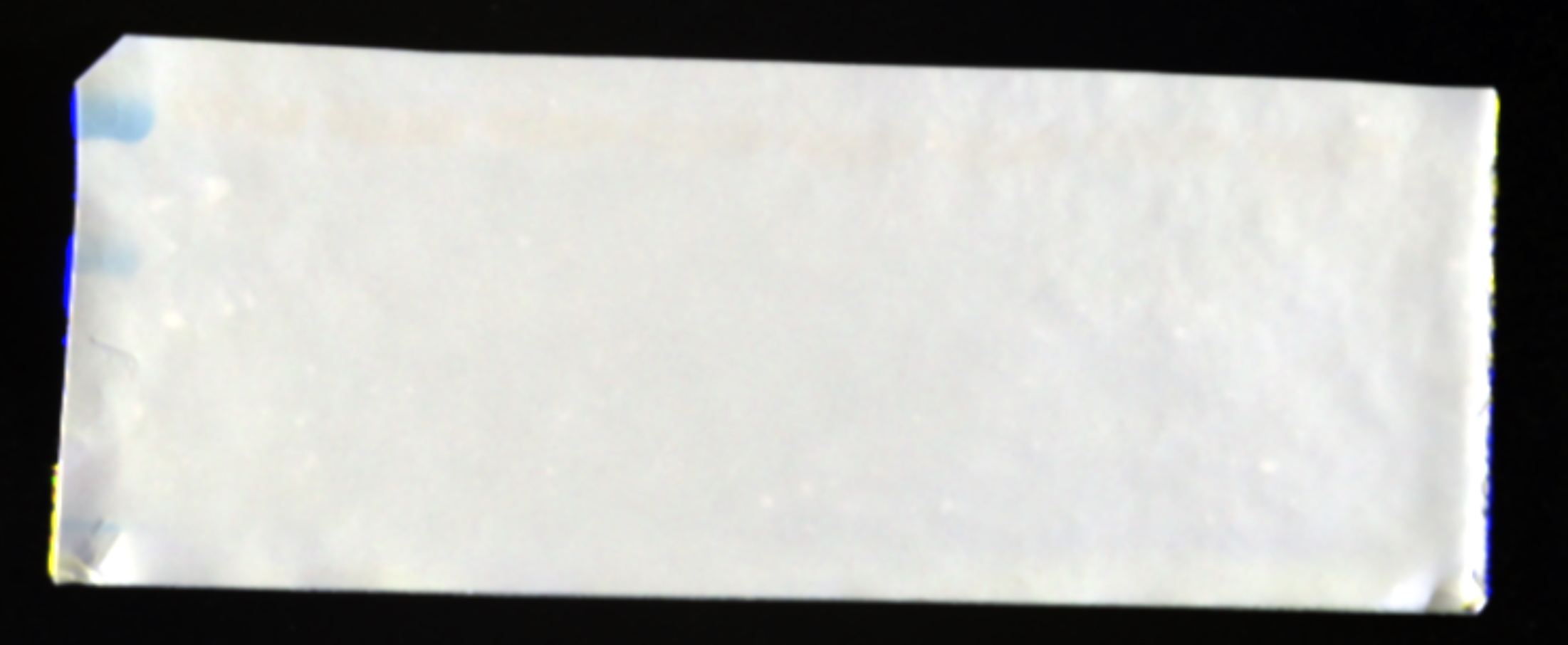

Supplement: Supplementary file 2 [file DataSheet2.zip › FigureS12_WB/FigureS11A/Tubulin/b4/23.04.18_07.58.36_marker.tif]

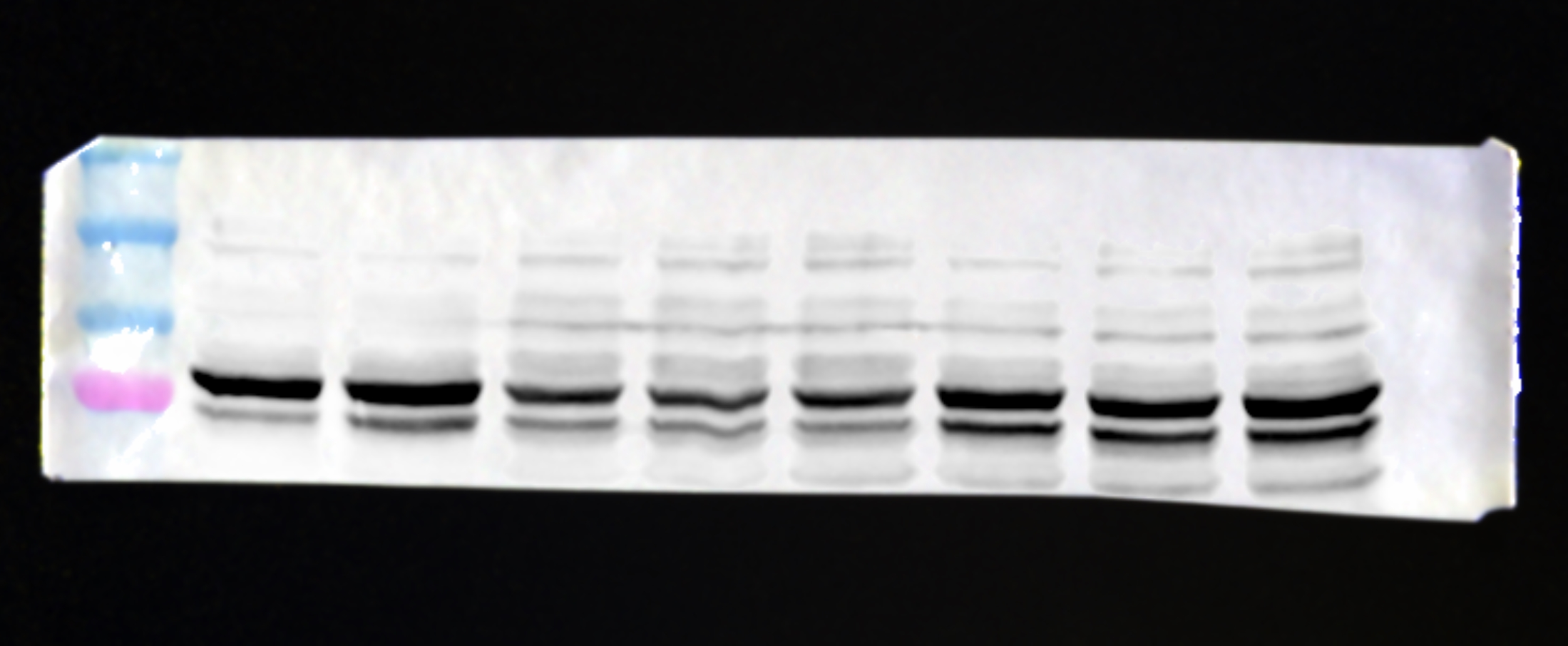

Supplement: Supplementary file 2 [file DataSheet2.zip › FigureS12_WB/FigureS11B/pSTAT3/23.04.18_08.24.09_ECL+Markerc.tif]

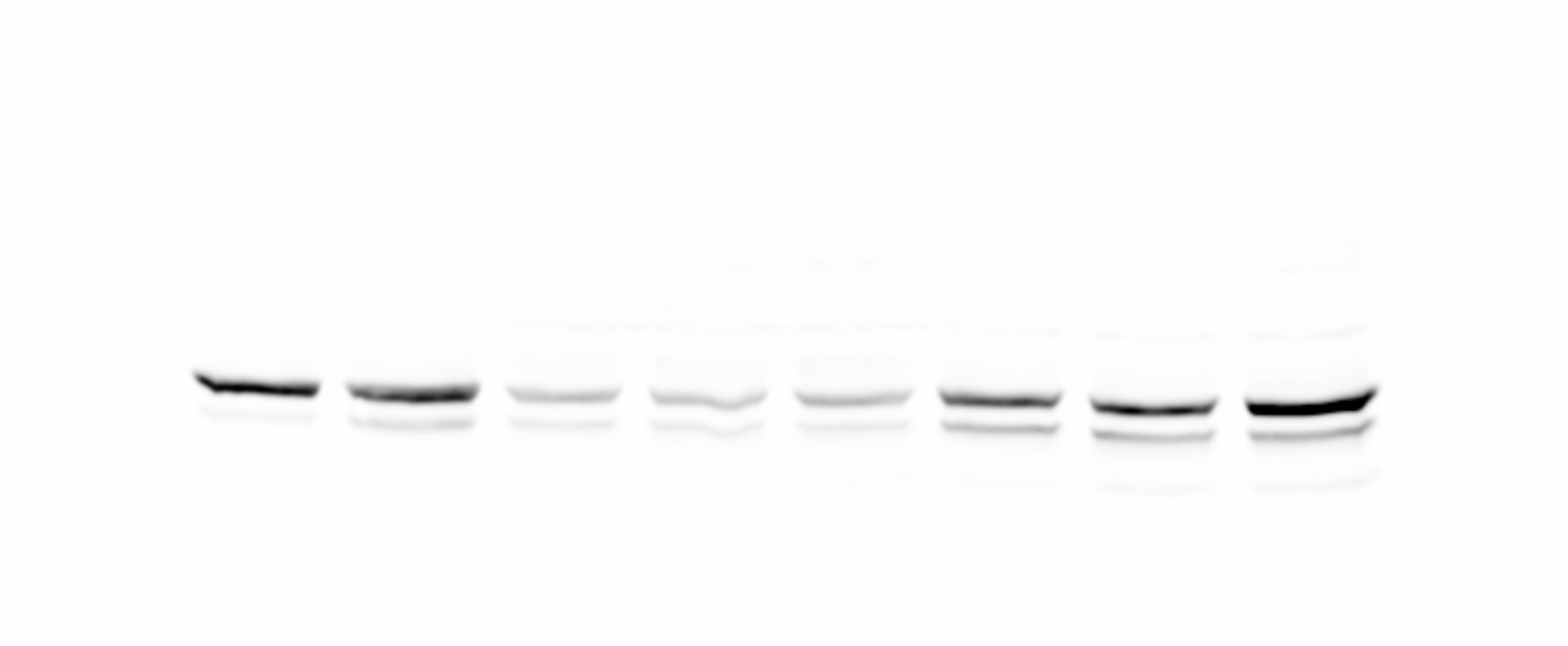

Supplement: Supplementary file 2 [file DataSheet2.zip › FigureS12_WB/FigureS11B/pSTAT3/23.04.18_08.24.09_ECL.tif]

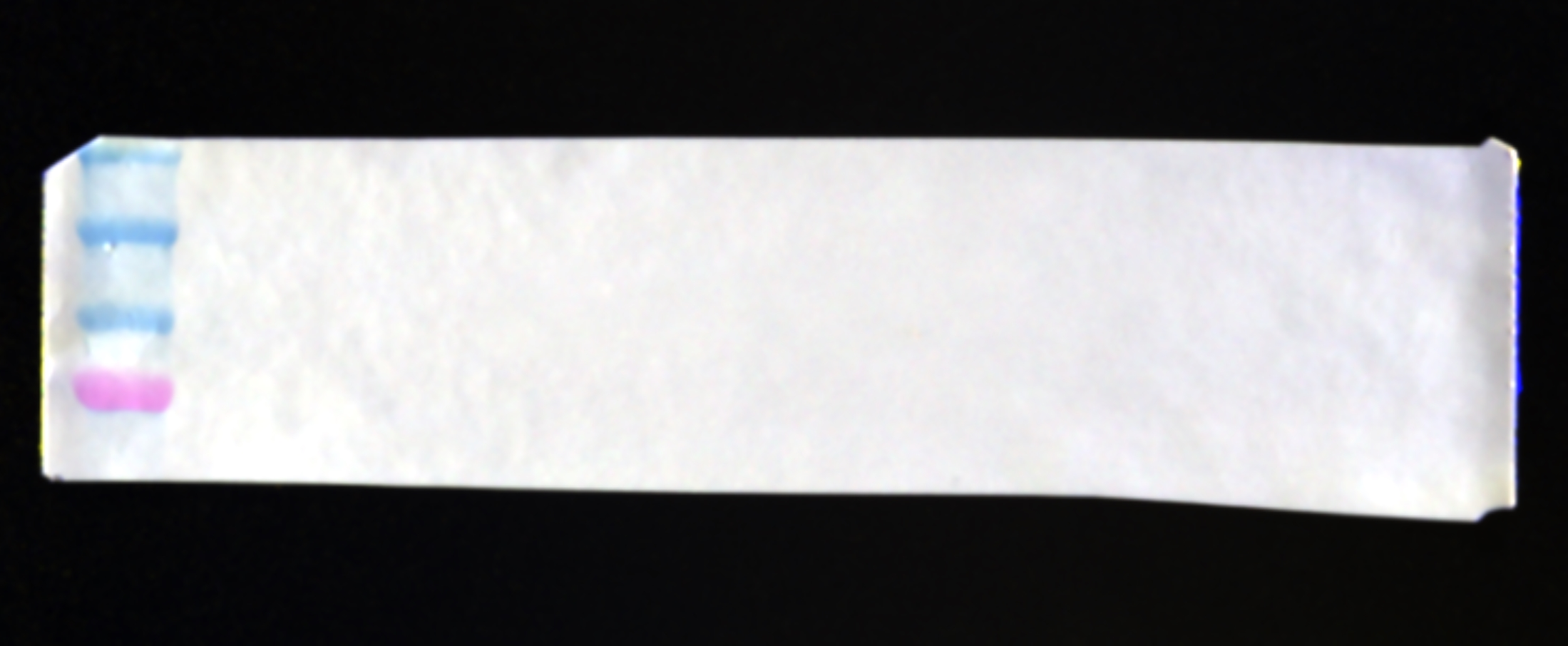

Supplement: Supplementary file 2 [file DataSheet2.zip › FigureS12_WB/FigureS11B/pSTAT3/23.04.18_08.24.09_marker.tif]

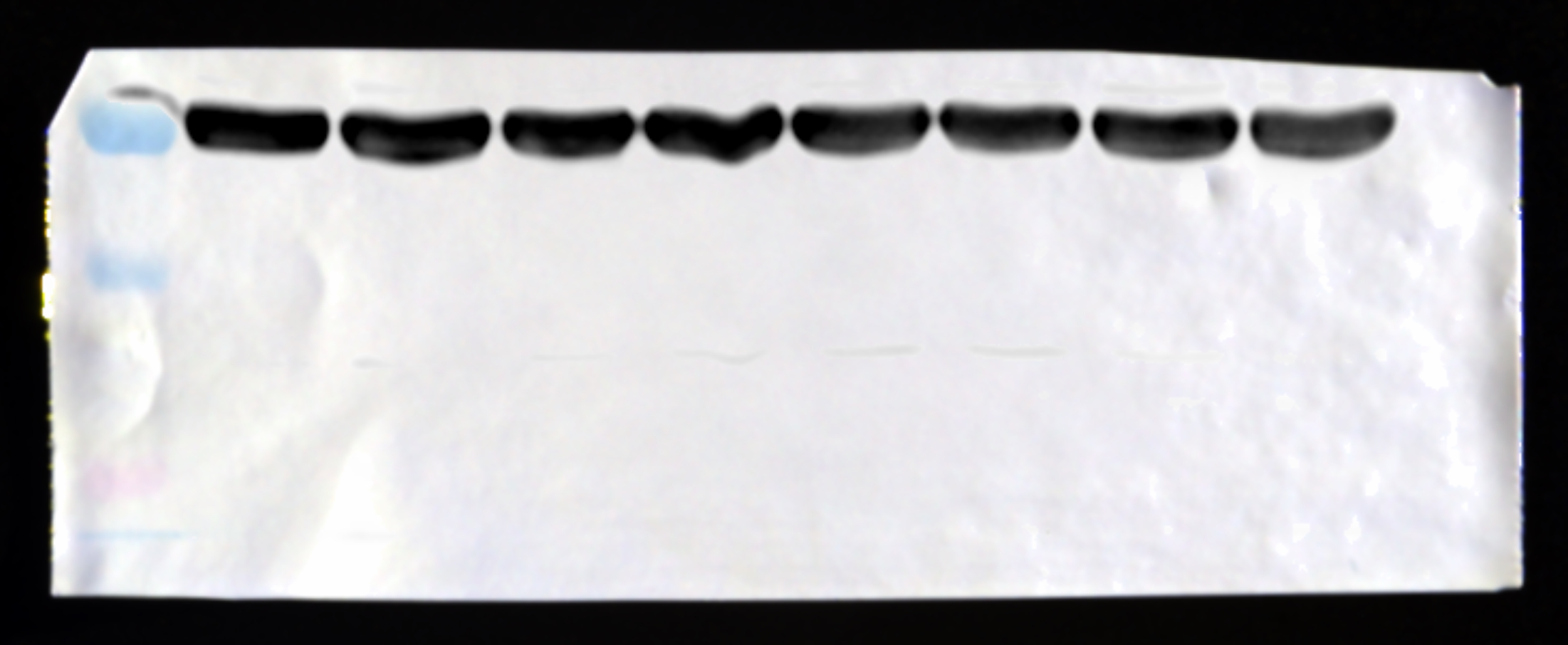

Supplement: Supplementary file 2 [file DataSheet2.zip › FigureS12_WB/FigureS11B/Tubulin/23.04.18_08.10.59_ECL+Markerc.tif]

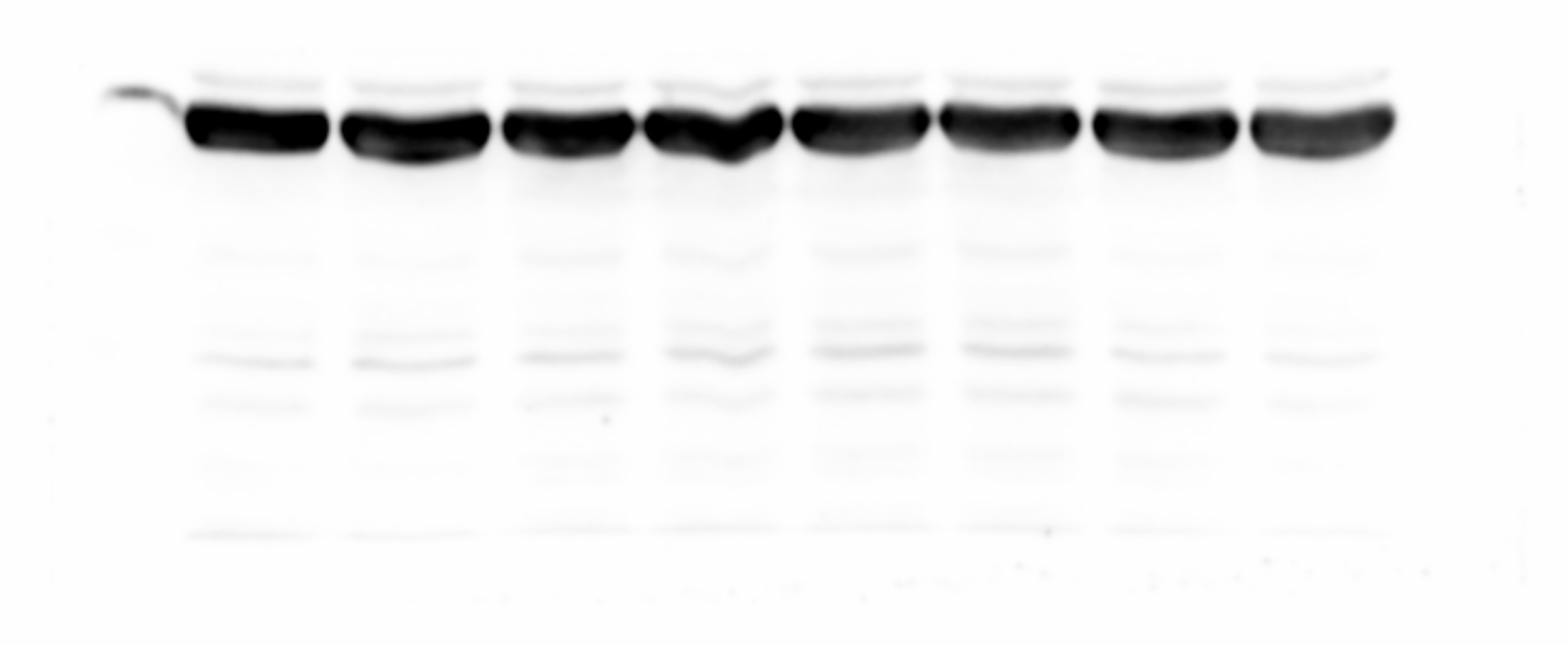

Supplement: Supplementary file 2 [file DataSheet2.zip › FigureS12_WB/FigureS11B/Tubulin/23.04.18_08.10.59_ECL.tif]

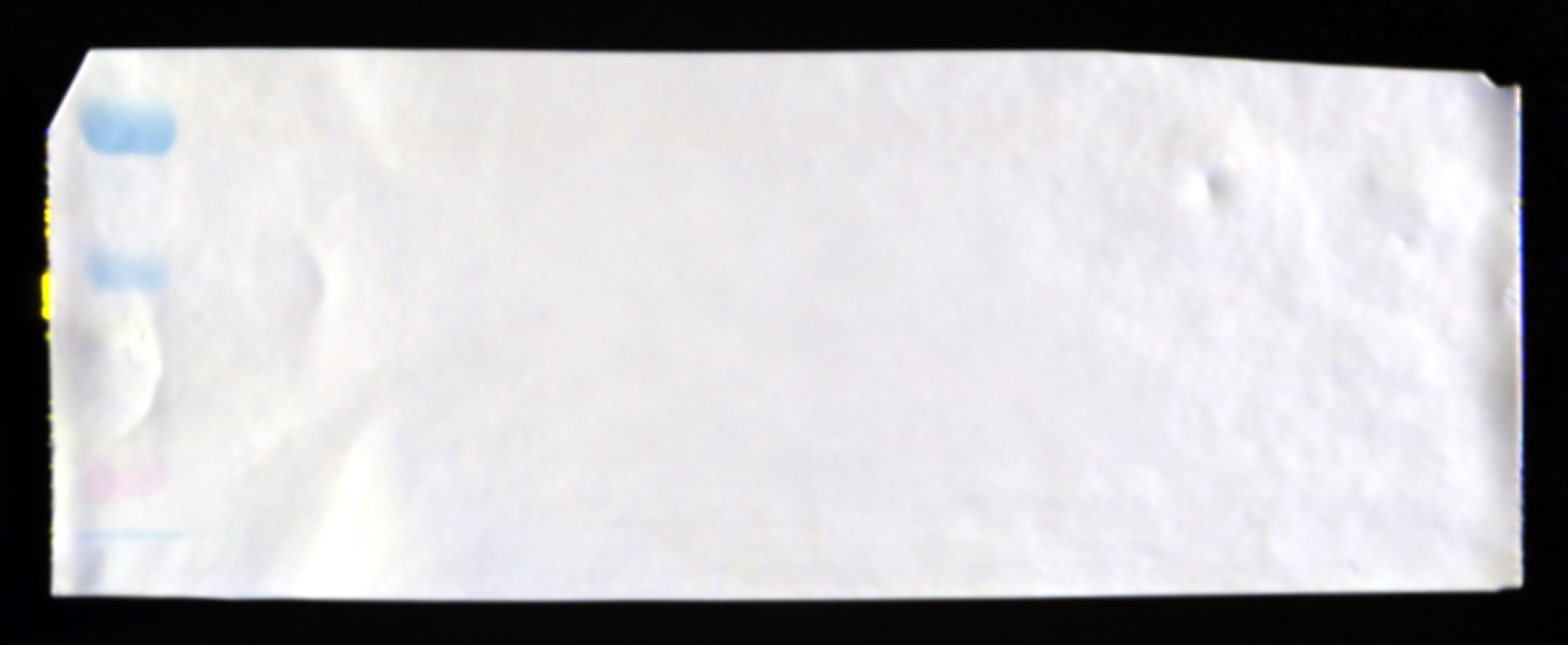

Supplement: Supplementary file 2 [file DataSheet2.zip › FigureS12_WB/FigureS11B/Tubulin/23.04.18_08.10.59_marker.tif]

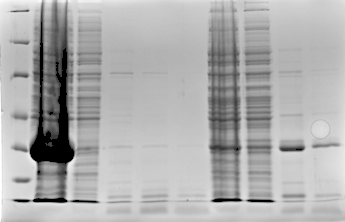

Supplement: Supplementary file 2 [file DataSheet2.zip › FigureS3_SDSPAGE/IL20R2D.tif]

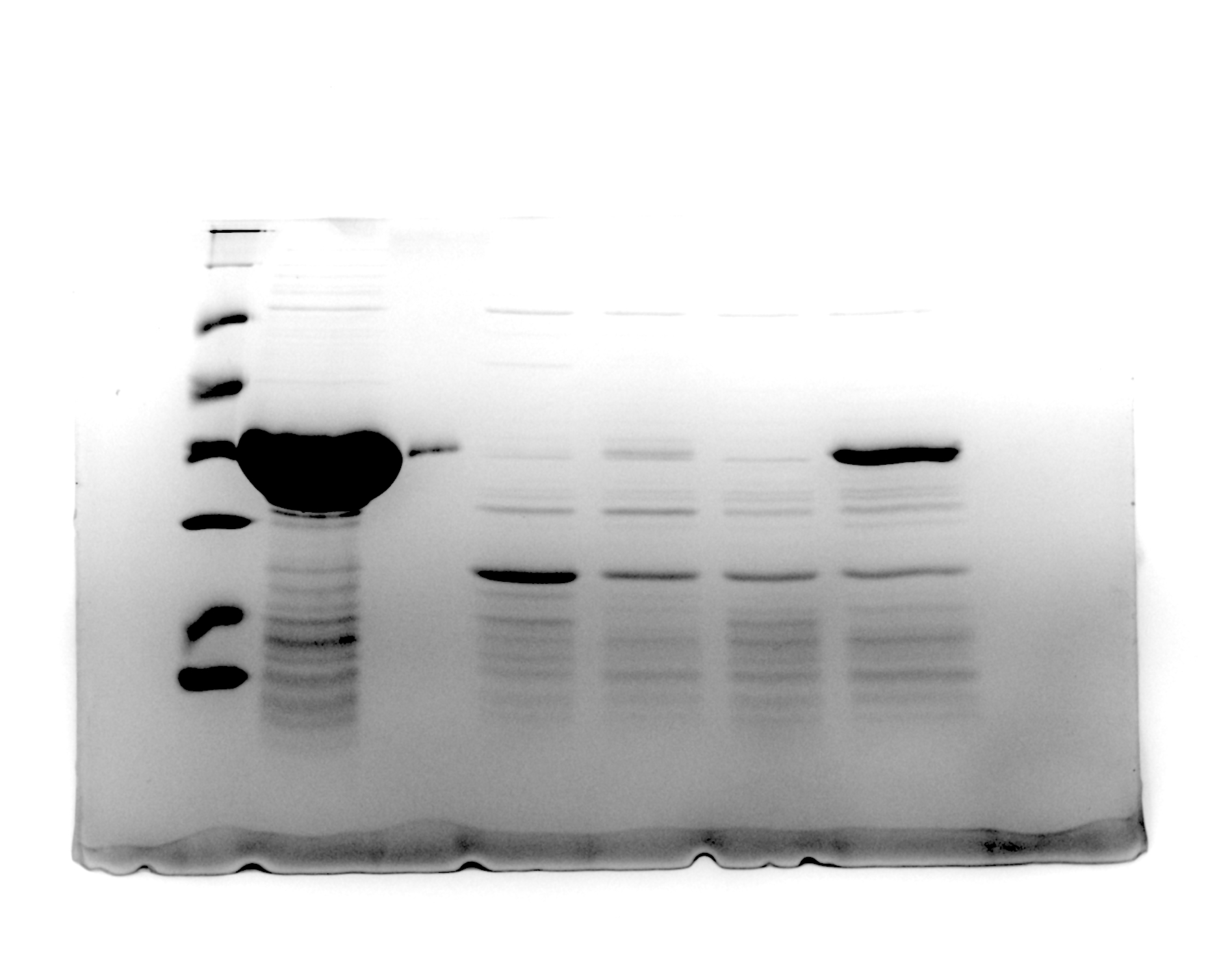

Supplement: Supplementary file 2 [file DataSheet2.zip › FigureS5_SDSPAGE/22.05.24_12.01.27_FigureS5A.tif]

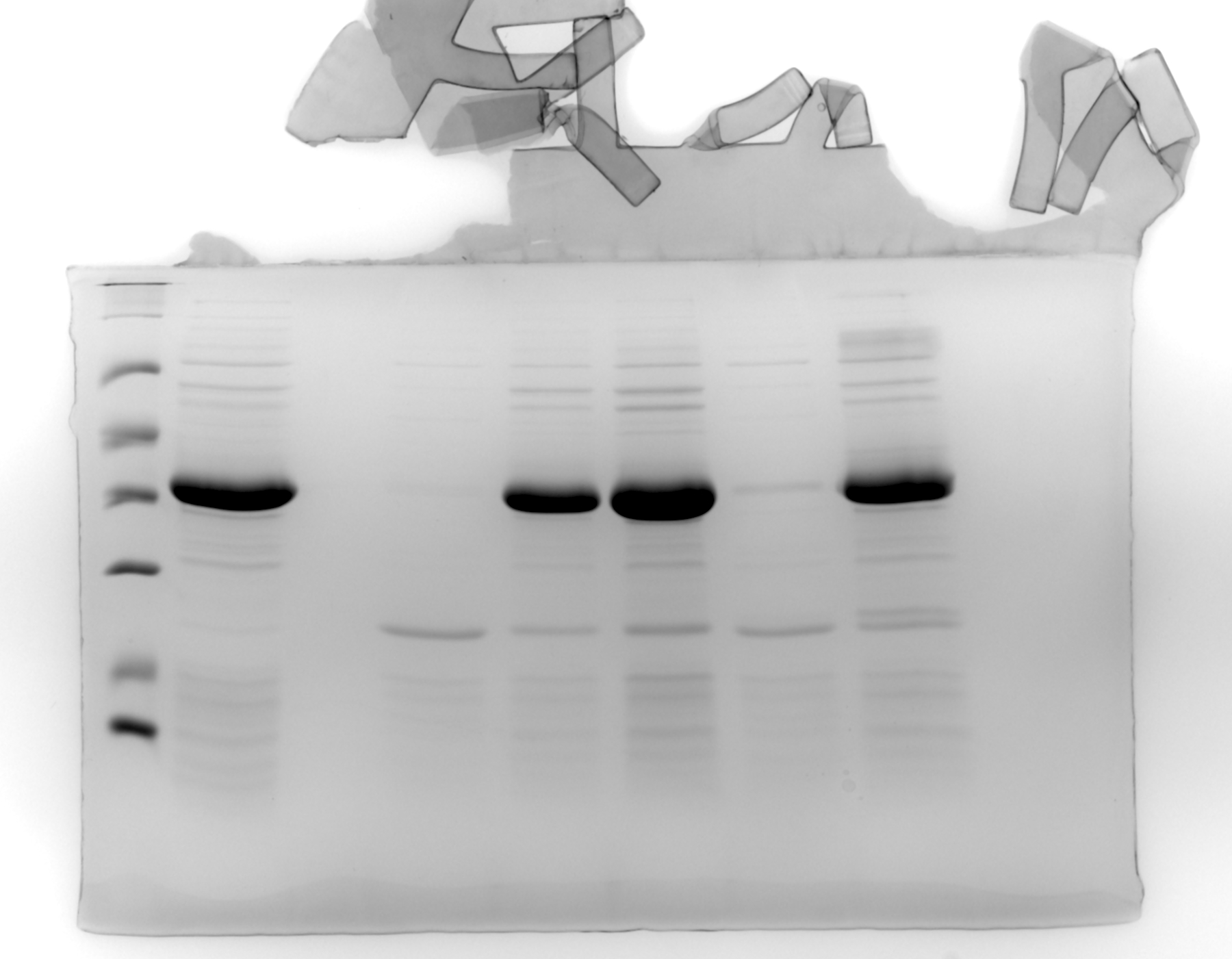

Supplement: Supplementary file 2 [file DataSheet2.zip › FigureS5_SDSPAGE/22.05.24_12.10.09_FigureS5B.tif]
